# Morphological phylogeny of *Tradescantia* L. (Commelinaceae) sheds light on a new infrageneric classification for the genus and novelties on the systematics of subtribe Tradescantiinae

**DOI:** 10.3897/phytokeys.89.20388

**Published:** 2017-10-26

**Authors:** Marco O. O. Pellegrini

**Affiliations:** 1 Universidade de São Paulo, Departamento de Botânica, Rua do Matão 277, CEP 05508-900, São Paulo, SP, Brazil; 2 Jardim Botânico do Rio de Janeiro, Rua Pacheco Leão 915, CEP 22460-030, Rio de Janeiro, RJ, Brazil; 3 Current address: Smithsonian Institution, NMNH, Department of Botany, MRC 166, P.O. Box 37012, Washington D.C. 20013-7012, USA

**Keywords:** Commelinales, *Elasis*, *Gibasis*, inflorescence morphology, Tradescantieae, spiderworts

## Abstract

Throughout the years, three infrageneric classifications were proposed for *Tradescantia* along with several informal groups and species complexes. The current infrageneric classification accepts 12 sections – with T. sect. Tradescantia being further divided into four series – and assimilates many concepts adopted by previous authors. Recent molecular-based phylogenetic studies indicate that the currently accepted sections might not represent monophyletic groups within *Tradescantia*. Based on newly gathered morphological data on the group, complemented with available micromorphological, cytological and phytochemical data, I present the first morphology-based evolutionary hypothesis for *Tradescantia*. Furthermore, I reduce subtribe Thyrsantheminae to a synonym of subtribe Tradescantiinae, and propose a new infrageneric classification for *Tradescantia*, based on the total evidence of the present morphological phylogeny, in accordance to the previously published molecular data.

## Introduction

*Tradescantia* L., as currently circumscribed, is the second largest genus of Commelinaceae, comprising ca. 90 species confined to the Neotropics and having Mexico and southern USA as its diversity center (Hunt 1975, 1980, 1986b; Faden 1998; eMonocot 2010; The Plant List 2013). The genus has been traditionally characterized by its contracted and fused back to back double-cincinni, with each cincinnus subtended by a frondose bract, actinomorphic flowers, six equal or subequal stamens, and seeds with a linear hilum (Hunt 1975, 1980, 1986b; Faden and Hunt 1991; Faden 1998; Pellegrini 2015). However, during the last century, different authors carried out a successive dismemberment of *Tradescantia*, proposing ca. 20 segregated genera (Woodson Jr. 1942; Pichon 1946; Brenan 1966; Hunt 1975, 1983), some of them still currently accepted. Since its establishment, three different infrageneric classifications have been proposed for *Tradescantia*, based on different morphological characters and conflicting taxonomic concepts (i.e. Clarke 1881; Brückner 1930; Rohweder 1956, 1969; Hunt 1975, 1980, 1986b). In addition, different authors have often recognized several informal groups or species complexes (e.g., Woodson Jr. 1942, Anderson and Woodson Jr. 1935). The current infrageneric classification for the genus (Hunt 1975, 1980, 1986b), accepts 12 sections – with T. sect. Tradescantia being further divided into four series – and assimilates many of the concepts adopted by previous authors. This classification also restructures *Tradescantia*, by reducing to sectional rank many of the previously segregated genera (e.g. *Campelia* L.C.Rich., *Cymbispatha* Pichon, *Rhoeo* Hance, *Setcreasea* K.Schum. & Sydow, *Zebrina* Schniz., etc). Furthermore, it also integrates some species complexes recognized by previous taxonomists (e.g. the *T. fluminensis* complex, and the *T. virginiana* complex).

*Tradescantia* was included by Faden and Hunt (1991), together with *Callisia* Loefl., *Gibasis* Raf., and *Tripogandra* Raf., in subtribe Tradescantiinae. Nonetheless, the subtribe was recovered as paraphyletic, due to the inclusion of *Elasis* D.R.Hunt (a member of subtribe Thyrsantheminae). Alternatively, subtribe Thyrsantheminae has been consistently recovered as polyphyletic by all morphological and molecular phylogenies so far (Evans et al. 2000, 2003; Wade et al. 2006; Burns et al. 2011; Zuiderveen et al. 2011; Hertweck and Pires 2014; Pellegrini et al. unpublished data), thus lacking any kind of micro- or macromorphological synapomorphies. Noticing the monophyly issues with both subtribes, Hertweck and Pires (2014) proposed an informal group called *Tradescantia* alliance. This group is composed by all genera of the non-monophyletic subtribes Thyrsantheminae and Tradescantiinae *s.s.*, and represents the exclusively Neotropical crown group in tribe Tradescantieae. Within the *Tradescantia* alliance, *Tradescantia* is more closely related to *Callisia*, *Elasis*, *Gibasis*, and *Tripogandra*, from which its morphological boundaries are still poorly understood (Evans et al. 2003; Wade et al. 2006; Burns et al. 2011; Zuiderveen et al. 2011; Hertweck and Pires 2014).

The age of molecular phylogenetics has shed considerable light into the understanding of relationships within *Tradescantia*, and between related genera (Evans et al. 2000; Evans et al. 2003; Wade et al. 2006; Burns et al. 2011; Hertweck and Pires 2014). Nonetheless, all available phylogenetic studies were unsuccessful in sampling the wide morphological variation, and most of the type species of the currently accepted sections and series in *Tradescantia* (Burns et al. 2011; Hertweck and Pires 2014). Despite several aspects of *Tradescantia*, such as chemotaxonomy (Martínez and Martínez 1993), anatomy (Tomlinson 1966, 1969), cytology (Jones and Jopling 1972; Martínez and Ginzo 1985), and pollen morphology (Poole and Hunt 1980) being well understood, none of these were ever considered under the light of systematics. Furthermore, all the phylogenetic studies so far, give little to no attention to morphological data. This is probably due to morphological characters being considered to be highly homoplastic in Commelinaceae, which would render them inadequate for phylogenetic inferences (Evans and Faden 1998). Nonetheless, these are the same morphological characters which were the foundation of the infrafamiliar system for Commelinaceae proposed by Faden and Hunt (1991), and which has been greatly supported by several molecular phylogenies (Evans et al. 2003; Wade et al. 2006; Burns et al. 2011; Zuiderveen et al. 2011; Hertweck and Pires 2014).

I carried out a phylogenetic analysis of *Tradescantia*, based on newly gathered macromorphological evidence, combined with the micromorphological, cytological and phytochemical data available in the literature. My goals were to: (1) test the monophyly of *Tradescantia* and its relation to *Gibasis* and *Elasis*; (2) test the current infrageneric classification for the genus; (3) provide insights into the adaptive radiation and geographical diversification of *Tradescantia*; and (4) test the hypothesis by Evans and Faden (1998), regarding the relevance of morphological data on phylogenetic analyses in Commelinaceae. Furthermore, I also explored the importance of underutilized characters to understand the phenotypic variation and morphological evolution of seed plants, using *Tradescantia* as a model group. Finally, based on the combined results of morphological studies and previously published molecular data, I propose a new infrageneric classification for *Tradescantia*, and the expansion of subtribe Tradescantiinae to correspond to the *Tradescantia* alliance as proposed by Hertweck and Pires (2014).

## Methods

### Taxon sampling

The present study samples 60 taxa from the *Tradescantia* alliance (as circumscribed by Hertweck and Pires 2014), including all four genera currently accepted for subtribe Tradescantiinae and two genera from subtribe Thyrsantheminae (i.e. *Elasis* and *Tinantia* Scheidw., out of six genera). The ingroup includes 42 species of *Tradescantia* (ca. 90 species total in the genus) and five species of *Gibasis* (11 spp.). The outgroup is represented by five species of *Callisia* (20 spp., with representatives from sections *Leptocallisia* and *Callisia*), five species of *Tripogandra* (ca. 22 spp.) and the monospecific *Elasis*. The analysis is rooted in two species of *Tinantia* (ca. 13 spp.), since the genus is consistently recovered as the first lineage to diverge in the *Tradescantia* alliance (Wade et al. 2006; Burns et al. 2011; Hertweck and Pires 2014). Type species from all sections and series currently accepted for *Tradescantia* and *Gibasis* were sampled, with the exception of the type species of *Gibasis* [i.e. *G. pulchella* (Kunth) Raf., not available for analysis when this study was carried out]. Aside from this, the sampling of this study aimed to represent the morphological and diversity centers in each genus, especially *Tradescantia*. I have studied at least five specimens for each species, with the most representative specimen chosen as the voucher (Table 1). I was also unable to sample *Weldenia* Schult.f. and *Thyrsanthemum* Pichon due to both being endemic to Mexico and represented by few specimens in most herbaria, especially in South American collections. Since both genera are phylogenetically well-placed and supported based on molecular data, and are morphologically quite distinctive from the studied genera, including them in the present analysis is dispensable, since it would only increase the degree of homoplasy and phylogenetic noise in the analysis. For the same reason, *Callisia warszewicziana* (Kunth & C.D.Bouché) D.R.Hunt, which was originally sampled, was excluded from the final analysis.

**Table 1.** Voucher specimens used in the phylogenetic analysis. ^*^Type species of the genus. ^**^Type species of the infrageneric rank.

Taxon	Infrageneric rank	Collector & no.	Herbarium
*Tinantia erecta* (Jacq.) Fenzl^*^	–	Pellegrini 315	RB
*Tinantia sprucei* C.B.Clarke	–	Santos 1149	RB
*Tradescantia fluminensis* Vell.	Sect. Austrotradescantia ^**^	Pellegrini 48	RB
*T. cerinthoides* Kunth	Sect. Austrotradescantia	Pellegrini 445	RB
*T. crassula* Link & Otto	Sect. Austrotradescantia	Pellegrini 439	RB
*T. chrysophylla* M.Pell.	Sect. Austrotradescantia	Custódio Filho 1910	RB
*T. cymbispatha* C.B.Clarke	Sect. Austrotradescantia	Pellegrini 17	RB
*T. mundula* Kunth	Sect. Austrotradescantia	Pellegrini 434	RB
*T. seubertiana* M.Pell.	Sect. Austrotradescantia	Pellegrini 436	RB
*T. tenella* Kunth	Sect. Austrotradescantia	Pellegrini 431	RB
*T. umbraculifera* Hand.-Mazz.	Sect. Austrotradescantia	Pellegrini 192	RB
*T. valida* G.Brückn.	Sect. Austrotradescantia	s.leg. s.n.	B barcode B100296487
*Tradescantia* sp. 1	Sect. Austrotradescantia	Pellegrini 207	RB
*Tradescantia* sp. 2	Sect. Austrotradescantia	Wood 21010	K
*T. zanonia* (L.) Sw.	Sect. Campelia ^**^	Pellegrini 412	RB
*T. commelinoides* Schult. & Schult.f.	Sect. Cymbispatha ^**^	Breedlove 12239	US
*T. gracilima* Standl.	Sect. Cymbispatha>	Standley 55158	F
*T. grantii* Faden	Sect. Cymbispatha	Grant 92-01801	US
*T. poelliae* D.R.Hunt	Sect. Cymbispatha	Pöll 8	K
*T. praetermissa* M.Pell.	Sect. Cymbispatha	Mandon 1237	K
*T. standleyi* Steyerm.	Sect. Cymbispatha	Steyermark 50970	US
*T. guatemalensis* C.B.Clarke *ex* Donn.Sm.	Sect. Coholomia ^**^	Heyde 3519	US
*T. soconuscana* Matuda	Sect. Corinna ^**^	Faden 76/98	US
*T. ambigua* Mart. *ex* Schult. & Schult.f.	Sect. Mandonia ^**^	Martius 140	M
*T. boliviana* (Hassk.) J.R.Grant	Sect. Mandonia	Mandon 1239	K
*T. crassifolia* Cav.	Sect. Mandonia	Rose 216	US
*T. gentryi* D.R.Hunt	Sect. Mandonia	Gentry 14415	US
*T. petricola* J.R.Grant	Sect. Mandonia	Chavarría 1035	US
*T. tepoxtlana* Matuda	Sect. Mandonia	Smith 3618	US
*T. andrieuxii* C.B.Clarke	Sect. Parasetcreasea ^**^	Andrieux 53	K
*T. spathacea* Sw.	Sect. Rhoeo ^**^	Pellegrini 499	RB
*T. virginiana* L. ^*^	Sect. Tradescantia ser. Virginianae^**^	Faden 87/1a	US
*T. occidentalis* (Britton) Smyth	Sect. Tradescantia ser. Virginianae	Shantz 1118	US
*T. sillamontana* Matuda	Sect. Tradescantia ser. Sillamontanae^**^	White 30	MICH
*T. pinetorum* Greene	Sect. Tradescantia ser. Tuberosae^**^	Greene s.n.	US barcode US00044946
*T. wrightii* Rose & Bush	Sect. Tradescantia ser. Tuberosae	Wright 701	US
*T. orchidophylla* Rose & Hemsl.	Sect. Tradescantia ser. Orchidophyllae^**^	Jones 467	US
*T. mirandae* Matuda	Sect. Tradescantia ser. Orchidophyllae	Moore 4735	US
*T. pygmaea* D.R.Hunt	Sect. Separotheca ^**^	Rose 2095	US
*T. brevifolia* (Torr.) Rose	Sect. Setcreasea ^**^	Bigelow 1500-a	NY
*T. hirta* D.R.Hunt	Sect. Setcreasea	Wagner 4114	US
*T. pallida* (Rose) D.R.Hunt	Sect. Setcreasea	Palmer s.n.	US barcode US00091625
*T. zebrina* Heyhn. *ex* Bosse	Sect. Zebrina ^**^	Pellegrini 406	RB
*T. schippii* D.R.Hunt	Sect. Zebrina	Standley 54189	US
*Elasis hirsuta* (Kunth) D.R.Hunt	–	Bonpland 2160	P
*Gibasis geniculata* (Jacq.) Rohweder	Sect. Heterobasis ^**^	Pellegrini 338	RB
*G. oxacana* D.R.Hunt	Sect. Heterobasis	Hunt 8175	K
*G. consobrina* D.R.Hunt	Sect. Gibasis	Pringle 6723	US
*G. pellucida* (M.Martens & Galeotti) D.R.Hunt	Sect. Gibasis	Pellegrini 5	RFA
*G. karwinskyana* (Schult. & Schult.f.) Rohweder	Sect. Gibasis	Pringle 9250	US
*Callisia repens* (Jacq.) L. ^*^	Sect. Callisia ^**^	Pellegrini 284	RB
*C. fragrans* (Lindl.) Woodson	Sect. Callisia	Acevedo-Rodríguez 3805	US
*C. gentlei* Matuda	Sect. Callisia	Carauta 4272	RB
*C. monandra* (Sw.) Schult. & Schult.f.	Sect. Leptocallisia ^**^	Pellegrini 430	RB
*C. filiformis* (M.Martens & Galeotti) D.R.Hunt	Sect. Leptocallisia	Sobral-Leite 814	RB
*Tripogandra multiflora* (Sw.) Raf. ^*^	–	Swartz s.n.	BM barcode BM000578859
*T. diuretica* (Mart.) Handlos	–	Pellegrini 4	RFA
*T. elata* D.R.Hunt	–	C.A. Ferreira Junior s.n.	RB barcode RB00821839
*T. glandulosa* (Seub.) Rohweder	–	Pellegrini 298	RB
*T. warmingiana* (Seub.) Handlos	–	Pellegrini 346	RB

### Character selection

**Figure 1.**
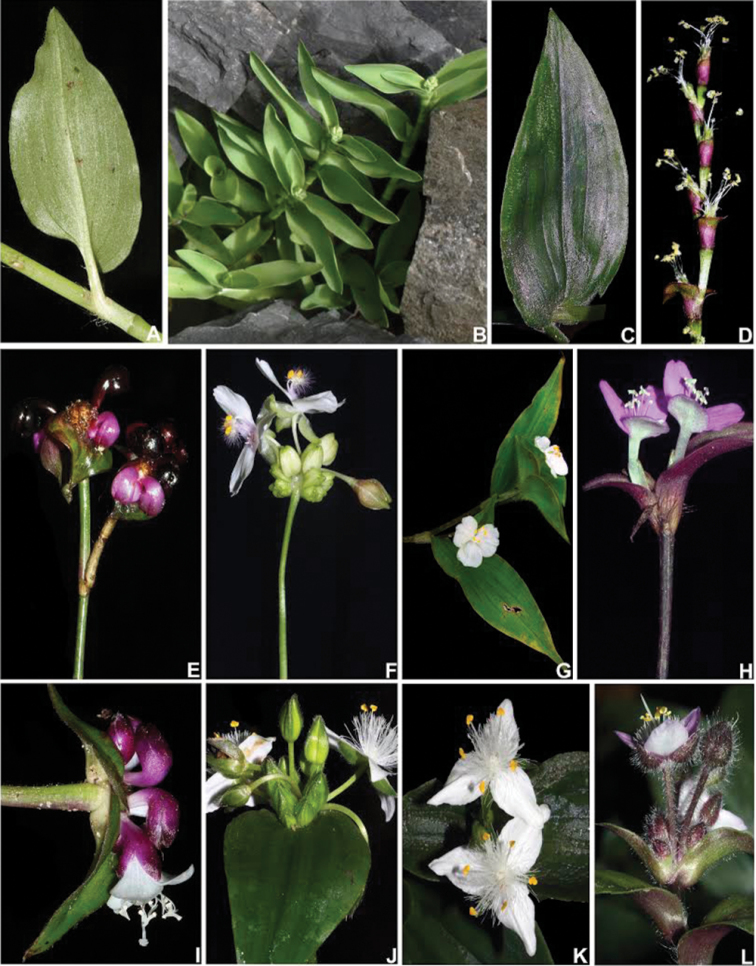
Some macromorphological characters used in the phylogenetic analysis. **A** subpetiolate leaf (Character 8) and asymmetrical base (Character 16), in *Tradescantia tenella* Kunth **B** complicate leaves (Character 8), in *Tradescantia crassula* Link & Otto. **C** impressed secondary veins (Character 19), in *Tradescantia fluminensis* Vell **D** predominantly axillar to thyrse-like synflorescence (Character 24), in *Callisia repens* (Jacq.) L. **E** synflorescence with two paraclades (Character 26), in *Tradescantia zanonia* (L.) Sw. **F** contracted cincinni (Character 34), fused back to back (Character 35), vestigial cincinni bracts (Character 38), flower display of 60° (Character 48), shorter antesepalous stamens (Character 72), sigmoid filaments (Character 73), and zygomorphic androecium (Character 76), in *Tripogandra diuretica* (Mart.) Handlos **G** supernumerary cincinni bracts (Character 37), in *Tradescantia praetermissa* M.Pell **H** cincinni bracts saccate at base (Character 43), tubular flower (Character 47), fused petals (Character 60), clawed petals (Character 62), shorter antesepalous stamens (Character 72), connective expanded and transversally linear (Characters 77–80), round anther sacs (Characters 81–82), pollen white *in vivo* (Character 83), and trilobate stigma (Character 91), in *Tradescantia zebrina* Heynh. *ex* Bosse. **I** tubular flower (Character 47), pedicels geniculate at anthesis and pre-anthesis (Character 51), fused sepals (Character 53), filaments bearded with sparse and short hairs at mid-length (Characters 66–71), shorter antesepalous stamens (Character 72), connective expanded and transversally linear (Characters 77–80), round anther sacs (Characters 81–82), pollen white *in vivo* (Character 83), and trilobate stigma (Character 91), in *T. zanonia* **J** sepals all keeled (Character 56), in *T. fluminensis* **K** filaments basally bearded with dense and long hairs (Characters 66–71), connective expanded and rhomboid (Characters 77–80), anther sacs ellipsoid (Characters 81–82), and pollen yellow *in vivo* (Character 83), in *T. fluminensis* **L** pistil longer than the androecium (Character 86) and punctate (Character 91), in *Tradescantia cerinthoides* Kunth. All photos by M.O.O. Pellegrini, except G by H. Huaylla.

**Figure 2.**
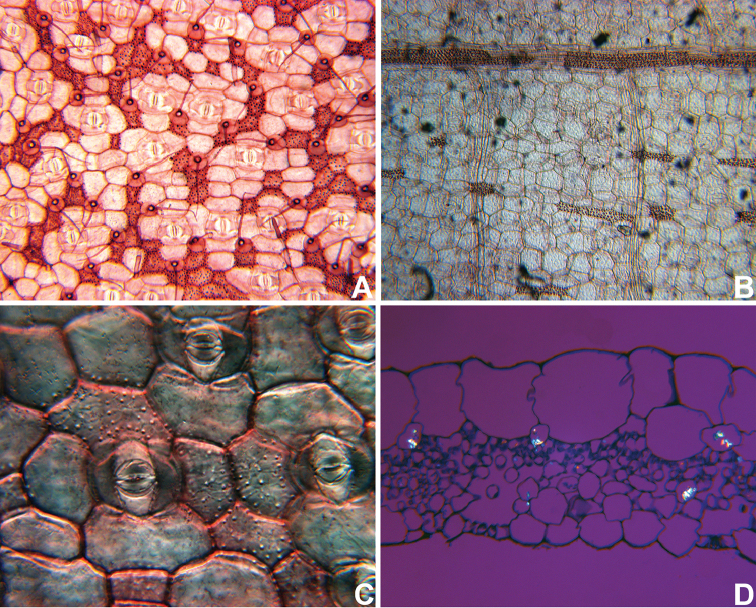
Anatomical characters used in the phylogenetic analysis. **A** leaf epidermis with silica crystals in specialized cells with thickened cell walls (Character 103–104), in *Callisia multiflora* (M.Martens & Galeotti) Standl **B** leaf epidermis with silica crystals in specialized cells without thickened cell walls (Character 103–104), bundle sheath in the mesophyll with longitudinal sclerenchymatic extensions (Character 105), in *Gibasis pellucida* (M.Martens & Galeotti) D.R.Hunt. **C** detail of the silica crystals in the leaf epidermis, in *Tripogandra aff. glandulosa* **D** raphides inside the raphide canals, evidencing the different morphology and position from the silica crystals in the leaf epidermis, in *G. pellucida*. All photos by S. Yankowski & F.B. Faden; **A** based on *Spencer 92-308* (US), **B, D** based on *Rosen 4645* (US), **C** based on *Bogner 2381* (US).

Characters were scored mainly from living specimens at the field and specimens kept at the Jardim Botânico do Rio de Janeiro greenhouses, and later complemented by spirit and herbarium samples from the following herbaria: ALCB, B, BA, BHCB, BHZB, BM, BOTU, BRIT, C, CAL, CEPEC, CESJ, CGE, CGMS, CNMT, COR, CORD, CVRD, EAC, ESA, F, FCAB, FCQ, FLOR, FUEL, FURB, GUA, HAMAB, HAS, HB, HBR, HDCF, HRB, HRCB, HSTM, HUCS, HUEFS, HUFSJ, HURB, IAC, ICN, INPA, JOI, K, L, MBM, MBML, MG, MO, MY, NY, P, PACA, PMSP, R, RB, RFA, RFFP, SCP, SP, SPF, SPSF, U, UEC, UFRN, UPCB, US, W, WAG, and WU (herbaria acronyms according to Thiers, continuously updated). Many additional specimens were examined during collections made on expeditions in Brazil and in the USA, between 2010–2016. When living or herborized specimens were not available for examination, information was taken from published literature (Table 2). A limited number of characters used here have been analyzed in previous studies (i.e. Evans et al. 2000; Panigo et al. 2011), with most characters being coded and scored for the first time in the present study. Character coding followed the recommendations of Sereno (2007) for morphological phylogenies. Primary homology hypotheses (De Pinna 1991) were proposed for root, stem, leaf, inflorescence architecture, floral, fruit, seed (partly illustrated in Fig. 1), palynological, anatomical characters (illustrated in Fig. 2), phytochemical and cytological characters. A total of 114 discrete characters were scored, being: 97 macromorphological, three micromorphological, two palynological, three anatomical, four cytological, and five phytochemical (Suppl. material 1). All the characters were treated as unordered and were equally weighted. The terminology for indumentum and shapes follows Radford et al. (1974); inflorescence terminology follows Weberling (1965, 1989) and Panigo et al. (2011); stigmatic micromorphology terminology follows Owens and Kimmins (1981) and Owens et al. (1984); fruit terminology follows Spjut (1994); seed terminology follows Faden (1991); phytochemical terminology follows Martínez and Martínez (1993); cytology terminology follows Jones and Jopling (1972) and Martínez and Ginzo (1985); pollen terminology follows Poole and Hunt (1980); anatomical terminology follows Tomlinson (1966, 1969); and general macromorphological terminology follows Pellegrini (2015).

**Table 2.** Primary literature sources for information used in the phylogenetic analysis.

Character	Source
Pollen	Poole and Hunt 1980
Stigmatic micromorphology	Owens and Kimmins 1981; Owens et al. 1984
Anatomy	Tomlinson 1966, 1969
Cytology	Anderson and Sax 1936; Jones and Jopling 1972; Jones et al. 1981; Martínez 1984; Martínez and Ginzo 1985; Jones 1990
Phytochemistry	Martínez and Sawin 1985; Martínez and Martínez 1993

### Phylogenetic analysis

Data was entered into a matrix of characters per taxa using the software Mesquite 3.20 (Maddison and Maddison 2017; Suppl. material 2). Maximum Parsimony (MP) analysis was performed using PAUP* 4 (Swofford 2003), with a heuristic search with 1000 random taxon additions and tree bisection-reconnection (TBR) branch swapping. Consistency index (CI) and retention index (RI) were used to assess the degree of homoplasy in the dataset, and using character optimization of ACCTRAN (accelerated transformation optimization; Swofford and Maddison 1987). Statistical support for each branch of the cladogram was evaluated with Bootstrap Support (BS) analyses with 1000 random addition replication. The search parameters used to estimate the bootstrap values were the same as the initial heuristic search. Bremer Index (BI) was also used to evaluate clade reliability based on the presence of secondary homologies (Bremer 1994). Bremer Index was calculated by increasing the number of the optimal tree steps until all clades collapsed. Mesquite 3.20 was used to reconstruct the ancestral character states, while WinClada ver. 1.0000 (Nixon 2002) was used to trace the synapomorphic characters on the majority-rule (50% values) and strict consensus trees. The complete data matrix and trees are available at TreeBase (http://purl.org/phylo/treebase/phylows/study/TB2:S21372).

## Results

**Figure 3.**
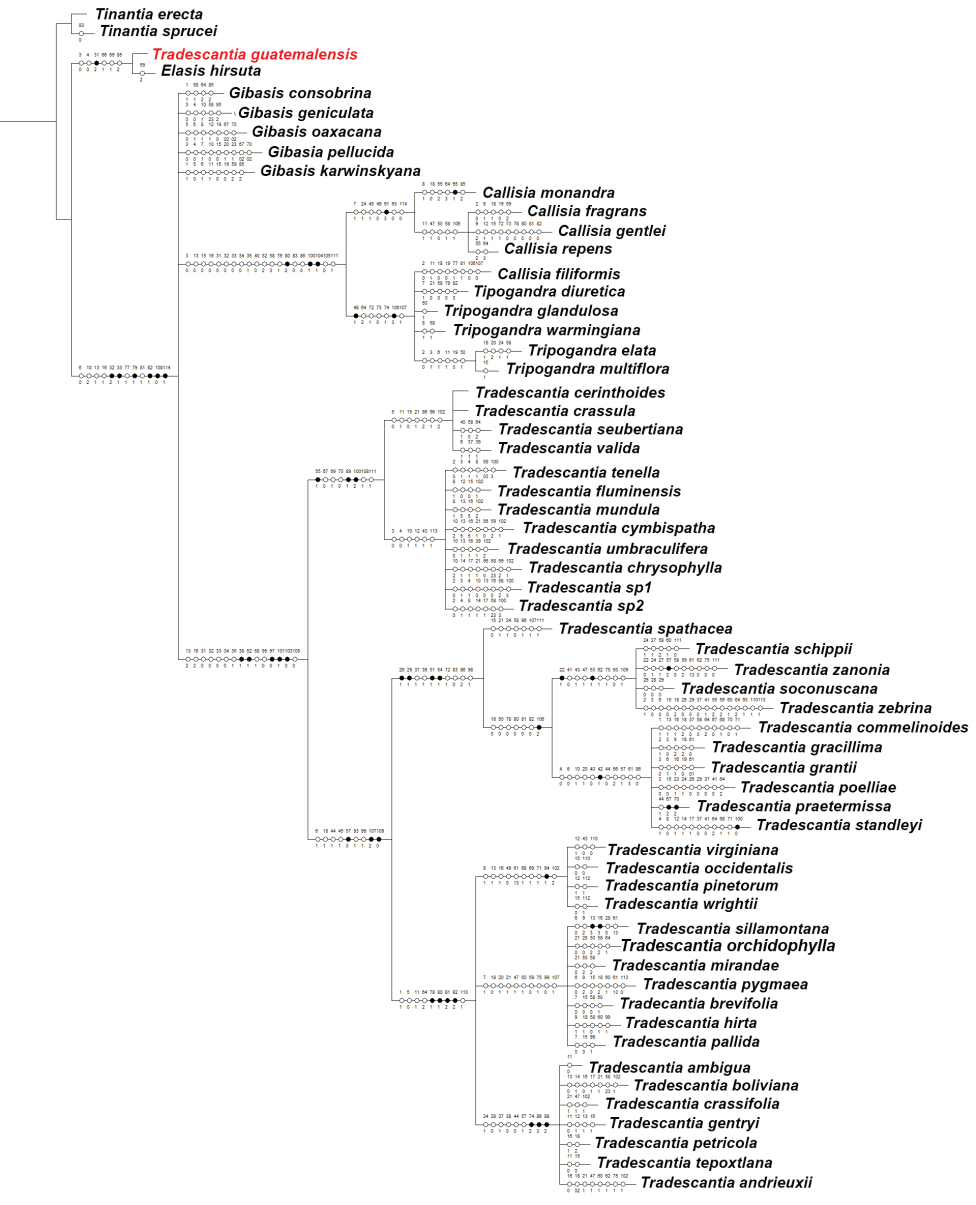
Strict consensus tree (length= 516 steps; CI= 0.3411; RI= 0.8039), showing the character state optimizations at each node of the cladogram, represented by circles. In each circle, the numbers above and below represent the character and character state numbers, respectively (as presented in Suppl. material 1). *Tradescantia guatemalensis* C.B.Clarke *ex* Donn.Sm. is depicted in red, to highlight its placement as sister to *Elasis hirsuta* (Kunth) D.R.Hunt.

**Figure 4.**
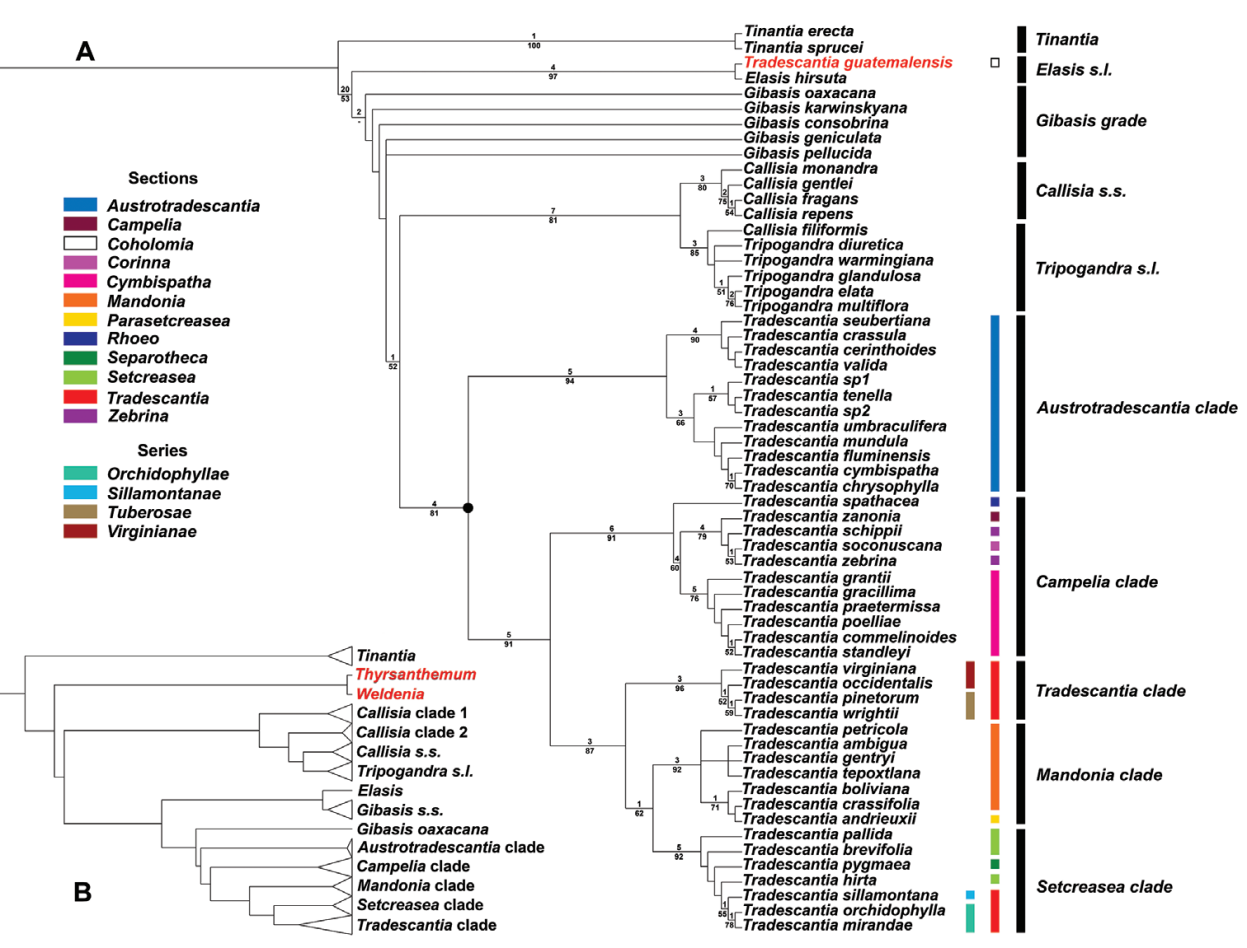
Congruence between morphological and molecular datasets. **A**, majority-rule tree showing the sections and series proposed by Hunt (1975, 1980, 1986b) color-coded; the five newly proposed subgenera are represented by the black bars; *Tradescantia guatemalensis* C.B.Clarke *ex* Donn.Sm. is depicted in red, to highlight its placement as sister to *Elasis hirsuta* (Kunth) D.R.Hunt; the ● represents *Tradescantia* *s.s.*; Bremer Index support values are depicted over the branches, while bootstrap support values are depicted under the branches **B** simplification of the current hypothesis for phylogenetic relationships in tribe Tradescantieae, based on molecular data. *Thyrsanthemum* Pichon and *Weldenia* Schult.f. are depicted in red since they were not sampled in the present study. Modified from Hertweck and Pires (2014).

**Figure 5.**
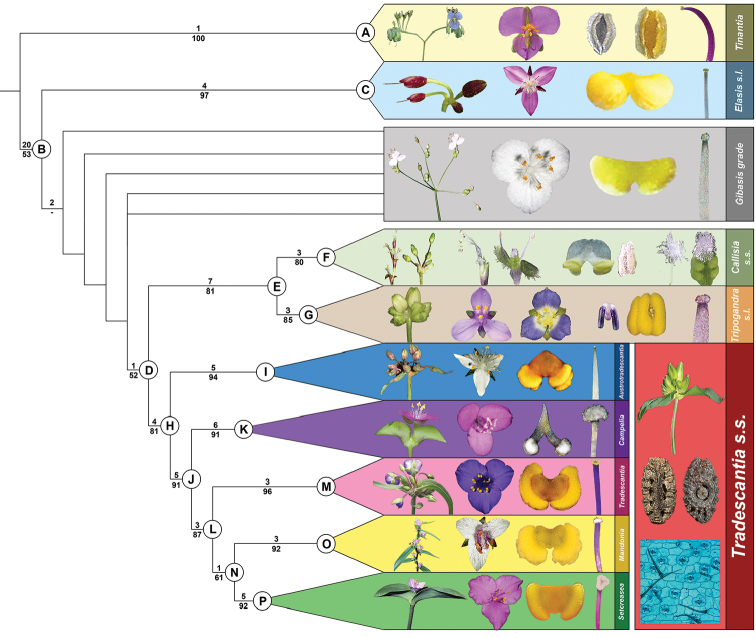
Majority-rule tree showing the relation between the genera in the *Tradescantia* alliance, highlighting the reproductive synapomorphies recovered for each lineage. Synapomorphies for *Tinantia* (clade **A**): cincinni verticillate, flowers zygomorphic, filaments sigmoid, anthers dimorphic with inconspicuous connectives, style sigmoid, stigma truncate, and hilum C-shaped. Synapomorphy for clade **B**: basal bract inconspicuous and tubular. Synapomorphies for *Elasis* *s.l.* (clade **C**): cincinni fasciculate, flowers actinomorphic, filaments sigmoid, anthers dimorphic, style sigmoid, and stigma truncate. Synapomorphies for clade **D**: double-cincinni fused back to back. Synapomorphies for clade **E**: seeds triangular to round triangular or tetrahedral, ventrally ridged, hilum elliptic or punctate, and leaf epidermis with silica crystals in specialized cells with thickened cell walls. Synapomorphies for *Callisia* *s.s.* (clade **F**): inflorescences mainly axillary, bracteoles conspicuous, pedicels apically non-gibbous, and penicilliform stigma. Synapomorphies for *Tripogandra* *s.l.* (clade **G**): floral display with a 60° torsion, petals ranging from pink to lilac to purple, antesepalous filaments shorter than the antepetalous, and filaments sigmoid at anthesis and post-anthesis. Synapomorphies for *Tradescantia* *s.s.* (clade **H**): double-cincinni fused back to back; frondose cincinni bracts, pedicels deflexed at post-anthesis, seeds elliptic to oblong in outline, ventrally flattened, hilum linear and longer than ½ the length of the seed, leaf epidermis lacking silica bodies in specialized cells, and diffuse bundle sheath in the mesophyll. Synapomorphies for T. subg. Austrotradescantia (clade **I**): sepals elliptic to broadly elliptic, all keeled, filaments basally and densely bearded with long moniliform hairs, style obconic at base and conic at apex, and stigma punctate with type D papillae. Synapomorphies for clade **J**: overlapping cincinni bracts, conspicuous bracteoles sometimes completely involving the cincinnus, membranous sepals, stigmatic papillae equal or shorter than 1μm, and conspicuous embryotega. Synapomorphies for T. subg. Campelia (clade **K**): synflorescence with one or more coflorescences, presence of peduncle bracts, presence of supernumerary bracts, spathaceous cincinni bracts, flowers with pedicels geniculate at anthesis and pre-anthesis, unequal sepals, androecium with filaments from external series shorter than the internal, pollen white *in vivo*, pistil longer than the stamens, and semilateral embryotega. Synapomorphies for Core T*radescantia* (clade **L**): petals ranging from lilac to purple or pink, connectives quadrangular to rectangular to slightly curved, anther sacs C-shaped, and pistil the same length as the stamens. Synapomorphies for T. subg. Tradescantia (clade **M**): pedicels apically non-gibbous, filaments densely bearded with moniliform hairs, and stigmatic papillae restricted to the margins of the stigma. Synapomorphies for clade **N**: ovary pubescent with eglandular hairs; hilum shorter than ½ the length of the seed. Synapomorphies for T. subg. Mandonia (clade **O**): inflorescences mainly axillary, sessile main florescences, the presence of supernumerary bracts, reduced cincinni bracts, cincinni bracts not overlapping, chartaceous sepals, filaments apically spirally-coiled at post-anthesis, style ½ time longer than the stamens, and style spirally-coiled at post-anthesis. Synapomorphies for T. subg. Setcreasea (clade **P**): saccate cincinni bracts, tubular flowers, pedicel the same length as the floral buds, hyaline sepals, fused petals, clawed petals, and epipetalous stamens.

The cladistic analysis retrieved 10,408 equally parsimonious trees with 516 steps, Consistency Index (CI) of 0.3411, Retention Index (RI) of 0.8039, and Rescaled Consistency Index (RC) of 0.2742. Out of the 114 studied characters, 113 were parsimony-informative. The strict consensus (Fig. 3) and the majority-rule trees are presented and discussed below (Fig. 4A). The monophyly of *Tradescantia*, in its current circumscription, is not supported by the present analysis, due to the position of *T. guatemalensis* C.B.Clarke *ex* Donn.Sm. (the sole member of T. sect. Coholomia) as sister to *Elasis hirsuta* (C.B.Clarke) D.R.Hunt (BS= 98; BI= 4) (Figs 3, 4A, 5 clade C). This relation is supported by their prostrate herbaceous stems (Characters 3 and 4, homoplastic), densely branched stems (Character 5, homoplastic), asymmetric leaf base (Character 19, homoplastic), cincinni arranged side by side (Character 31), filaments densely barbate (Characters 66 and 69, homoplastic), and glandular-pubescent ovary (Character 85, homoplastic) (Figs 3, 4A, 5 clade H). With the exclusion of *T. guatemalensis*, the synapomorphies supporting the *Tradescantia* *s.s.* clade (BS= 81; BI= 4) are: plants with a definite base (Character 2, homoplastic); the combination of opposite, sessile, straight, contracted and fused cincinni (Characters 31–35, homoplastic); frondose cincinni bracts (Character 37), pedicels deflexed at post-anthesis (Character 52); seeds elliptic to oblong in outline (Character 95), ventrally flattened (Character 97), and with a linear hilum (Character 101), longer than ½ the length of the seed (Character 102, homoplastic); leaf epidermis lacking silica bodies in specialized cells (Character 103), and diffuse bundle sheath in the mesophyll (Character 105, homoplastic).

### Ingroup taxa

*Gibasis* is recovered as polyphyletic, with all sampled species recovered in a polytomy in the strict consensus (Fig. 3), or placed in a basal grade in the majority-rule (Figs 4A, 5). In the strict consensus, *Tripogandra* (*sensu* Handlos 1975) was recovered as paraphyletic (BS= 80; BI= 3), due to the inclusion of *C. filiformis* (M.Martens & Galeotti) D.R.Hunt (Fig. 3). However, in the majority-rule, *Tripogandra* is recovered as monophyletic, with *C. filiformis* sister to it, but without statistical support (Figs 4A, 5 clade G). The *Tripogandra* *s.l.* clade (i.e. including *C. filiformis*) is supported by a 60° torsion in the floral display (Character 48), petals ranging from pink to lilac to purple (Character 64, homoplastic), antesepalous filaments shorter than the antepetalous (Character 72, homoplastic), filaments sigmoid at anthesis (Character 73, homoplastic), filaments sigmoid at post-anthesis (Character 74, homoplastic), chromosome count of *n*= 8 (Character 106), and medium-sized chromosomes (Character 107, homoplastic) (Figs 3, 4A, 5 clade G). Consequently, *Callisia* is also recovered as paraphyletic (BS= 85; BI= 3), due to the inclusion of *C. filiformis* in *Tripogandra*. *Callisia* *s.s.* is supported in the present analysis by: leaves congested at the apex of the stems (Character 7, homoplastic), inflorescences mainly axillary, producing a raceme-like synflorescence (Character 24, homoplastic), bracteoles conspicuous (Character 45, homoplastic), pedicels apically non-gibbous (Character 49, homoplastic), penicilliform stigma (Character 91), stigmatic papillae longer than 1μm (Character 93, homoplastic), and by the absence of sulphated phenolic acids (Character 114, homoplastic). In the strict consensus, the clade composed by *Tripogandra* *s.l.*+*Callisia* (BS= 90; BI= 8) is supported by a combination of 20 characters, the non-homoplastic ones being: semicircular to flabellate connectives (Characters 78 and 80); seeds with reticulate to foveolate testa (Character 100); and leaf epidermis possessing specialized cells with thickened walls carrying silica bodies (Character 104) (Figs 3, 4A, 5 clade F). This clade is recovered inside a polytomy, together with *Tradescantia* *s.s.* and the polyphyletic *Gibasis* (BS= 66; BI=5). The polytomy composed of *Tradescantia* *s.s.*+*Gibasis* grade+(*Tripogandra* *s.l.*+*Callisia*) is sister to *Elasis* *s.l.* (Fig. 3).

### *Tradescantia* *s.s.*

*Tradescantia* *s.s.* was recovered arranged in five well-supported clades, with the innermost clades herein called Core *Tradescantia* (Figs 3, 4A, 5 clade L), which possesses most of the genus morphological diversity and includes most of its species richness. The first lineage to diverge in *Tradescantia* *s.s.* represents T. sect. Austrotradescantia (*sensu* Pellegrini 2015). The section is recovered as monophyletic (BS= 94; BI= 5), being supported by: sepals elliptic to broadly elliptic (Character 55), all keeled (Character 56); filaments basally, densely bearded (Characters 66–69 and 67–70, homoplastic) with long moniliform hairs (Characters 68 and 71); style obconic at base (Character 89) and conic at apex (Character 90), stigma punctate (Character 91) with type D papillae (Character 92); seeds with costate testa (Character 100); chromosome count of *n*=10–numerous (Character 106), bimodal chromosomes (Character 108, homoplastic); and the presence of 1 C-glycosides (Character 111, homoplastic) (Figs 3, 4A & 5 clade I). The section is further divided into two fairly-supported clades (Figs 3 & 4A). The first one has medium support (BS= 66; BI= 3) and was named by Pellegrini (2015) as the *T. fluminensis* group, being characterized by: prostrate (Character 3, homoplastic), herbaceous stems (Character 4, homoplastic), chartaceous or membranous leaf-blades (Character 10, homoplastic), saccate cincinni bracts (Character 43, homoplastic), the presence of 6-hydroxy-luteine (Character 113, homoplastic), and by the absence of sulphated phenolic acids (Character 114, homoplastic). The majority-rule recovers the *T. fluminensis* group arranged in two morphological complexes (Fig. 4A), that can be interpreted as: (1) the *T. tenella* species complex (BS= 57), being characterized by its rugose seeds (Character 100, homoplastic), and hilum shorter than ½ the length of the seed (Character 102, homoplastic); (2) and as the *T. fluminensis* species complex, being characterized by plants with indefinite base (Character 2, homoplastic), and equal cincinni bracts (Character 40, homoplastic). The second clade has high statistical support (BS= 90; BI= 4) and was named by Pellegrini (2015, 2016) as the *T. crassula* group, being characterized by its: stems unbranched to branched only at base (Character 5, homoplastic), conduplicate and/or falcate leaf-blades (Character 11, homoplastic), inconspicuous secondary veins (Character 21, homoplastic), pistil longer than the stamens (Character 86, homoplastic), seeds cleft towards the embryotega (Character 96, homoplastic), and hilum longer than ½ the length of the seed (Character 102, homoplastic).

The second lineage in *Tradescantia* is highly supported and here named the *Campelia* clade (BS= 91; BI= 6), being composed by T. sect. Campelia, T. sect. Corinna, T. sect. Cymbispatha, T. sect. Rhoeo, and T. sect. Zebrina (Figs 3, 4A, 5 clade K). The clade is supported by: synflorescence with one or more coflorescences (Character 26), presence of peduncle bracts (Character 29), presence of supernumerary bracts (Character 37, homoplastic), spathaceous cincinni bracts (Character 39, homoplastic); flowers with pedicels geniculate at anthesis and pre-anthesis (Character 51), unequal sepals (Character 54), androecium with filaments from outer series shorter than the inner (Character 72, homoplastic), pollen white *in vivo* (Character 83, homoplastic), pistil longer than the stamens (Character 86, homoplastic), and semilateral embryotega (Character 98, homoplastic) (Figs 3, 4A). The monospecific T. sect. Rhoeo is recovered as an independent lineage at the base of the *Campelia* clade, sister to two clades. One of these clades represents a monophyletic T. sect. Cymbispatha (*sensu* Pellegrini et al. 2016), herein called the *T. commelinoides* group, being well-supported (BS= 76; BI= 5) and characterized by: herbaceous stems (Character 4, homoplastic); distichously-alternate leaves (Character 6, homoplastic), with membranous leaf-blades (Character 10, homoplastic), and acute apex (Character 20, homoplastic); equal (Character 40, homoplastic), basally fused (Character 42), not overlapping cincinni bracts (Character 44, homoplastic); keeled dorsal sepal (Character 56, homoplastic), chartaceous sepals (Character 57, homoplastic), rotund to rhomboid petals (Character 61, homoplastic), and pistil shorter than the stamens (Character 86, homoplastic) (Figs 3, 4A). Tradescantia sect. Cymbispatha is recovered as sister to a small, well-supported (BS= 79; BI= 4) clade, herein called the *T. zebrina* group, composed by T. sect. Campelia, T. sect. Corinna, and T. sect. Zebrina. Tradescantia sect. Campelia and T. sect. Corinna are monospecific, being recovered in the strict consensus in a polytomy with both sampled species of T. sect. Zebrina (Fig. 3), thus making the section paraphyletic. In the majority-rule (Fig. 4A), T. sect. Zebrina is also recovered as paraphyletic, due to the inclusion of *T. soconuscana* Matuda, the sole species of T. sect. Corinna. The *T. zebrina* group is supported by: leaf-blades abaxially velutine (Character 16, homoplastic); flat cincinni bracts (Character 40, homoplastic), with saccate base (Character 42, homoplastic); tubular flowers (Character 46, homoplastic), sepals basally to completely fused (Character 52), stigmatic papillae longer than 1μm (Character 92, homoplastic); and chromosomes with asymmetric complements (Character 107, homoplastic) (Figs 3, 4A). The sister relation between the *T. zebrina* and the *T. commelinoides* group is fairly well-supported (BS= 61; BI= 4), being sustained by: the presence of pubescence in the abaxial side of the leaves (Character 15, homoplastic), leaf-blades cuneate at base (Character 18, homoplastic); sessile to subsessile flowers (Character 49, homoplastic); connectives ranging from cordate to sagittate to linearly-tapered (Characters 77 and 79, homoplastic), rounded anther sacs (Characters 80 and 81, homoplastic); seeds with hilum equal to ½ the length of the seed (Character 100, homoplastic); and chromosome count *n*= 7 and 8, *n*= 5, 6, 10 and 11, due to Robertosnian Changes (Character 104) (Figs 3, 4A). The *Campelia* clade is recovered as sister to Core *Tradescantia* with high statistical support (BS= 92; BI= 8), and sustained by: spirally-alternate leaves (Character 6, homoplastic), leaf-blades with truncate to amplexicaulous (Character 18, homoplastic), symmetric base (Character 19, homoplastic), acuminate apex (Character 20, homoplastic); overlapping cincinni bracts (Character 43, homoplastic), conspicuous bracteoles sometimes completely involving the cincinnus (Character 44, homoplastic); membranous sepals (Character 56), stigmatic papillae equal or shorter than 1μm (Character 92, homoplastic); conspicuous embryotega (Character 97); equal or larger than 10µm (Character 105), symmetric chromosomes (Character 107) (Figs 3, 4A, 5 clade J).

### Core *Tradescantia*

As aforementioned, Core *Tradescantia* consists of three smaller clades (herein called the *Mandonia*, *Setcreasea*, and *Tradescantia* clades), restricted to drier environments (i.e. Seasonally Dry Tropical and Subtropical Forests) in the American continent. Core *Tradescantia* is well-supported (BS= 88; BI= 4; 5 clade L), and defined by: tuberous roots (Character 1, homoplastic); stems unbranched to branched only at base (Character 5, homoplastic); conduplicate leaf-blades (Character 11, homoplastic); petals ranging from lilac to purple or pink (Character 63, homoplastic), connectives quadrangular to rectangular to slightly curved (Characters 77 and 79), anther sacs C-shaped (Characters 80 and 81), and pistil the same length as the stamens (Character 85, homoplastic). In the strict consensus, all the three clades are recovered inside a polytomy (Fig. 3), while in the majority-rule, the *Tradescantia* clade is the first to diverge, being sister to the *Mandonia* and *Setcreasea* clades (Figs 4A, 5 clade N). The *Tradescantia* clade is well-supported (BS= 96; BI= 3), and composed by most species of T. sect. Tradescantia (i.e. series *Virginianae* and *Tuberosae*), thus including the type-species of the genus (i.e. *T. virginiana* L.). Nonetheless, T. sect. Tradescantia *sensu* Hunt (1980) is still recovered as paraphyletic, due to series *Sillamontanae* and *Orchidophyllae* being nested within the *Setcreasea* clade (Figs 3, 4A). The *Tradescantia* clade is supported by: linear leaf-blades (Character 9, homoplastic); pedicels apically non-gibbous (Character 49, homoplastic), filaments densely bearded with moniliform hairs (Characters 65 and 68, homoplastic), and stigmatic papillae restricted to the margins of the stigma (Characters 93) (Figs 3, 4A, 5 clade M). The sister relation between the *Mandonia* clade and *Setcreasea* clade has low statistical support (BS= 65; BI= 1), as evidenced by the polytomy recovered in the strict consensus tree. However, it is morphologically supported by: pubescent ovary with eglandular hairs (Character 84); hilum shorter than ½ the length of the seed (Character 100, homoplastic); production of hydroxy-luteolin (Character 108, homoplastic), the absence of 6-hydroxy-lutein (Character 111, homoplastic), and the absence of sulphated phenolic acids (Character 112, homoplastic) (Figs 3, 4A, 5 clade N). The *Mandonia* clade is a well-supported clade (BS= 91; BI= 4), composed by T. sect. Mandonia and T. sect. Parasetcreasea. It is supported by: leaf-blades abaxially pubescent (Character 15, homoplastic); inflorescences mainly axillary, producing a raceme-like synflorescence (Character 24, homoplastic), sessile main florescences (Character 28, homoplastic), the presence of supernumerary bracts (Character 36, homoplastic), reduced cincinni bracts (Character 37, reversion), cincinni bracts not overlapping (Character 43, homoplastic); chartaceous sepals (Character 56, homoplastic), filaments apically spirally-coiled at post-anthesis (Character 73), style ½ time longer than the stamens (Character 85), and style spirally-coiled at post-anthesis (Character 87) (Figs 3, 4A, 5 clade O). Finally, the *Setcreasea* clade is statistically well-supported (BS=92; BI=4), despite being defined exclusively by homoplastic characters. It is characterized by the combination of: leaf-blades with cuneate base (Character 18), acute apex (Character 20), inconspicuous secondary veins (Character 21); saccate cincinni bracts (Character 42); tubular flowers (Character 46), pedicel the same length as the floral buds (Character 49), hyaline sepals (Character 58), fused petals (Character 59), clawed petals (Character 61), epipetalous stamens (Character 74); and chromosomes medium-sided (i.e. bigger than 5µm and smaller than 10µm; Character 105) (Figs 3, 4A, 5 clade P). The *Setcreasea* clade is composed by T. sect. Separotheca, T. sect. Setcreasea, and the remaining species of T. sect. Tradescantia (i.e. series *Sillamontanae* and *Orchidophyllae*).

## Discussion

### *Tradescantia* phylogeny and congruence between different datasets

The present study features the most extensive sampling of *Tradescantia* and its relatives, in a phylogenetic analysis (almost 50% of the species currently accepted in the genus), and most of the morphological diversity in subtribe Tradescantiinae (*sensu* Faden and Hunt 1991). It is also the first phylogenetic study to sample all sections and series proposed by Hunt (1975, 1980, 1986b), including all type species for each infrageneric rank. This study is also the first morphologically based phylogeny for *Tradescantia*, and the first in the family to include macromorphological, anatomical, palynological, cytological, and phytochemical characters. A high degree of homoplasy is observable in the present dataset, based on the CI, RI, and RC indexes, being congruent with the scenario expected for Commelinaceae (Evans and Faden 1998; Evans et al. 2000). Nonetheless, contrary to results for the whole family of Evans and Faden (1998) and Evans et al. (2003) in which their morphologically derived topology was highly incongruent with the molecular dataset (Evans and Faden 1998; Evans et al. 2003), the herein presented relationships inside *Tradescantia*, are congruent with the ones previously recovered for the genus, based on molecular data (Burns et al. 2011; Hertweck and Pires 2014; Figs 4A,B, 5). The Bremer Index, which is used for the first time in phylogenetic analysis in Commelinales, also gives strong statistical support for most clades, despite the homoplasy in the present dataset. The incongruence between the morphological and molecular datasets observed by Evans and Faden (1998) and Evans et al. (2000, 2003), might be a reflection of the differentiated coding of some key morphological characters. This is mainly due to the dramatically different sampling between the present study (i.e. subtribal and generic level) and the studies by Evans and Faden (1998) and Evans et al. (2000, 2003; i.e. family and infrafamily level). Characters related to inflorescence architecture and androecium morphology, were key in resolving the backbone of the present analysis. Furthermore, micromorphological, anatomical, palynological, cytological, and phytochemical characters were also essential in giving support to the backbone of the herein presented topology. From a wider perspective and despite the high degree of homoplasy, it is not unexpected for well-coded morphological characters to be highly congruent to molecular datasets. In Commelinales, Haemodoraceae and Pontederiaceae have yielded similar results regarding the congruence between different datasets. In Haemodoraceae, the morphological phylogeny by Simpson (1990) is widely corroborated by the molecular phylogeny by Hopper et al. (2009), with recent anatomical data Aerne-Hains and Simpson (2017) further increasing the congruence between morphology and molecular data. In Pontederiaceae the congruence is even clearer, where all phylogenies for the family published so far (i.e. Eckenwalder and Barrett 1986; Graham and Barrett 1995; Kohn et al. 1996; Barret and Graham 1997; Graham et al. 1998; Ness et al. 2011) recover the same evolutionary history, regardless of the dataset (Pellegrini 2017).

### Systematics and generic limits in Tradescantiinae

As aforementioned, the results of the present study are highly congruent with previous phylogenetic studies. Nonetheless, they still differ significantly from the previous ones, regarding generic limits and relationships. Similar to Hertweck and Pires (2014), the herein presented analysis recovers *Tradescantia* as paraphyletic in its current circumscription. In Hertweck and Pires (2014), the paraphyly of *Tradescantia* is caused by two species of *Gibasis*. In the present analyses, the paraphyly of *Tradescantia* is due to the position of *T. guatemalensis* as sister to *E. hirsuta*. This is the first time *T. guatemalensis* is sampled in any phylogenetic study, but based on the protologue of T. sect. Coholomia (Hunt 1980) describing the cincinni as arranged side by side, it was clear that this species was placed in the wrong genus. *Elasis*, as described by Hunt (1978), is characterized by possessing free, elongated, non-geniculate and fasciculate cincinni (which can also be interpreted as several cincinni emerging side by side from the same node), six equal stamens, inconspicuous connectives, and truncate stigma. This description matches perfectly with the one of *T. guatemalensis*, with both species being differentiated solely on pubescence, inflorescence position, and flower color. Thus, it is clear that in order to maintain a monophyletic *Tradescantia* and a coherent *Elasis*, *T. guatemalensis* needs to be transferred to the later. *Elasis* *s.l.* can be differentiated from *Tradescantia* *s.s.* by the following characters: 1–several, shortly-pedunculate, free, elongate cincinni (Characters 30, 31, 32, 34, 35), bracteose cincinni bracts (Character 38), anther connectives not expanded (Characters 77 & 79), silica crystals in specialized cells with thickened walls (Character 103 & 104), bundle sheath in the mesophyll with longitudinal sclerenchymatic extensions (Character 105) and chromosome number *n*= 4–5 (Character 106).

The present analysis was also unable to recover a monophyletic *Gibasis*. Nonetheless, in the present analyses *Gibasis* is not partially nested within *Tradescantia*, as recovered by Hertweck and Pires (2014), being actually distantly related to *Tradescantia*. In fact, the herein presented results suggest a closer relationship between *Tradescantia* and the *Callisia*+*Tripogandra* clade, based on inflorescence architecture. *Tradescantia* and *Gibasis* are morphologically, anatomically, cytologically, and phytochemically very distinct, and should be kept as distinct genera (Fig. 5). I believe that the present analysis was unable to recover a monophyletic *Gibasis* due to the limited sampling of this morphologically diverse genus in the present study. The present sampling of *Gibasis* had solely the intention of testing its relationship to *Tradescantia*, and not its monophyly. According to the simulations of Hillis (1996), the greater sampling for a certain group, the higher its statistical support and phylogenetic resolution. Furthermore, I was unable to sample the type species of *Gibasis* (i.e. *G. pulchella*) in the present study. Thus, I believe that further analysis focused on *Gibasis* and its systematics, might yield different results. Aside from recovering the genus as monophyletic, it would possibly recover the geniculate cincinni as a synapomorphy for *Gibasis*, as hypothesized by Hunt (1986a).

**Figure 6.**
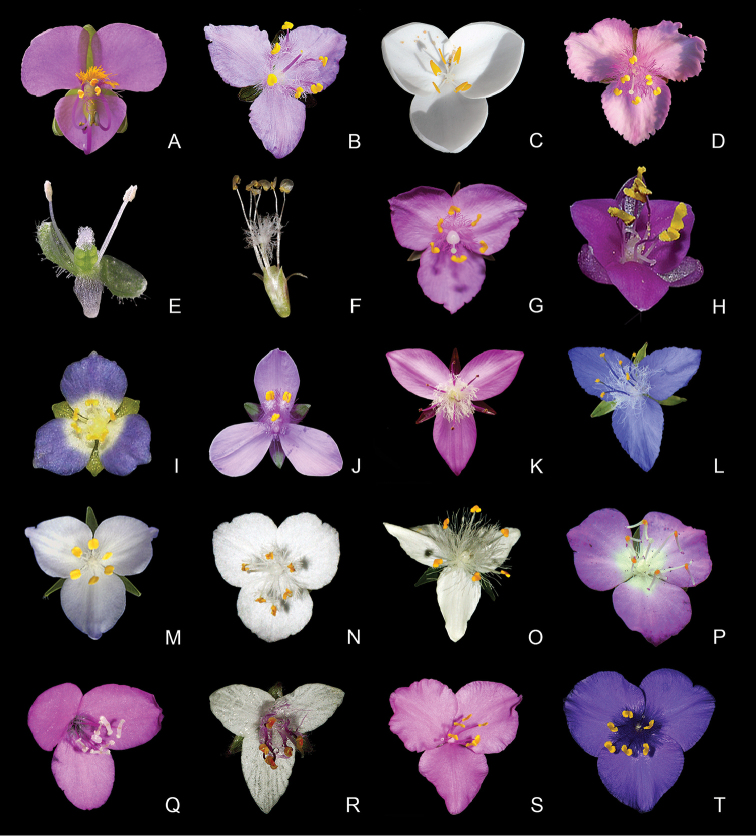
Floral morphology of subtribe Tradescantiinae *s.l.* **A** *Tinantia* Scheidw **B** *Thyrsanthemum* Pichon **C** *Weldenia* Schult.f. **D–I** *Callisia* Loefl. *s.l.*: **D** *Cuthbertia* Small **E** *Aploleia* Raf. (i.e. C. sect. Leptocallisia *s.s.*) **F** *Callisia* *s.s.* (i.e. C. sect. Callisia) **G** Callisia sect. Brachyphylla D.R.Hunt **H** *Hadrodemas* H.E.Moore (i.e. C. sect. Hadrodemas) **I–J** *Tripogandra* Raf. *s.l.*: **I** Callisia sect. Leptocallisia *pro parte* **J** *Tripogandra* *s.s.* **K–L** *Elasis* D.R.Hunt: **K** *E. hirsuta* (Kunth) D.R.Hunt **L** *E. guatemalensis* C.B.Clarke *ex* Donn.Sm.) M.Pell **M** *Matudanthus* D.R.Hunt **N** *Gibasis* Raf. **O–T** *Tradescantia* L. emend. M.Pell.: **O** T. subg. Austrotradescantia **P–Q** T. subg. Campelia **R** T. subg. Mandonia **S** T. subg. Setcreasea **T** T. subg. Tradescantia. **A** by E. Barbier, **B, L, P** by P. Acevedo-Rodriguez, **C** by S. Cross, **D** by D. Rankin, **E** by J. Amith, **F, J, N–O, Q–T** by M.O.O. Pellegrini, **G** by S. Eduardo, **H** by C. Willemsen, **I** by B.E. Hammel, **K** by A. Kay, and **M** by A. Garcia Mendoza.

*Callisia* is a historically challenging group in Commelinaceae, especially regarding its taxonomy (Hunt 1986c). Once again, the genus is recovered as non-monophyletic, with species being recovered together with *Tripogandra* *s.s.* and with the different sections proposed by Hunt (1986c) being recovered as distinct lineages. The present topology is highly congruent with the molecular-based hypothesis presented by Bergamo (2003). In the *Callisia* *s.s.* lineage recovered in the present study (i.e. Fig. 5 clade F), two morphological groups are clearly distinguishable. The first is represented in the present dataset exclusively by *C. monandra* (Sw.) Schult. & Schult.f., and can be characterized by its pedunculate double-cincinni, long-pedicellate flowers, glandular-pubescent pedicels and sepals, androecium reduced to 1–3, antesepalous stamens, anthers with elongate anther sacs, inconspicuous connectives, and sessile stigmas, being equivalent to C. sect. Leptocallisia *s.s.* [i.e. *Aploleia* Raf. *sensu* Moore Jr. 1961, with the addition of *C. cordifolia* (Sw.) E.S. Anderson & Woodson]. The remaining species sampled in the present study represent C. sect. Callisia, due to the inclusion of the type species [i.e. *C. repens* (Jacq.) L.]. This lineage is characterized by its sessile double-cincinni, short-pedicellate flowers, sepals glabrous or with eglandular hairs, androecium with (3–)6 stamens, when reduced to 3 stamens than antepetalous, round anther sacs, expanded flabellate or sagittate connectives, and elongated styles. Based on the molecular-based results by Bergamo (2003), combined with the herein presented morphological results, I suggest that both groups should probably be recognized as distinct genera in the near future (Pellegrini in prep.). Aside from that, other lineages of *Callisia* *s.l.* recovered by Bergamo (2003) and Hertweck and Pires (2014) possess specific morphologies (Fig. 6D, G), but were not sampled in the present study. In the same way as *Callisia* *s.s.* and *Aploleia*, these lineages also need to be recognized at the generic level, in order to maintain a morphologically cohesive and monophyletic *Callisia* (Pellegrini in prep.).

As aforementioned, *C. warszewicziana* was excluded from this analysis due to the great noise this extremely autapomorphic species created. The exclusion of *C. warszewicziana* caused no changes in the backbone of the analysis, but considerably increased the statistical support for all branches. As recovered by Bergamo (2003) and Hertweck and Pires (2014), *C. warszewicziana* is nested within the *Callisia*/*Tripogandra* generic complex, but morphologically deviant from all remaining species (Fig. 6F). Ongoing phylogenetic studies in the group seem to reveal the need to reestablish *Hadrodemas* H.E.Moore and other satellite genera lumped in *Callisia* *s.l.* by Hunt (1986c), in order to retain morphologically cohesive and monophyletic genera in subtribe Tradescantiinae (Pellegrini in prep.). *Tripogandra* as circumscribed by Handlos (1975) is also recovered as paraphyletic. This result is not surprising, since Bergamo (2003) had already recovered part of C. sect. Leptocallisia [i.e. *C. gracilis* (Kunth) D.R.Hunt] nested within *Tripogandra*. *Callisia gracilis* and *C. filiformis* both share the typical *Tripogandra*-type inflorescence pattern, with the cincinni bracts reduced to only a membranous crest (i.e. vestigial), the 60°-degree torsion in floral display, the outer whorl of stamens shorter than the inner whorl, and basic chromosome count of *n*= 8 (Fig. 5 clade G; pers. observ.). Aside from these species, *C. ciliata* Kunth is also morphologically similar, being a putative candidate for being placed in *Tripogandra* *s.l.* Pollen morphology should play an important role into solving this problem, since *Tripogandra* is known to be the only genus in the family with granular-verrucose tectum (Poole and Hunt 1980). I believe that with an improved sampling of *Callisia* and *Tripogandra*, it would be possible to shed some light on this intricate group and potentially solve this taxonomic impasse. Nonetheless, I also believe it would be precocious to implement taxonomic changes in the *Callisia*/*Tripogandra* complex in the present study, without further studies focusing in the group.

Subtribe Thyrsantheminae has been consistently recovered as polyphyletic by all morphological and molecular phylogenies so far (Evans et al. 2000, 2003; Wade et al. 2006; Burns et al. 2011; Zuiderveen et al. 2011; Hertweck and Pires 2014; Pellegrini et al. unpublished data). Additionally, the subtribe completely lacks any kind of micro- or macromorphological synapomorphies. This should not be unexpected, since Faden and Hunt (1991) when formally proposing the subtribe, comment on the marked heterogeneity of the group. The *Tradescantia* alliance, as proposed by Hertweck and Pires (2014), represents the exclusively Neotropical crown group in tribe Tradescantieae, being composed by all genera of the non-monophyletic subtribes Thyrsantheminae and Tradescantiinae *s.s.* This clade can be uniquely defined by the presence of pollen grains with rugose to rugose-insulate tectum as its synapomorphy, and originating the specialized tectum ornamentation of genera such as *Callisia*, *Tinantia* and *Tripogandra* (Poole and Hunt 1980; pers. observ.). As originally envisioned by Hunt (1983), subtribe Thyrsantheminae was characterized as possessing: free, stipitate and non-geniculate cincinni, sometimes reduced to 1–3-flowered and sessile cincinni; actinomorphic flowers; and six, fertile and equal stamens with expanded connectives. This diagnosis only needed to be slightly expanded by Faden and Hunt (1991) in order to include *Weldenia*, but due to the inclusion of *Tinantia* this diagnosis was expanded to also include zygomorphic flowers, and unequal stamens with inconspicuous connectives (Faden and Hunt 1991; Hunt 2015a,b,c). Even in its original circumscription (i.e. Hunt 1983), subtribe Thyrsantheminae still represented a polyphyletic and heterogeneous assemblage, with different genera possessing either alternate or fasciculate cincinni, inconspicuous or expanded connectives (the connectives of *Elasis* and *Matudanthus* D.R.Hunt are not expanded, and the connectives of *Thyrsanthemum* are very seldom so), and dorsal or semilateral embryotega (pers. observ.). Furthermore, based on the diagnosis provided by Faden and Hunt (1991), it is unclear why *Gibasis* was not included in this subtribe, instead of being included in Tradescantiinae. *Gibasis* lacks the diagnostic double-cincinni of Tradescantiinae *sensu* Faden and Hunt (1991), matching perfectly the diagnosis of Thyrsantheminae. Based on morphological and molecular data (Evans et al. 2000, 2003; Wade et al. 2006; Burns et al. 2011; Zuiderveen et al. 2011; Hertweck and Pires 2014; Pellegrini et al. unpublished data; this study), *Gibasis* is clearly more closely related to *Elasis* than it is to *Tradescantia*, as shown by Hertweck and Pires (2014). Furthermore, *Matudanthus* is unambiguously closely related to *Elasis*, being differentiated exclusively by the presence of tuberous roots, sessile cincinni, and tannin cells in the petals (Hunt 1978; pers. observ.). When finally sampled in a phylogenetic study, it is expected for *Matudanthus* to emerge as part of the *Gibasis*+*Elasis* clade, due to its inflorescence architecture, floral morphology, seed morphology, pollen features and anatomical pattern. Another monospecific genus not sampled in any phylogenetic study so far, *Gibasoides* D.R.Hunt, is strikingly similar to *Thyrsanthemum*. Both genera can only be differentiated based on the development of the main axis of the inflorescence, which produces a perfect thyrse with elongate main axis and alternate cincinni in *Thyrsanthemum*, and an umbellate-thyrse with inconspicuous main axis and verticillate cincinni in *Gibasoides* (Hunt 1978). Whether it will be recovered as sister to *Thyrsanthemum* or nested within the latter when included in a phylogenetic study, remains to be seen. For the time being, it is better to recognize them as distinctive genera. Finally, the last poorly-known genus in the *Tradescantia* alliance, *Sauvallea* C.Wright *ex* Hassk., received recent attention by Hunt and Arroyo-Leuenberger (2015). The authors clarify several morphological features for the genus, but due to the probable extinction of the its sole species, *S. blainii* C.Wright *ex* Hassk., our knowledge on this taxon is still limited. The genus was treated as *incertae sedis* by Faden and Hunt (1991) and Faden (1998), being a putative member of either subtribe Tradescantiinae or Thyrsantheminae. Its inflorescence morphology is unique in the *Tradescantia* alliance due to its spathaceous basal bract, being reminiscent of *Cyanotis* D.Don *s.l.* (including *Belosynapsis* Hassk.; Hunt and Arroyo-Leuenberger 2015). However, *Cyanotis* *s.l.* possesses an exclusively Palaeotropical distribution, inflated filaments and styles, generally basally poricidal anthers, and apical embryotega. Thus, it is unlikely that *Sauvallea* proves to be a member of subtribe Cyanotinae, if it is ever recollected and/or sampled in a phylogenetic study (pers. observ.).

### The relevance of inflorescence architecture in the systematics of Tradescantiinae

Inflorescence morphology has been widely used in the taxonomy of Commelinaceae, throughout the years (e.g. Clarke 1881; Brückner 1930; Pichon 1946; Hunt 1975, 1980, 1986a, 1986b, 1986c; Panigo et al. 2011) for the delimitation of different taxonomical ranks. The herein presented analysis corroborates that different inflorescence patterns are indeed recovered as synapomorphies for different lineages. *Tinantia* can be easily differentiated from ingroup genera by its leaf-like basal bract [that becomes somewhat spathaceous and conduplicate in species like *T. anomala* (Torr.) C.B.Clarke], pedunculate inflorescence with verticillate, free, and non-geniculate cincinni, and conspicuous and persistent bracteoles (Figs 3, 4A, 5 clade A). Based on the molecular results published so far (Bergamo 2003; Evans et al. 2003; Wade et al. 2006; Burns et al. 2011; Hertweck and Pires 2014), tubular and hyaline basal bracts seem to have evolved only once in Commelinaceae, being a putative synapomorphy for the clade composed by *Callisia* *s.l.*, *Elasis*, *Gibasis*, *Tradescantia*, and *Tripogandra* *s.l.* (i.e. clade B, Fig. 5). Despite the limited sampling of the *Tradescantia* alliance in the present study, the recovered topology also supports this hypothesis (Figs 3–5). All remaining genera in the family have either leaf-like, bracteose, or spathaceous basal bracts (pers. observ.). In the clade composed by *Thyrsanthemum*+*Weldenia* (and most likely also including *Gibasoides*), the inflorescences are characterized by their alternate cincinni, subtended by linear bracts, and greatly reduced, generally absent or deciduous bracteoles (Hunt 1978, 2015b, c). If confirmed to be part of this clade, the inflorescence architecture of *Gibasoides* could be easily explained by the suppression of the main axis of the inflorescence, causing the alternate cincinni to acquire as pseudo-verticillate arrangement. This feature could easily derived from a inflorescence very similar to the one of *Thyrsanthemum goldianum* D.R.Hunt, which is the only species in the genus with long-stipitate cincinni, equivalent to the ones of *Gibasoides laxiflora* (C.B.Clarke) D.R.Hunt (Hunt 1978, 2015c; pers. observ.).

The double-cincinni fused back to back, seems to have evolved only once in Commelinaceae, recovered in the present study as a synapomorphy for the clade composed by *Callisia* *s.l.*, *Tradescantia*, and *Tripogandra* *s.l.* (i.e. clade D, Fig. 5). Nonetheless, molecular based phylogenetic studies (i.e. Bergamo 2003; Evans et al. 2003; Wade et al. 2006; Burns et al. 2011; Zuiderveen et al. 2011; Hertweck and Pires 2014; Pellegrini et al. in prep.) recover the double-cincinni as a synapomorphy for subtribe Tradescantiinae (*sensu* Faden and Hunt 1991) plus *Elasis* (i.e. clade B, Fig. 5). In this scenario, the reversion from the double-cincinni to verticillate or fasciculate cincinni is synapomorphic for the clade formed by *Gibasis*+*Elasis* (and most likely also including *Matudanthus*). Despite being recovered in the present study as polyphyletic, *Gibasis* can be recognized by its main florescences with free, verticillate and geniculate cincinni (Hunt 1986a; Fig. 5). The double-cincinni is very conserved in this clade, however in *C. warszewicziana* the cincinni are commonly only basally fused, and can range from 1–2(–3). Some variation is also observed in *Tradescantia* *s.s.*, with some species sometimes producing abnormal inflorescences with alternate looking cincinni (e.g. *T. cymbispatha*, *T. fluminensis*, and *T. zanonia*), others commonly producing axillary inflorescences with a solitary cincinnus (e.g. *T. crassula* and all species in the *Mandonia* clade), and others producing inflorescences with 2–3(–5) cincinni, fused back to back (i.e. *T. valida*) (pers. observ.). As aforementioned, *Tripogandra* *s.l.* can be easily defined by the extreme reduction of the cincinni bracts, coupled with its double-cincinni fused back to back, and the 60° torsion in floral display. The polyphyletic *Callisia* *s.l.* also possesses several inflorescence characters that give morphological support to the different lineages recovered by molecular datasets (Bergamo 2003; Evans et al. 2003; Wade et al. 2006; Burns et al. 2011; Zuiderveen et al. 2011; Hertweck and Pires 2014; Pellegrini et al., in prep.), and on a smaller scale the results herein presented. *Callisia* *s.s.* (i.e. C. sect. Callisia *sensu* Hunt 1986c) could be easily characterized by its synflorescences with mainly axillary main florescences, sessile main florescences, cincinni bracts similar to the bracteoles, double-cincinni fused back to back, tubular flowers, white to hyaline petals, glabrous filaments, flabellate connectives, and penicilliform stigmas.

*Sauvallea blainii* must once again be considered a taxon of uncertain phylogenetic affinity regarding inflorescence morphology. As explained by Hunt and Arroyo-Leuenberger (2015), due to the paucity and poor preservation of available specimens, our knowledge on the genus will remain limited until it is, if ever, recollected. Nonetheless, Hunt and Arroyo-Leuenberger (2015) were able to describe the main florescence as being greatly reduced, apparently to a solitary cincinnus, with conspicuous and persistent bracteoles (which can be observed protruding from the spathaceous basal bract), and with each cincinnus producing most probably only one flower. Based on this description, *Sauvallea* could be more closely related to *Tinantia*, with which it shares the frondose (i.e. spathaceous or leaf-like) basal bract, and expanded and persistent bracteoles. The number of cincinni in *Tinantia* is variable within and between species, with several species known to produce solitary cincinni (Faden 1998). Furthermore, *Sauvallea* and *Tinantia* also share an annual life cycle, subequal to unequal petals, and six stamens bearded with moniliform hairs (pers. observ.). Alternatively, it is also possible for *Sauvallea* to represent a further reduction of the inflorescence architecture exhibited by *Callisia* *s.l.* This scenario is supported by the description of Hasskarl (1870), in which he describes the androecium as similar to *Tradescantia* due to its six equal stamens, barbate with moniliform hairs, expanded connectives, and gynoecium similar to *Callisia* due to its bilocular and pilose ovary and elongate style. This description brings *S. blainii* considerably closer to *C. cordifolia* (i.e. *Aploleia* *sensu* Moore Jr. 1961, or C. sect. Leptocallisia *sensu* Hunt 1986c) due to floral morphology, which would explain the common misidentifications between the two species in Cuba (Hunt and Arroyo-Leuenberger 2015). Nonetheless, this relationship would require major changes in the inflorescence architecture (e.g. shortening of the peduncle, reduction of the double-cincinni to a solitary cincinnus accompanied by the expansion of the cincinni bract, and shortening of the pedicels). Both hypotheses remain to be tested once both genera are sampled in a comprehensive phylogenetic study.

### Evolution of petal morphology in *Tradescantia* *s.s.*

When Hunt (1975) reduced *Setcreasea* to a section of *Tradescantia*, the author commented on the strong emphasis given by previous authors to sympetaly and epipetaly, questioning its systematic significance. As aforementioned, these characters can be found in other genera of Commelinaceae, as well as in three lineages of *Tradescantia* (i.e. the *Campelia*, *Setcreasea*, and *Mandonia* clades). Nonetheless, these two characters played a significant part in Hunt’s sectional treatment of *Tradescantia*. Furthermore, sympetaly has also been regarded by other Commelinaceae specialists as a key morphological character in the evolution and systematics of the group. It has been used as the defining character of tribes, subtribes, genera, and sections (e.g. Clarke 1881; Brückner 1930; Pichon 1946; Hunt 1975, 1980, 1986a, 1986b, 1986c). Despite his own critical view on sympetaly, Hunt (1975, 1980, 1986b) also gave great weight to this character, making it decisive in differentiating some of the sections in *Tradescantia*, such as: (1) *T.* sect. Mandonia vs. T. sect. Parasetcreasea; and (2) T. sect. Campelia + *T.* sect. Corinna vs. T. sect. Zebrina. Nonetheless, systematic studies have shown that this character probably evolved several times in the family (Bergamo 2003; Evans et al. 2003; Wade et al. 2006; Burns et al. 2011; Zuiderveen et al. 2011; Hertweck and Pires 2014). Furthermore, my observations have revealed that sympetaly does not seem to be constant in the same evolutionary lineage, suffering several reversions along the way (Figs 3, 5). Finally, *T. pallida* produces flowers either with conate or free petals, sometimes in the same individual, which gives further support to my interpretation.

Epipetaly is by far one of the least common and less studied characters in Commelinaceae. It is found exclusively in some species of *Tradescantia* and *Weldenia*. In *Tradescantia*, epipetaly is only found in 12 species from six sections (i.e. corresponding to three clades in our analysis): *T. andrieuxii* C.B.Clarke, *T. brevifolia* (Torr.) Rose, *T. buckleyi* (I.M.Johnst.) D.R.Hunt, *T. hirta* D.R.Hunt, *T. leiandra* Torr., *T. mirandae* Matuda, *T. orchidophylla* Rose & Hemsl., *T. pallida* (Rose) D.R.Hunt, *T. pygmaea* D.R.Hunt, *T. rozynskii* Matuda, *T. sillamontana* Matuda, *T. schippii* D.R.Hunt, and *T. soconuscana* Matuda. In most species, the stamens are fused throughout or most of the claws length. However, in the species that do not possess clawed petals (i.e. *T. mirandae*, *T. orchidophylla*, *T. rozynskii*, and *T. sillamontana*), the stamens are clearly basally fused to the petals and recorded by the first time in the present study. According the herein presented results, epipetaly does not seem to be directly connected either with sympetaly or clawed petals, but might actually represent an independent character.

Clawed petals are found in several genera from tribe Commelineae and Tradescantieae. In tribe Commelineae, clawed petals are restricted to genera from the *Commelina* clade (i.e. *Aneilema* R.Br., *Commelina* L., *Dictyospermum* Wight, *Pollia* Thunb., *Rhopalephora* Hassk., and *Tapheocarpa* Conran), that together with the presence of hook-hairs are putative synapomorphies for the group (Evans et al. 2003). In all these genera, the petals are invariably free from each other. In tribe Tradescantieae clawed petals are uncommon, found in *Coleotrype* C.B.Clarke, *Cyanotis* *s.l.*, some species of *Tradescantia*, and *Weldenia*. These genera are in general distantly related, suggesting that this character evolved several times and has no obvious phylogenetic pattern. In *Tradescantia*, clawed petals have been previously observed in: *T. andrieuxii*, *T. brevifolia*, *T. buckleyi*, *T. hirta*, *T. huehueteca* (Standl. & Steyerm.) D.R.Hunt, *T. leiandra*, *T. pallida*, *T. pygmaea*, *T. schippii*, *T. soconuscana*, and *T. zebrina* Heynh. *ex* Bosse. Differently from the pattern observed in tribe Commelineae, in *Tradescantia* many, but not all, species with clawed petals also present connate petals.

Tubular flowers are generally associated by most taxonomists with sympetaly by traditional taxonomy. However, as indicated by Harris and Harris (2001), the shape of the perianth has no relation with the connation of the perianth segments. Thus, in the present study, I have considered flowers of species such as *T. zanonia* (L.) Sw. as possessing shallow, wide and funnelform to cupuliform tubes, instead of being truly flat, as in *T. fluminensis*. Flat flowers seem to be ancestral state in Commelinaceae, with most genera in the four major lineages of the family possessing primarily flat flowers. Non-flat flowers are found in *Callisia*, *Coleotrype*, *Cyanotis* *s.l.*, some species of *Tradescantia*, and *Weldenia*, being far more common than most of the characters described so far. Sympetaly and epipetaly have evolved at least three times in *Tradescantia*, and seem to be at least partially dependent to each other, as previously hypothesized by Rohweder (1956) and Hunt (1975). Still, tubular flowers are not necessarily correlated to the aforementioned characters. In the present topology it is possible to observe species that possess clearly tubular flowers, but no sign of sympetaly, clawed petals, or epipetaly (e.g. *C. fragrans* (Lindl.) Woodson, *C. gentlei* Matuda, and *C. repens*). There are also cases of species that possess tubular flowers, clawed petals, epipetaly, but free petals (e.g. *T. soconuscana*). Finally, there are species that possess tubular flowers and sessile and free petals, but present filaments basally conate to the petals (e.g. *T. mirandae*, *T. orchidophylla*, *T. rozynskii*, and *T. sillamontana*). These results support my decision to code each of these four characters as independent, since the same result was recovered when these characters were coded as a single character. Alternatively, the statistical support for some branches increased greatly when these characters were coded independently.

### Evolution of androecium morphology in Commelinaceae and its systematic relevance

Androecium morphology has historically been the most prominent character in the taxonomy and classification of Commelinaceae (Clarke 1881; Bentham and Hooker 1883; Brückner 1930; Woodson Jr. 1942; Pichon 1946; Brenan 1961; Faden and Hunt 1991; Faden 1998). Indeed, a great deal of variation is observed in the family as a whole, with some patterns characteristic or synapomorphic to several taxonomic ranks (Brückner 1930; Handlos 1975; Hunt 1983; Faden 1991, 1998; Faden and Hunt 1991; Pellegrini et al. 2016; Pellegrini and Faden 2017). The androecium morphology of Commelinaceae is known to vary regarding the: (1) symmetry of the androecium as a whole; (2) number of stamens; (3) fertility of the stamens; (4) similarity between the inner and outer whorl of stamens; (5) similarity within each whorl of stamens; (6) connation with the corolla; (7) filament connation; (8) position, curvature and torsion of the filaments; (9) pubescence of the filaments; (10) insertion of the anthers; (11) morphology of the connectives; (12) morphology of the anther sacs; (13) relative position of the anther sacs; (15) dehiscence of the anther sacs; and (16) fertility of the pollen grains (Faden 1998; Evans et al. 2000, 2003; pers. observ.). In Tradescantiinae, this plasticity is easily observable in most genera (Figs 5, 6). Nonetheless, different androecium features have been shown to be highly homoplastic by different phylogenetic studies (Evans et al. 2000, 2003; Wade et al. 2006; Burns et al. 2011; Zuiderveen et al. 2011; Hertweck and Pires 2014). This is not surprising, considering that all Commelinaceae lack nectaries of all types, and pollen is the only floral resource available for pollinators (Faden 1992, 2000). Thus, it should be expected for androecium morphology to be strongly connected to key shifts in pollination syndromes in different lineages of the family (Faden 1992, 2000; Pellegrini and Faden 2017).

On the other hand, when coupled with different macro and micromorphological characters, androecium morphology can be successfully used to circumscribe monophyletic groups in the family (e.g. Pellegrini et al. 2016; Pellegrini and Faden 2017). For instance, subfamily Cartonematoideae was characterized based on the absence of several morphological features present in subfamily Commelinoideae, such as raphide-canals and glandular microhairs (Faden and Hunt 1991). Nonetheless, *Cartonema* R.Br. and *Triceratella* Brenan can be uniquely characterized by the their cormose habit, thyrsi composed of several one-flowered cincinni (i.e. a pseudoraceme), enantiostylous flowers, persistent sepals completely enclosing and/or surpassing the capsules, petals generally yellow and with tannin cells, six fertile stamens with inconspicuous connectives, poricidal and connivent anthers, parallel anther sacs, bowl-shaped seeds with prominent embryotega, and the loss of the micropylar collar (Barker et al. 2001; Panigo et al. 2011; pers. observ.). These androecium characters are not exclusive to Cartonematoideae, but combined with the aforementioned morphological characters, uniquely circumscribe the subfamily. Correspondingly, as recovered in the present phylogeny, androecium characters combined with different morphological characters, successfully circumscribe several lineages (Figs 3–6). For instance, *Tradescantia* *s.s.* can be easily differentiated from *Gibasis* and *Elasis* based on inflorescence morphology and anatomical features, but especially due to its basifixed anthers with expanded connectives. *Tripogandra* *s.l.* can be differentiated from *Callisia* *s.l.* by its inconspicuous cincinni bracts and flowers with a 60° display torsion, coupled with the presence of dimorphic stamens, and dorsifixed anthers.

### Reproductive and biogeographical patterns in *Tradescantia* *s.s.*

The present study recovered the same five clades pattern as Burns et al. (2011), and Hertweck and Pires (2014), despite the slightly different relationship between the three clades of Core *Tradescantia* (Figs 3–5). The present results also reveal that more than 60% of the non-monospecific sections proposed by Hunt (1975, 1980, 1986b) for *Tradescantia* are paraphyletic. The congruence between the phylogenetic hypotheses recovered based on the two-previous molecular datasets, with the hypothesis provided by the herein presented multiapproach dataset is indeed intriguing, but not at all surprising. All three topologies evidence an ecological and biogeographical pattern for each of the five main lineages of *Tradescantia*, reflecting on the probable relevance of these features in the evolutionary history of the genus. This data also corroborates the phylogenetic hypothesis proposed for the genus by Martínez and Martínez (1993), based exclusively on phytochemical characters. This scenario suggests a South American origin for *Tradescantia* in the Atlantic Forest domain, with a subsequent occupation of the Andes, Central America and the West Indies by the *Campelia* clade, and a latter occupation of North America, in Seasonally Tropical Dry Forests (STDF) and savanna formations by Core *Tradescantia*, with some probable dispersions to STDF in Central and South America.

The *Austrotradescantia* clade is restricted to South America, more precisely to Southeastern and Southern Brazil (i.e. Brazilian Atlantic Forest, especially in moist areas), Argentina, Paraguay, Uruguay, and Bolivia. Tradescantia sect. Austrotradescantia is invariably recovered as monophyletic, regardless of the number of species sampled for the group. The *Austrotradescantia* clade is also consistently recovered as sister to the remaining species of *Tradescantia* *s.s.* The species of the *Austrotradescantia* clade possess a pronounced floral conservatism, with flat and small flowers, petals elliptic to ovate to broadly ovate, commonly white but sometimes in shades of pink and lilac, equal stamens with basally densely bearded moniliform hairs, glabrous gynoecium, and punctate stigma. During field and cultivation studies, flowers from this group were observed to rarely be visited by any insects at all. The few observed insects consist of generalist pollen-collectors such as hoverflies (Diptera, Syrphidae) and less commonly sweatbees (Hymenoptera, Halictidae) (pers. observ.), and point to a non-specialized floral syndrome, which might also lead to the formation of some putative hybrids observed during the development of the taxonomic revision of the group (Pellegrini 2015) and evidenced by Martínez (1984) with his cytological work on the group.

The *Campelia* clade is mostly restricted to understory environments of SDTF and rainforests, ranging from Mexico to Argentina. The species in this clade possess a wide range of variation regarding vegetative morphology, but share similar reproductive specializations (e.g. the presence of coflorescences, spathaceous cincinni bracts, distinct floral display position, bigger flowers, sepals zygomorphic and partially connate, petals variously colored and shaped, showy androecium with very enlarged connectives, white pollen *in vivo*, and semilateral embryotega), that might indicate a key shift in the reproductive strategy in this lineage. These reproductive features might help in the attraction of pollinators and could also be related in avoiding hybridization (pers. observ.). Aside from that, *T. zanonia* is the only species in the genus confirmed to be zoochoric dispersed, with its berry-like capsules being dispersed by birds. Despite the unique collection of reproductive peculiarities in the *Campelia* clade, no reproductive study has ever focused on it. As in most Commelinaceae, almost nothing is known regarding the group’s reproductive biology (Pellegrini and Faden 2017).

Core *Tradescantia* has Central and North America (especially Mexico and southern USA) as its diversity center, with almost all of its species being restricted to deserts, savanna formations, or STDF. In Core *Tradescantia* the increase in floral size is obvious in almost all species, being the clade with the most widely cultivated species due to their showy flowers. Despite the flowers ranging from medium to very large in this group, little floral specialization is observed, with androecium and gynoecium morphology being rather constant. Almost all floral specializations in Core *Tradescantia* are also synapomorphic to the group. Alternatively, most of the reproductive diversity recorded in this group is related either to synflorescence structure or petal conation. The *Mandonia* clade is especially interesting, ranging from South to North America, but with a peculiarly disjunct distribution restricted to the dry environments across the American continent. Its species seem to be greatly adapted to seasonality and longer dry periods, since the tuberous roots are extremely well-developed in all species (Fig. 9A). The inflorescence architecture in the *Mandonia* clade is unique in the genus, where a great number of axillary coflorescences is produced, and the main florescence is consistently sessile, with reduced cincinni bracts. This collection of features, gives the impression that the fertile individuals are large, many-branched thyrse with alternate double-cincinni. Furthermore, the flowers in the *Mandonia* clade possess characteristically long filaments and styles, that become spirally-coiled at post-anthesis (Fig. 9H–K). Nonetheless, since it is hard to infer how these features may affect or may have been selected by a shift in the group’s pollination syndrome, this group should also be the focus of reproductive biology studies (pers. observ.). The *Setcreasea* and *Tradescantia* clades are restricted to North America, with few species naturally reaching Central America. Both clades possess rather similar floral morphologies, differing mostly in the shape of their perianths (i.e. tubular in the *Setcreasea* clade *vs.* flat in the *Tradescantia* clade). Once again, the species from these two groups seem to present a generalist floral syndrome, with its flowers being visited by a wide range of insects, such as: hoverflies, sweatbees, honeybees (Hymenoptera, Apidae), bumblebees (Hymenoptera, Apidae), and occasional small unidentified beetles (pers. observ.).

## Taxonomy

Based on the herein presented results, coupled with previously published molecular based phylogenetic studies, I recircumscribe subtribe Tradescantiinae to include subtribe Thyrsantheminae. This expanded Tradescantiinae is equivalent to the exclusively Neotropical *Tradescantia* alliance, as proposed by Hertweck and Pires (2014). In order to facilitate the identification of the 11 genera accepted in Tradescantiinae *s.l.*, I present an identification key to the expanded subtribe. Furthermore, I transfer *T. guatemalensis* to *Elasis*, in order to recognize a morphologically cohesive and monophyletic *Tradescantia*. Finally, I present a new infrageneric classification for *Tradescantia* *s.s.*, organizing it in five monophyletic subgenera. As opposed to all previous infrageneric classifications for *Tradescantia* (i.e. Clarke 1881; Brückner 1930; Hunt 1975, 1980, 1986), I have chosen to organize the genus in subgenera, instead of sections. This decision is made from a nomenclatural and taxonomic perspective, since no subgenera were ever proposed for *Tradescantia*, and names have no priority outside their original rank of publication (McNeill et al. 2012 Art. 11.2). Thus, I was able to select the names that better characterize the clade, also avoiding names that might increase the existing taxonomic confusion in *Tradescantia* (Pellegrini et al. 2016). Finally, I provide an identification key to the subgenera, and characterize each one of them, also providing the approximate number of species and names accepted in each one of them.

### Subtribe Tradescantiinae

Taxon classificationPlantaeCommelinalesCommelinaceae

Rohw., Abh. Auslandsk. 61, Reihe C, Naturwiss. 18: 144. 1956.

Fig. 6

#### Type genus.

*Tradescantia* L.

### Subtribe Thyrsantheminae

Taxon classificationPlantaeCommelinalesCommelinaceae

Faden & D.R.Hunt, Taxon 40(1): 26. 1991 syn. nov.

#### Type genus.

*Thyrsanthemum* Pichon.

#### Diagnosis.

*Herbs* chamaephytes or geophytes, base definite or indefinite, perennial or annual, terrestrial, rupicolous or epiphytes. *Roots* thin and fibrous or thick and tuberous. *Rhizomes* absent. *Stems* all aerial, rarely both underground and aerial stems present. *Leaves* sessile to subpetiolate; distichously or spirally-alternate, evenly distributed along the stem or congested at the apex of the stem; sheaths closed, rarely split open at maturity; blades flat to falcate and/or complicate, base symmetrical or asymmetrical. *Synflorescences* terminal or axillary in the distal portion of the stems, sometimes exclusively axillary, composed of a solitary main florescence or a main florescence with 1–several coflorescences. *Inflorescences (main florescences)* consisting of a variously modified thyrse, sometimes extremely reduced to few cincinni, inflorescence bract leaf-like or hyaline, tubular and inconspicuous, rarely spathaceous; peduncle bracts present or not; supernumerary bracts present or not; cincinni bracts frondose (leaf-like or spathaceous), bracteose, rarely reduced to hyaline crests, saccate or not at base, free from each other or not; cincinni alternate, fasciculate, verticillate or subopposite, free to fused back to back, sessile, contracted or elongated, bracteoles inconspicuous or expanded, imbricate or not, sometimes completely involving the cincinnus. *Flowers* bisexual, sometimes staminate, rarely pistillate, actinomorphic zygomorphic, chasmogamous, flat or tubular, when present floral tube infundibuliform to hypocrateriform, rarely campanulate; pedicel gibbous at apex or not, upright or geniculate at anthesis and pre-anthesis, deflexed at post-anthesis; sepals equal or unequal, free to conate, membranous or chartaceous, rarely fleshy, cucullate, dorsally keeled or not, persistent in fruit; petals sessile or clawed, equal, rarely subequal, free to conate; stamens (1–3–)6, arranged in two series, equal or subequal or unequal, all fertile or not, filaments free from each other, free from the petals or epipetalous, rarely connate producing a petalo-staminal ring, straight or sigmoid at anthesis, straight or spirally-coiled at post-anthesis, bearded or not with moniliform hairs, rarely hairs non- moniliform, when present hairs basal or medial or apical, sparse to dense, much shorter or as long as the stamens, anthers basifixed or dorsifixed, rimose, connective expanded or not, anther sacs straight or divergent; ovary sessile, variously pubescent, (1–2–)3-locular, locules equal, locules 1–several-ovulate, ovules uniseriate, style straight or sigmoid at anthesis, straight or spirally-coiled at post-anthesis, obconical or cylindrical at base, cylindrical at length, conical or cylindrical to obconical at the apex, stigma punctate or truncate to capitulate or capitate to trilobate. *Capsules* smooth, glabrous, loculicidal, (2–)3-valved, rarely indehiscent, sometimes apiculate due to persistent style base. *Seeds* exarillate, ventrally flattened or not, cleft or not towards the embryotega, testa variously ornamented, hilum punctate to elliptic, C-shaped or linear, embryotega dorsal, semilateral or lateral, conspicuous or not, with a prominent apicule or not.

#### Chromosomes.

Small, medium or large-sized, uni- or bimodal, *n*= 4–17

#### Included genera.

*Callisia* Loefl. (New World, 20 spp.); *Tripogandra* Raf. (Neotropics, ca. 22 spp.); *Tradescantia* L. emend. M.Pell. (New World, ca. 90 spp.); *Gibasis* Raf. (Neotropics, ca. 11 spp.); *Elasis* D.R.Hunt (Mexico/Guatemala/Ecuador, ca. 4 spp.); *Matudanthus* D.R.Hunt (Mexico, 1 sp.); *Thyrsanthemum* (Mexico, 3 spp.); *Gibasoides* D.R.Hunt (Mexico, 1 sp.); *Tinantia* Scheidw. (Texas/Neotropics, 13 spp.); *Weldenia* Schult.f. (Mexico/Guatemala, 2 sp.); *Sauvallea* C.Wright *ex* Hassk. (Cuba, 1 sp.).

#### Notes.

Subtribe Tradescantiinae (*sensu* Faden and Hunt 1991) is composed by *Callisia* *s.l.*, *Gibasis*, *Tradescantia*, and *Tripogandra*. The subtribe was characterized by its main florescences reduced to a double-cincinni, fused back to back, or by two to several stipitate and geniculate cincinni arranged in an umbellate thyrse (Faden and Hunt 1991; Panigo et al. 2010). In this old circumscription of Tradescantiinae, the cincinni are generally contracted, as opposed to the elongated cincinni in subtribe Thyrsantheminae (Faden and Hunt 1991). Thyrsantheminae represents a rather heterogeneous assemble of genera, with no clear morphological feature linking these groups together. Not surprisingly, both subtribes have been consistently recovered as non-monophyletic, due to the inclusion of *Elasis* in Tradescantiinae *s.s.*, and to the remaining genera of Thyrsantheminae being recovered in two independent lineages (Bergamo 2003; Evans et al. 2003; Wade et al. 2006; Burns et al. 2011; Zuiderveen et al. 2011; Hertweck and Pires 2014; Pellegrini et al. unpublished data; Fig. 4B). Nonetheless, if both subtribes are combined, they become equivalent to the *Tradescantia* alliance (*sensu* Hertweck and Pires 2014) and monophyletic (Evans et al. 2003; Wade et al. 2006; Burns et al. 2011; Hertweck and Pires 2014; Pellegrini et al. unpublished data). This clade is exclusively Neotropical, having pollen grains with rugose to rugose-insulate tectum as its synapomorphy (Poole and Hunt 1980; pers. observ.).

### Key to the genera of Tradescantiinae *s.l.*

**Table:** 

1	Main florescence a double-cincinni, cincinni opposite to subopposite, fused back to back or rarely only basally fused	**2**
–	Main florescence a perfect or umbelliform thyrse (i.e. with abbreviated main axis), sometimes reduced to a solitary cincinnus, cincinni alternate, verticillate or fasciculate, free	**4**
2	Main florescence subtended by a 2–3(–4) frondose cincinni bracts, bracts sometimes reduced (if reduced, inflorescences sessile and predominantly axillar); seeds ellipsoid to reniform, hilum linear	***Tradescantia* L. emend. M.Pell.** (Figs 6O–T, 7–14)
–	Main florescence subtended by a pair of reduced (i.e. bracteose) or vestigial cincinni bracts (i.e. consisting of a membranous crest at the base of each cincinnus); seeds triangular to round-triangular or tetrahedral, hilum punctiform to elliptic	**3**
3	Cincinni bracts vestigial; flowers with a 60° display torsion, stamens dimorphic, rarely subequal or antepetalous whorl absent, anthers dorsifixed; pollen with verrucose-granulose tectum	***Tripogandra* Raf. *s.l.*** (Fig. 6I–J)
–	Cincinni bracts reduced; flowers without display torsion, stamens monomorphic, anthers basifixed; pollen with clavate or rugulose tectum	***Callisia* Loefl. *s.l.*** (Fig. 6D–H)
4	Bracteoles conspicuous and persistent; flower zygomorphic, petals unequal, anthers dimorphic, filaments and style sigmoid to J-shaped	***Tinantia* Scheidw.** (Fig. 6A)
–	Bracteoles much reduced and sometimes caduceus; flowers actinomorphic, petals equal, anthers monomorphic, filaments and style straight	**5**
5	Stem subterraneous; leaves congested forming a rosette; sepals and petals fused, each forming a long and narrow tube, filaments connate to the corolla tube forming a petalo-staminal ring, glabrous, anthers basifixed; pollen domed-insulate	***Weldenia* Schult.f.** (Fig. 6C)
–	Stem aerial; leaves generally evenly distributed along the stem; sepals and petals free, filaments free, bearded with moniliform hairs, anthers dorsifixed; pollen rugulose	**6**
6	Main florescence 1-flowered, basal bract spathaceous, cincinnus contracted; petals subequal, gynoecium 2-locular	***Sauvallea* C.Wright *ex* Hassk.**
–	Main florescence (1–)many-flowered, basal bract leaf-like or tubular and hyaline, cincinni elongate; petals equal, gynoecium 3-locular	**7**
7	Basal bract leaf-like, bracteoles caduceus; flowers sessile to subsessile, stamens subequal, anther sacs C-shaped; embryotega lateral to semilateral	**8**
–	Basal bract tubular and hyaline, bracteoles persistent; flowers distinctively pedicellate, stamens equal, anther sacs elliptic; embryotega dorsal	**9**
8	Main florescence thyrsiform, cincinni alternate	***Thyrsanthemum* Pichon** (Fig. 6B)
–	Main florescence umbelliform, cincinni verticillate	***Gibasoides* D.R.Hunt**
9	Cincinni geniculate, long stipitate; connective expanded, anther sacs divergent	***Gibasis* Raf.** (Fig. 6N)
–	Cincinni upright, sessile to subsessile; connective inconspicuous, anther sacs parallel	**10**
10	Roots thin and fibrous; cincinni subsessile; petals lacking tannin cells	***Elasis* D.R.Hunt** (Fig. 6K–L)
–	Roots tuberous; cincinni sessile; petals with tannin cells	***Matudanthus* D.R.Hunt** (Fig. 6M)

### 1. Elasis

Taxon classificationPlantaeCommelinalesCommelinaceae

D.R.Hunt, Kew Bull. 33(2): 332. 1978.

Fig. 6K–L

Tradescantia sect. Coholomia D.R.Hunt, Kew Bull. 35(2): 440. 1980., **Syn. nov.** Type species. T. guatemalensis C.B.Clarke *ex* Donn.Sm. [≡ E. guatemalensis (C.B.Clarke *ex* Donn.Sm.) M.Pell.].

#### Type species.

*Elasis hirsuta* (Kunth) D.R.Hunt (≡ *Tradescantia hirsuta* Kunth).

#### Comments.

A taxonomic revision of *Elasis* is currently being prepared (Pellegrini and Hunt, in prep.) and should address pending taxonomic problems in the genus. In gross flower morphology, *Elasis* can be confused with *Tradescantia* and most of its segregate genera (i.e. some species of *Callisia*, *Gibasis*, *Matudanthus*, *Thyrsanthemum*, *Gibasoides*, and *Sauvallea*). Nonetheless, *Elasis* can be easily differentiated from these genera due to its sessile inflorescence, with 1–several fasciculate, non-geniculate cincinni, pedicellate flowers, petals lacking tannin cells, inconspicuous connectives, and truncate stigma (Fig. 5, clade C; Fig. 6K–L).

### 1.1. Elasis guatemalensis

Taxon classificationPlantaeCommelinalesCommelinaceae

(C.B.Clarke ex Donn.Sm.) M.Pell. comb. nov.

urn:lsid:ipni.org:names:77166529-1

Fig. 6L

Tradescantia guatemalensis C.B.Clarke *ex* Donn.Sm., Bot. Gaz. 18(6): 210. 1893. Lectotype (designated here). GUATEMALA. Jalapa: Laguna de Ayarza, fl., fr., Sep 1892, Heyde & Lux 3886 (US barcode US00045211!; isolectotypes: NY barcode NY00039636!, P barcode P02173850!)

#### Nomenclatural notes.

Hunt (1994) designated the specimen *Heyde & Lux 3515* (K) as the lectotype for *T. guatemalensis*. This specimen was indeed examined by Clarke, being annotated as a new species and presenting drawings with diagnostic features for the new species. Nonetheless, after carefully analyzing the protologue and the collections of K, NY and P, I noticed that the collector’s number for the specimen at K is actually “3519”, which is annotated in the specimen by the original collectors and by Clarke, instead of “3515” as cited by Smith (1893). This lead me to conclude that Smith (1893) had limited access to this specimen, and probably did not base his diagnosis on it. Thus, the lectotype designated by Hunt (1994) must be disregarded, since the collector number is incorrect, and the specimen chosen by him does not correspond to a specimen of *T. guatemalensis* and was not cited in the protologue. On the other hand, the collection Heyde & Lux 3886 was clearly available to Smith, being housed at the NY, P and US herbaria, and was most probably studied by him. The US specimen is greatly preserved, presenting flowers and fruits, and is a good option for a lectotype. Thus, it is here designated as the lectotype of *E. guatemalensis*.

### 2. Tradescantia

Taxon classificationPlantaeCommelinalesCommelinaceae

L., Species Plantarum 1: 288. 1753, emend. M.Pell.

Figs 6O–T , 7 , 8 , 9 , 10 , 11 , 12 , 13 , 14

#### Type species.

*Tradescantia virginiana* L.

#### Description.

*Herbs* chamaephytes or geophytes, base definite or indefinite, perennial, frequently succulent, terrestrial, rupicolous or epiphytes. *Roots* thin and fibrous or thick and tuberous. *Rhizomes* absent. *Stems* prostrate with ascending apex or erect, herbaceous to succulent, rarely fibrous, unbranched to branched only at base or little to densely branched, rooting at the basal nodes or at the distal ones when they touch the substrate. *Leaves* sessile to subpetiolate; distichously or spirally-alternate, evenly distributed along the stem or congested at the apex of the stem; sheaths closed or split open at maturity; ptyxis involute or convolute; blades flat to falcate and/or complicate, base symmetrical or asymmetrical, midvein conspicuous or not, secondary veins conspicuous or not. *Synflorescences* terminal or axillary in the distal portion of the stems, sometimes exclusively axillary, composed of a solitary main florescence or a main florescence with 1–several coflorescences. *Inflorescences (main florescences)* consisting of a pedunculate double-cincinni fused back to back, sometimes the main florescence composed of 3(–5) cincinni fused back to back, rarely reduced to a solitary cincinnus in axillary inflorescences; inflorescence bract hyaline, tubular, inconspicuous; peduncle bracts present or not; supernumerary bracts present or not; cincinni bracts leaf-like, spathaceous, sometimes reduced (bracteose), generally differing from the leaves mostly only in size, similar to unequal to each other, saccate or not, free from each other; cincinni sessile, contracted, bracteoles inconspicuous or expanded, imbricate or completely involving the cincinnus, linear-triangular to triangular or flabellate, hyaline. *Flowers* bisexual, actinomorphic or slightly zygomorphic due to the unequal sepals and geniculate pedicels, chasmogamous, flat or tubular, when present floral tube infundibuliform to hypocrateriform, rarely campanulate; pedicel gibbous at apex or not, upright or geniculate at anthesis and pre-anthesis, deflexed at post-anthesis; sepals equal or unequal, free to conate, membranous or chartaceous, rarely fleshy, cucullate, dorsally keeled or not, margin hyaline, apex acute, persistent in fruit; petals sessile or clawed, equal, free to conate, blade flat or plicate; stamens 6, arranged in two series, equal or subequal, filaments free from each other, free from the petals or epipetalous, straight or spirally-coiled at anthesis and post-anthesis, bearded or not with moniliform hairs, when present hair basal or medial or apical, sparse to dense, much shorter or as long as the stamens, anthers basifixed, rimose, connective rhomboid or cordate to sagittate to linearly-tapered or quadrangular to rectangular, generally yellow, but also white or orange or red or pink or lilac, anther sacs ellipsoid or round or C-shaped, divergent, generally yellow, sometimes also white or pink or lilac, pollen generally yellow, sometimes white; ovary sessile, subglobose, white, glabrous, 3-locular, locules equal, locules (1–)2-ovulate, ovules uniseriate, style straight at anthesis, straight or spirally-coiled at post-anthesis, variously colored, obconical or cylindrical at base, cylindrical at length, conical or cylindrical to obconical at the apex, stigma punctate or truncate to capitulate or capitate to trilobate, pistil shorter or the same length or longer than stamens. *Capsules* subglobose to globose, light to medium brown when mature, loculicidal, 3-valved, sometimes apiculate due to persistent style base. *Seeds* exarillate, 1–2 per locule, reniform to ellipsoid to narrowly trigonal, ventrally flattened, cleft or not towards the embryotega, testa smooth to faintly rugose to rugose or scrobiculate or costate with ridges radiating from the embryotega, hilum linear, embryotega dorsal or semilateral, conspicuous or not, generally covered by a cream farina, with a prominent apicule or not.

#### Habitat, distribution and ecology.

**Figure 7.**
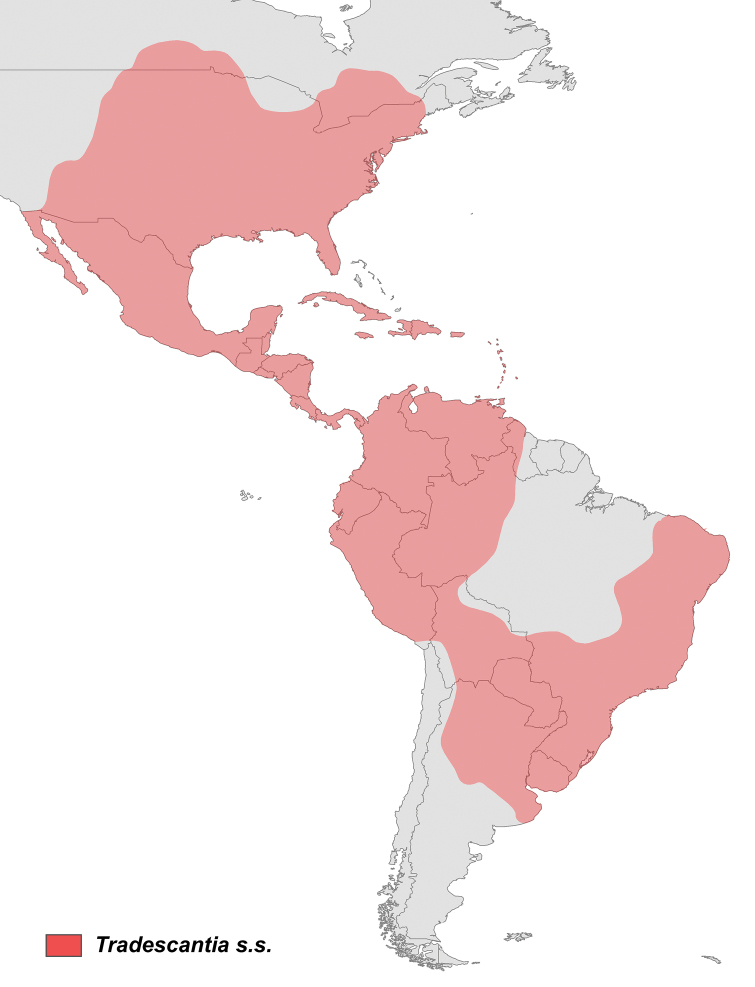
Distribution of *Tradescantia* L. emend. M.Pell.

Neotropical, ranging from southern USA to Argentina, having Mexico and Central America as its diversity center (Fig. 7). *Tradescantia*, as evidenced by its wide distribution and morphological variation, grows in a wide range of environments. The main habitat and ecological traits of the genus are discussed below, under each of the five proposed subgenera.

#### Phylogenetic placement and circumscription.

With the present recircumscription of *Tradescantia*, the genus seems to be finally monophyletic and easily morphologically characterized. Based on molecular and combined data, *Tradescantia* is sister to the clade composed by *Gibasis*+*Elasis*, with these three genera being sister to the clade composed by *Tripogandra* *s.l.* and all lineages of the polyphyletic *Callisia* (Bergamo 2003; Evans et al. 2003; Wade et al. 2006; Burns et al. 2011; Zuiderveen et al. 2011; Hertweck and Pires 2014; Pellegrini et al. in prep.). This whole clade (see Fig. 4B) is morphologically supported by the presence of an inconspicuous, hyaline and tubular basal bract, and the main florescence reduced to a double-cincinni fused back to back. As stated in this study and thoroughly discussed by Hunt (1975, 1980, 1983, 1986b), the circumscription of *Tradescantia* has been the focus of great discussion since its description by Linnaeus (1753). Since the circumscription adopted in the present study does not match any of the previous circumscriptions, I propose an amendment to the description to the genus, to assure taxonomic clarity and aid taxonomists to recognize it.

#### Growth form and life cycle.

Despite common misconception, almost all species of *Tradescantia* are perennial herbs, lacking a true rhizome. In some species of *Tradescantia*, some portions of the stems might become non-chlorophyllate due to shading and produce shortened internodes from being underground. Nonetheless, these stems lack cataphylls and the anatomic characterization needed for them to be correctly classified as rhizomes. Thus, the only perennation structures known for the genus are the well-known tuberous roots, characteristic of *T. commelinoides*, *T.* subg, *Mandonia* (Fig. 12A), T. subg. Setcreasea, and T. subg. Tradescantia (Fig. 14A). The only truly annual species are restricted to T. subg. Tradescantia, but this character is not typical of the subgenus as a whole. Species appearing annual lack conspicuously tuberous roots and occur in the northernmost range of the genus in temperate zones, which may not be hardy during harsh and snowy winters.

#### Key to the subgenera of *Tradescantia*

**Table:** 

1	Stems prostrate with ascending apex or erect; sepals generally all keeled, filaments densely bearded at the base with long moniliform hairs, stigma punctate; embryotega inconspicuous	**Tradescantia subg. Austrotradescantia** (Figs 6O, 9)
–	Stems erect, rarely prostrate with ascending apex; sepals rarely keeled, if present keel restricted to the dorsal sepal, filaments glabrous to sparsely bearded at mid-length, rarely at the base or apex with short moniliform hairs, stigma truncate to capitulate or capitate to trilobate; embryotega with a conspicuous apicule	**2**
2	Roots thin and fibrous, rarely tuberous; inflorescence composed by the main florescence and generally 1–many coflorescences, peduncle bracts commonly present, cincinni bracts spathaceous; stamens subequal, connectives cordate to sagittate to linear-tapered, rarely rhomboid, anther sacs globose, rarely ellipsoid, pollen white; embryotega semilateral	**Tradescantia subg. Campelia** (Figs 6P–Q, 10)
–	Roots fleshy to tuberous; inflorescence composed only by the main florescence, peduncle bracts never present, cincinni bracts leaf-like or reduced; stamens equal, connectives quadrangular to rectangular, rarely slightly rhomboid to slightly sagittate, anther sacs elliptic to curved, pollen yellow; embryotega dorsal	**3**
3	Main florescences sessile, mainly axillary, cincinni bracts reduced; sepals chartaceous, filaments and style spirally-coiled at post-anthesis, style ½ longer than the stamens	**Tradescantia subg. Mandonia** (Figs 6R, 12)
–	Main florescences pedunculate, rarely sessile, terminal, cincinni bracts expanded and leaf-like; sepals membranous, filaments and style straight at post-anthesis, style equal or shorter than the stamens	**4**
4	Leaves lanceolate to ovate to rotund, rarely cylindrical, base obtuse to slightly cordate; pedicel apically gibbous, flowers tubular, stamens epipetalous, filaments glabrous or sparsely bearded, stigmatic papillae evenly distributed in the stigma	**Tradescantia subg. Setcreasea** (Figs 6S, 13)
–	Leaves linear to acicular, base truncate to round; pedicels apically non-gibbous, flowers flat, stamens free, filaments densely bearded, stigmatic papillae restricted to the margins of the stigma	**Tradescantia subg. Tradescantia** (Figs 6T, 14)

### 2.1. Tradescantia subg. Austrotradescantia

Taxon classificationPlantaeCommelinalesCommelinaceae

(D.R.Hunt) M.Pell., comb. et stat. nov.

urn:lsid:ipni.org:names:77166527-1

Figs 6O , 9

Tradescantia sect. Austrotradescantia D.R.Hunt, Kew Bull. 35(2): 440. 1980. Type species. T. fluminensis Vell.
Tropitria Raf., Fl. Tellur. 3: 68. 1837. Type species. Tropitria crassula (Link & Otto) Raf. (≡ T. crassula Link & Otto).

#### Description.

*Herbs* chamaephytes, base definite or indefinite, perennial, frequently succulent, terrestrial, rupicolous or epiphytes. *Roots* thin, fibrous. *Stems* prostrate with ascending apex or erect, herbaceous to succulent, rarely fibrous, little to densely branched, rooting at the basal nodes or at the distal ones when they touch the substrate. *Leaves* sessile to subpetiolate; distichously or spirally-alternate, evenly distributed along the stem, rarely congested in a rosette; sheaths closed; blades flat to falcate and/or complicate, base asymmetrical, midvein conspicuous, rarely inconspicuous, adaxially impressed, abaxially prominent, rounded, secondary veins conspicuous or inconspicuous. *Synflorescences* terminal or axillary in the distal portion of the stems, composed of a solitary main florescence, 1–4 per leaf axis. *Inflorescences (main florescences)* consisting of a pedunculate double-cincinni fused back to back; inflorescence bract hyaline, tubular, inconspicuous; peduncle bracts absent; supernumerary bracts rarely present; cincinni bracts leaf-like, rarely spathaceous, differing from the leaves mostly only in size, similar to unequal to each other, saccate or not, free from each other; bracteoles inconspicuous, imbricate, linear-triangular to triangular, hyaline. *Flowers* bisexual, actinomorphic, flat (not forming a floral tube); pedicel gibbous at apex, upright at anthesis and pre-anthesis, deflexed at post-anthesis; sepals equal, free, chartaceous, ovate, dorsally keeled or not, apex acute; petals sessile, equal, free, elliptic to ovate to broadly ovate, flat or plicate, base cuneate to obtuse, margin entire, apex acute; stamens 6, arranged in two series, equal, filaments free from the petals, straight at anthesis and post-anthesis, basally densely bearded with moniliform hairs, hairs as long as the stamens, white, anthers with connective rhomboid, yellow, anther sacs ellipsoid, yellow, pollen yellow; ovary white, glabrous, locules 2-ovulate, style straight at anthesis and post-anthesis, white, obconical at base, conical at the apex, stigma punctate, pistil longer than or the same length as the stamens. *Capsules* subglobose to globose, light to medium brown when mature, glabrous, loculicidal, 3-valved, sometimes apiculate due to persistent style base. *Seeds* 1–2 per locule, ellipsoid to narrowly trigonal, ventrally flattened, cleft or not towards the embryotega, testa costate to rugose with ridges radiating from the embryotega, embryotega dorsal, relatively inconspicuous, without a prominent apicule.

#### Habitat, distribution and ecology.

**Figure 8.**
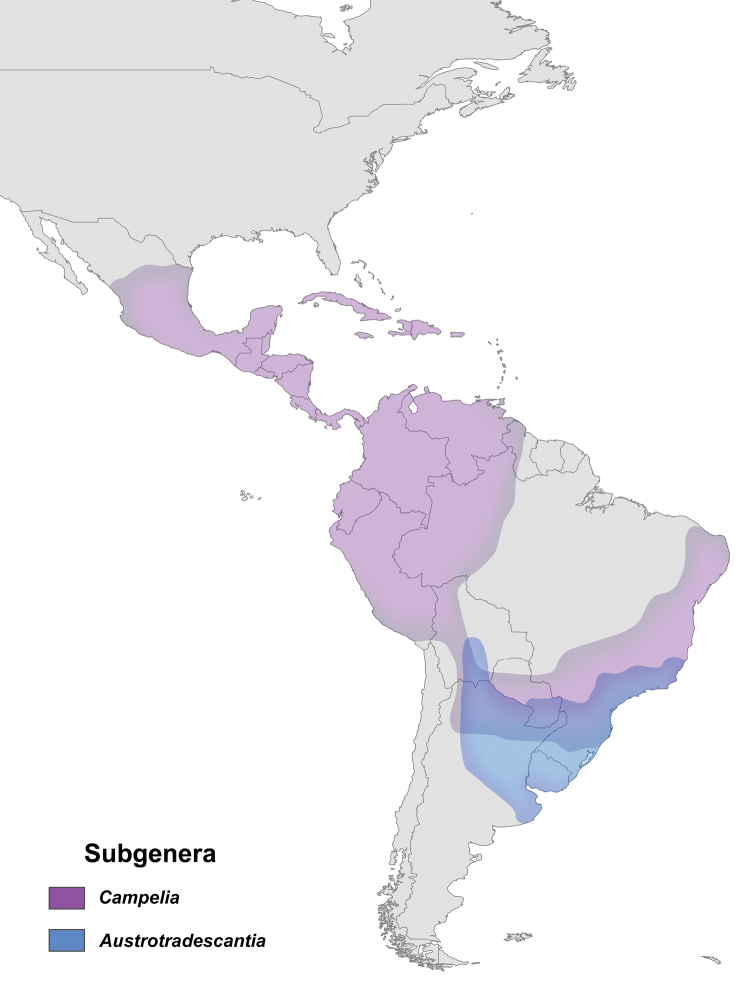
Distribution of Tradescantia subg. Austrotradescantia (D.R.Hunt) M.Pell. in blue, and of T. subg. Campelia (Rich.) M.Pell. in purple.

As stated by Hunt (1980), T. subg. Austrotradescantia is the only exclusively South American group in the genus, occurring in Bolivia, Brazil, Paraguay, Uruguay and Argentina (Fig. 8). Its species can be found growing understory in moist and shady forests in the Atlantic Forest domain, open fields, rocky outcrops, and are especially common in disturbed areas.

#### Included species.

The subgenera is composed by ca. 15 species, namely: *Tradescantia cerinthoides* Kunth, *T. chrysophylla* M.Pell., *T. crassula* Link & Otto, *T. cymbispatha* C.B.Clarke, *T. fluminensis* Vell., *T. mundula* Kunth, *T. seubertiana* M.Pell., *T. tenella* Kunth, *T. umbraculifera* Hand.-Mazz., and *T. valida* G.Brückn. Accepted names and total of accepted species for T. subg. Austrotradescantia will be separately dealt in the taxonomic revision for the group, including the formal description of two new species.

#### Comments.

**Figure 9.**
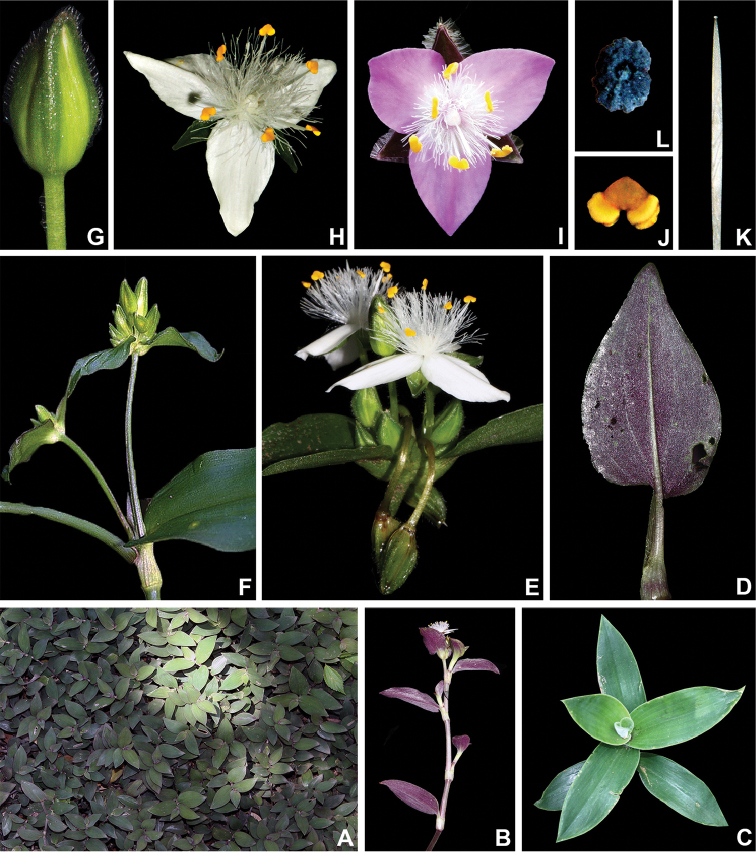
Tradescantia subg. Austrotradescantia (D.R.Hunt) M.Pell. **A–C** habit: **A** prostrate, mat-forming habit of *T. cymbispatha* C.B.Clarke **B** detail of a branch of *T. cymbispatha*, showing the distichously-alternate leaves **C** young specimen of *T. cerinthoides* Kunth, showing the rosette habit and spirally-alternate leaves **D** subpetiolate leaf of *T. tenella* Kunth **E–F** inflorescence: **E** inflorescence of *T. fluminensis* Vell., showing the leaf-like, saccate cincinni bracts, and deflexed pedicels at post-anthesis **F** synflorescence of *T. umbraculifera* Hand.-Mazz., showing two inflorescence per leaf axil, and spathaceous, saccate cincinni bracts **G** floral bud of *T. fluminensis*, showing three keeled sepals **H–I** flowers: **H** flower of *T. fluminensis*, showing the white, plicate petals **I** flower of *T. cerinthoides*, showing the pink, flat petals **J** anther of *T. fluminensis*, showing the rhomboid connective and elliptic anther sacs **K** style of *T. fluminensis*, showing the punctate stigma **L** seed of *T. cerinthoides*, showing the costate testa cleft towards the embryotega, and the inconspicuous embryotega. All photos by M.O.O. Pellegrini.

Tradescantia subg. Austrotradescantia can be easily recognized by its generally distichously-alternate leaves (a character uncommon for the genus; Fig. 9A & B), sepals elliptic to broadly elliptic, all keeled (Fig. 9G); filaments basally, densely bearded with long moniliform hairs (Fig. 9E); style obconic at base and conic at apex, stigma punctate with type D papillae (Owens and Kimmins 1981; Fig. 9K); seeds with costate testa, and relatively inconspicuous embryotega (Fig. 9L). Added to these morphological characters, the small bimodal and numerous chromosomes (*n*= 10–numerous), and a unique chemical profile, set this group apart from the other four subgenera. A complete taxonomical revision of this subgenus is in the works by me (Pellegrini 2015, in prep), and should provide the necessary tools for proper species identification and name application in the group. Regarding taxonomically informative characters, leaf morphology can easily differentiate the two observed morphological groups. The presence of a definite and indefinite base is also helpful and probably connected to the understory habit of many species. Aside from that, cincinni bracts are extremely useful for species delimitation. They can be either saccate or not at base (see below), leaf-like or spathaceous (e.g. *T. umbraculifera* and *T. valida*), and the symmetry between both cincinni bracts is also very useful in differentiating some closely related species. Furthermore, *T. valida* is the only species in T. subg. Austrotradescantia to possess supernumerary bracts.

Two morphological groups can be clearly observed in T. subg. Austrotradescantia, being also recovered in the present study (Figs 3, 4). The *T. fluminensis* group is composed by more delicate plants, with prostrate stems ascending at the apex, cincinni bracts saccate at base, and generally white petals. A marking exception is the *T. tenella* complex, which possess erect stems, flowers that range from white to pink, seeds with rugose testa, and hilum always shorter than ½ the length of the seeds. Nonetheless, its species generally possess subpetiolate, membranous to chartaceous leaves, and conspicuous mid and secondary veins, which are characters common to the *T. fluminensis* group (Figs 3, 4). Since the leaves vary from membranous to chartaceous to slightly fleshy, venation pattern is very useful for species differentiation inside this clade. Both *T. fluminensis* and *T. mundula* possess leaves with adaxially impressed secondary veins, while *T. cymbispatha* and *T. chrysophylla* possess inconspicuously impressed secondary veins. The species in this group occur almost exclusively in Tropical and Subtropical Rainforests, but are also commonly found growing as weedy plants throughout their distribution range.

The *T. crassula* group is composed by succulent plants (which generally grow in open areas), with erect stems, cincinni bracts not saccate at base, petals ranging from white to pink to lilac, and seeds cleft towards the embryotega. The leaves from these species are sessile and extremely succulent, with only the midvein conspicuous, and secondary veins rarely conspicuous in *T. crassula*. Nonetheless, in some individuals of *T. crassula*, *T. seubertiana* and *T. valida*, the leaves are so succulent that even the midvein is adaxially inconspicuous. Great petal color variation can be found within the same population, under the same ecological conditions, and is probably genetically controlled. These species are intimately related to the two southern biomes of South America, characterized by open and/or drier vegetation formations: the Chaco (which is part of the Dry Diagonal) and the Pampa. The species from the *T. crassula* group are morphologically very similar due to many overlapping morphological characters, and indumenta type and pattern are the most useful characters for separating these species. For the same reason, all species were recovered within a polytomy in the strict consensus (Fig. 3). In addition, *T. cerinthoides* and *T. crassula* (the two lowland species) possess a rather wide distribution range which overlaps with the narrowly distributed *T. seubertiana* and *T. valida* (both of them restricted to high elevation sites). Much vegetative variation is recorded for the two widely distributed species, but reproductive characters are key in differentiating species in this group (Pellegrini et al. 2017). Thus, I suggest the *T. crassula* group to be targeted for phylogeographic and reproductive studies to improve and deepen the understanding of taxonomic boundaries between these taxa.

### 2.2. Tradescantia subg. Campelia

Taxon classificationPlantaeCommelinalesCommelinaceae

(Rich.) M.Pell., comb. et stat. nov.

urn:lsid:ipni.org:names:77166530-1

Figs 6P–Q , 10

Tradescantia sect. Campelia (Rich.) D.R.Hunt, Kew Bull. 41(2): 404. 1986.
Campelia Rich., Démonstr. Bot.: 46. 1808.
Zanonia Cramer., Disp. Syst.: 75. 1803, nom. illeg. Type species. Zanonia bibracteata Cramer., nom. illeg. [= Tradescantia zanonia (L.) Sw.].
Gonatandra Schltdl., Linnaea 24: 659. 1851, **Syn. nov.** Type species. Gonatandra tradescantioides Schltdl. [= Tradescantia zanonia (L.) Sw.].
Sarcoperis Raf., Fl. Tellur. 2: 16. 1837, **Syn. nov.** Type species. Sarcoperis bibracteata (Cramer) Raf. [= Tradescantia zanonia (L.) Sw.].
Tradescantia sect. Cymbispatha (Pichon) D.R.Hunt, Kew Bull. 35(2): 440. 1980.
Cymbispatha Pichon, Not. Syst. 12: 224. 1946, **Syn. nov.** Type species. T. commelinoides Schult.f.
Tradescantia sect. Rhoeo (Hance) D.R.Hunt, Kew Bull. 41(2): 401. 1986.
Rhoeo Hance, Ann. Bot. Syst. 3: 659. 1852, **Syn. nov.** Type species. T. discolor L’Hér. (= T. spathacea Sw.)
Tradescantia sect. Zebrina (Schnizl.) D.R.Hunt, Kew Bull. 41(2): 404. 1986.
Zebrina Schnizl., Bot. Zeitung (Berlin) 7: 870. 1849, **Syn. nov.** Type species. Zebrina pendula Schnizl. (= T. zebrina Heynh. *ex* Bosse)
Tradescantia sect. Corinna D.R.Hunt, Kew Bulletin 41(2): 405. 1986, **Syn. nov.** Type species. Campelia standleyi Steyermark (= T. soconuscana Matuda)

#### Description.

*Herbs* chamaephytes, rarely geophytes, base definite or indefinite, frequently succulent, terrestrial, rupicolous or epiphytes. *Roots* thin, fibrous, rarely thick, tuberous. *Stems* prostrate with ascending apex or erect, herbaceous to succulent, rarely fibrous, little to densely branched, rooting at the basal nodes or at the distal ones when they touch the substrate. *Leaves* sessile to subpetiolate; distichously or spirally-alternate, evenly distributed along the stem or congested at the apex of the stems; sheaths closed; blades flat to falcate and/or complicate, base symmetrical or asymmetrical, midvein conspicuous, rarely inconspicuous, adaxially impressed, abaxially prominent, rounded, secondary veins conspicuous or inconspicuous. *Synflorescences* terminal or axillary in the distal portion of the stems, sometimes exclusively axillary, composed of a main florescence with 1–several coflorescences, rarely composed of a solitary main florescence. *Inflorescences (main florescences)* consisting of a pedunculate double-cincinni fused back to back; inflorescence bract hyaline, tubular, inconspicuous; peduncle bracts present or not, bladeless sheaths, rarely with a reduced leaf-like blade; supernumerary bracts generally present, leaf-like to slightly spathaceous, the same size as the leaves or the cincinni bracts; cincinni bracts spathaceous, similar to unequal to each other, saccate or not, flat or conduplicate, free or fused to each other, overlapping each other or not; bracteoles expanded, imbricate or completely involving the cincinnus, linear-triangular to triangular or flabellate, hyaline. *Flowers* bisexual, slightly zygomorphic due to the unequal sepals and geniculate pedicels, flat or tubular, when present floral tube infundibuliform to hypocrateriform, rarely campanulate; pedicel gibbous at apex, geniculate at anthesis and pre-anthesis, deflexed at post-anthesis; sepals unequal, free to conate, membranous or chartaceous, rarely fleshy, elliptic to broadly elliptic to obovate, dorsally keeled or not, apex obtuse or acute; petals sessile or clawed, equal, free to conate, blade elliptic to ovate to broadly ovate or rhomboid to broadly obovoid to obovoid, flat, base cuneate to obtuse, margin entire, apex acute to obtuse; stamens 6, arranged in two series, subequal, the outer whorl shorter than the inner, filaments free from the petals or epipetalous, straight at anthesis and post-anthesis, basally, medially or apically sparsely bearded with moniliform hairs, hairs shorter than the stamens, variously colored, anthers with connective cordate to sagittate to linearly-tapered, rarely rhomboid, variously colored, anther sacs round, white, sometimes pink to lilac or yellow, pollen white; ovary white, glabrous or pubescent, locules (1–)2-ovulate, style straight at anthesis and post-anthesis, variously colored, cylindrical at base, cylindrical to obconical at the apex, stigma capitate to trilobate, pistil shorter to the same length to longer than the stamens. *Capsules* subglobose to globose, light to medium brown when mature, glabrous, loculicidal, 3-valved, sometimes apiculate due to persistent style base. *Seeds* exarillate, 1–2 per locule, ellipsoid to narrowly trigonal, ventrally flattened, cleft or not towards the embryotega, testa smooth to faintly rugose to rugose or costate with ridges radiating from the embryotega, embryotega semilateral, conspicuous, with a prominent apicule.

#### Habitat, distribution and ecology.

Tradescantia subg. Campelia is the most widespread of the subgenera, ranging from Mexico to Argentina (Fig. 8). It is highly diverse in Central America and northern South America, with its species being mostly restricted to forest understories or growing in elevated open areas, such as the Andean region.

#### Included species.

Tradescantia subg. Campelia is composed by ca. 15 species, including: *Tradescantia commelinoides* Schult. & Schult.f., *T. deficiens* Brandegee, *T. gracillima* Stand., *T. grantii* Faden, *T. huehueteca* (Standl. & Steyerm.) D.R.Hunt, *T. plusiantha* Stand., *T. poelliae* D.R.Hunt, *T. praetermissa* M.Pell., *T. schippii* D.R.Hunt, *T. soconuscana* Matuda, *T. spathacea* Sw., *T. standleyi* Steyerm., *T. zanonia* (L.) Sw., and *T. zebrina* Heynh. *ex* Bosse. Despite its small number of species, a great deal of taxonomic problems and species complexes still prevents the total number of species from being known.

#### Comments.

**Figure 10.**
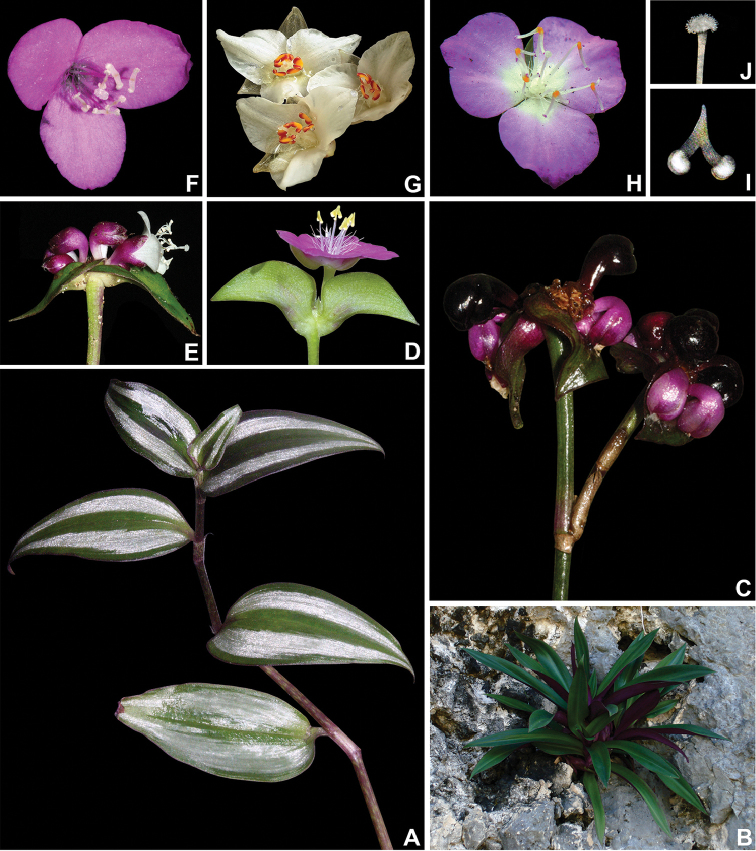
Tradescantia subg. Campelia (Rich.) M.Pell. **A–B** habit: **A** prostrate habit of *T. zebrina* Heyhn. *ex* Bosse, also showing the distichously-alternate, subpetiolate, striped leaves **B** rosette habit of *T. spathacea* Sw., also showing the spirally-alternate and sessile leaves **C–E** inflorescence: **C** synflorescence showing the presence of a coflorescence, also showing the berry-like fruits of *T. zanonia* (L.) Sw. **D** main florescence of *T. polliae* D.R.Hunt, showing the basally fused, folded, non-saccate, not overlapping, cincinni bracts and flat flower **E** main florescence of *T. zanonia*, showing the basally free, not folded, saccate, overlapping cincinni bracts, geniculate pedicels at anthesis and pre-anthesis, and the infundibuliform flower **F–H** flowers: **F** oblique view of a flower of *T. zebrina* **G** cluster of flowers of *T. spathacea*, showing the orange to red anther sacs **H** oblique view of a flower of *T. commelinoides* Schult. & Schult.f., showing the linearly-tapered connectives **I** anther of *T. zanonia*, showing the sagittate connective and round anther sac. **J** style of *T. zanonia*, showing the capitate stigma. **A, C, E, F, I, J** by M.O.O. Pellegrini, **B** by L. Gutierrez, **D** by F.A. Michelangeli, **G** by S. Neuwirth, and **H** by P. Acevedo-Rodriguez.

When *Cymbispatha* was proposed by Pichon (1946) as a new genus, he reinforced the importance of inflorescence characters in Commelinaceae, especially the shape of the cincinni bracts, and position of the embryotega on the seed. The author characterized his new genus as possessing a double-cincinni subtended by two spathaceous bracts (Fig. 10D, E), stamens of different length (Fig. 10F–H), tapered connective (Fig. 10I), and lateral embryotega; but did not note the zygomorphic calyx (Fig. 10D, E), the shape of the anther sacs, and the white pollen (Fig. 10I), all unusual characters for the genus. The present analysis reveals that important morphological characters, such as the characters listed by Pichon (1946), and previously considered as exclusive to T. sect. Cymbispatha (*sensu* Hunt 1980), are actually shared with all or most species from the T. subg. Campelia. These characters are: subequal sepals, keeled dorsal sepal, subequal stamens, and semilateral embryotega. Characters like, zygomorphic sepals, and pedicels the same size as the floral buds or sessile to subsessile are not exclusive to T. sect. Cymbispatha, but are actually homoplastic synapomorphies to the two larger clades within the *Campelia* clade (i.e. *T. commelinoides* group+*T. zebrina* group). Spathaceous bracts, the presence of supernumerary bracts, and white pollen grains *in vivo*, are also recovered in the present analysis as homoplastic synapomorphies to this subgenus. Thus, T. subg. Campelia can be differentiated from the remaining subgenera by synflorescences with one or more coflorescences, presence of peduncle bracts, presence of supernumerary bracts, spathaceous cincinni bracts (Fig. 10C–E); flowers with pedicels geniculate at anthesis and pre-anthesis (Fig. 10E), unequal sepals (Fig. 10D, E), dorsal sepal generally keeled (Fig. 10D), outer filaments shorter than the inner (Fig. 10F–H), white pollen, pistil longer than the stamens (Fig. 10E), and semilateral embryotega.

### 2.3. Tradescantia subg. Mandonia

Taxon classificationPlantaeCommelinalesCommelinaceae

(D.R.Hunt) M.Pell., comb. et stat. nov.

urn:lsid:ipni.org:names:77166528-1

Figs 6R , 11 , 12

Tradescantia sect. Mandonia D.R.Hunt, Kew Bull. 35(2): 441. 1980. Type species. Tradescantia ambigua Mart. *ex* Schult. & Schult.f.
Mandonia Hassk., Flora 54: 260. 1871., nom. illeg, non Mandonia Wedd., Bull. Soc. Bot. France 11: 50–51, t. 1. 1864.
Skofitzia Hassk. & Kanitz, Oesterr. Bot. Z. 22: 147. 1872.
Neomandonia Hutch., Fam. Fl. Pl., Monocot. 2: 57. 1934, **Syn. nov.** Type species. Mandonia boliviana Hassk. [≡ T. boliviana (Hassk.) J.R.Grant].
Tradescantia sect. Parasetcreasea D.R.Hunt, Kew Bull. 30(3): 455. 1975, **Syn. nov.** Type species. Tradescantia andrieuxii C.B.Clarke

#### Description.

*Herbs* geophytes, base definite, perennial, frequently succulent, terrestrial or rupicolous. *Roots* thick, tuberous. *Stems* erect, rarely prostrate with ascending apex, herbaceous to succulent, unbranched to little branched, rarely densely branched, rooting only at the basal nodes. *Leaves* sessile; spirally-alternate, rarely distichously-alternate, evenly distributed along the stem or congested at the apex of the stems; sheaths closed; blades flat to falcate and/or complicate, base symmetric or slightly asymmetric, midvein conspicuous, rarely inconspicuous, adaxially impressed, abaxially prominent, rounded, secondary veins conspicuous or inconspicuous. *Synflorescences* mainly axillary in the distal portion of the stems, sometimes exclusively axillary, rarely exclusively terminal, composed of a solitary main florescence. *Inflorescences (main florescences)* consisting of a sessile double-cincinni fused back to back, when terminal also pedunculate; inflorescence bract hyaline, tubular, inconspicuous; peduncle bracts absent; supernumerary bracts generally present, reduced, the same size as the leaves or the cincinni bracts, rarely leaf-like; cincinni bracts reduced, unequal to each other, non-saccate, conduplicate, free, not overlapping each other; bracteoles expanded, imbricate, linear-triangular to triangular, hyaline. *Flowers* bisexual, actinomorphic, flat or tubular, when present floral tube infundibuliform to hypocrateriform or campanulate; pedicel gibbous at apex, straight at anthesis and pre-anthesis, deflexed at post-anthesis; sepals equal, free, chartaceous, elliptic to broadly elliptic, not dorsally keeled, apex acute; petals sessile, rarely clawed, equal, free to conate, blade elliptic to ovate to broadly ovate or rhomboid to broadly obovoid to obovoid, flat, base cuneate to obtuse, margin entire, apex acute to obtuse; stamens 6, arranged in two series, equal, filaments free from the petals, rarely epipetalous, straight at anthesis, spirally-coiled at post-anthesis, medially sparsely bearded with moniliform hairs, hairs shorter than the stamens, variously colored, anthers with connective quadrangular to rectangular, rarely rhomboid, yellow, anther sacs C-shaped, rarely ellipsoid, yellow, pollen yellow; ovary pubescent, locules 2-ovulate, style straight at anthesis, spirally-coiled at post-anthesis, variously colored, cylindrical at base, cylindrical to obconical at the apex, stigma truncate to capitulate or capitate to trilobate, pistil longer than the stamens. *Capsules* broadly oblongoid to subglobose to globose, light to medium brown when mature, pubescent, loculicidal, 3-valved, sometimes apiculate due to persistent style base. *Seeds* exarillate, 1–2 per locule, ellipsoid to narrowly trigonal, ventrally flattened, not cleft towards the embryotega, testa scrobiculate to rugose, rarely costate, with ridges radiating from the embryotega, embryotega dorsal, conspicuous, with a prominent apicule.

#### Habitat, distribution and ecology.

**Figure 11.**
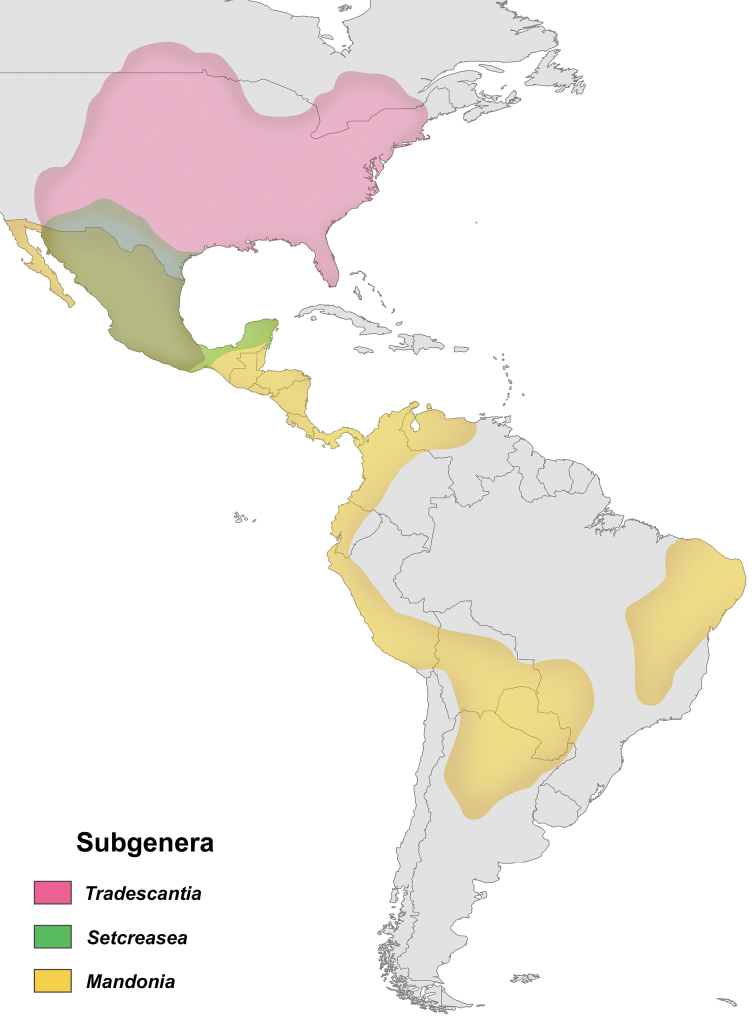
Distribution of Tradescantia subg. Mandonia (D.R.Hunt) M.Pell. in yellow, of T. subg. Setcreasea (K.Schum. & Sydow) M.Pell. in green, and Tradescantia L. subg. Tradescantia in pink.

Tradescantia subg. Mandonia is widely but disjunctively distributed across the American continent, with species occurring in North America, Central America, and South America (Fig. 11). Its species are restricted to Seasonally Dry Forests (STDF) or other dry biomes across the continent, and possess well-developed tuberous roots that allow them to perennate through the dry season. Flowering seems to be triggered by the beginning of the wet season.

#### Included species.

The subgenus includes ca. 20 species, including: *Tradescantia ambigua* Mart. *ex* Schult. & Schult.f., *T. andrieuxii* C.B.Clarke, *T. boliviana* (Hassk.) J.R.Grant, *T. burchii* D.R.Hunt, *T. crassifolia* Cav., *T. exaltata* D.R.Hunt, *T. gentryi* D.R.Hunt, *T. guiengolensis* Matuda, *T. iridescens* Lindl., *T. llamasii* Matuda, *T. masonii* Matuda, *T. mcvaughii* D.R.Hunt, *T. murilloae* Zamudio et al., *T. nuevoleonensis* Matuda, *T. peninsularis* Brandegee, *T. petricola* J.R.Grant, *T. tepoxtlana* Matuda, *T. velutina* Kunth & C.D.Bouché. A number of still undescribed species are being described, and should help better understand this taxonomically complex group (Pellegrini, Grant & Hunt, in prep.).

#### Comments.

**Figure 12.**
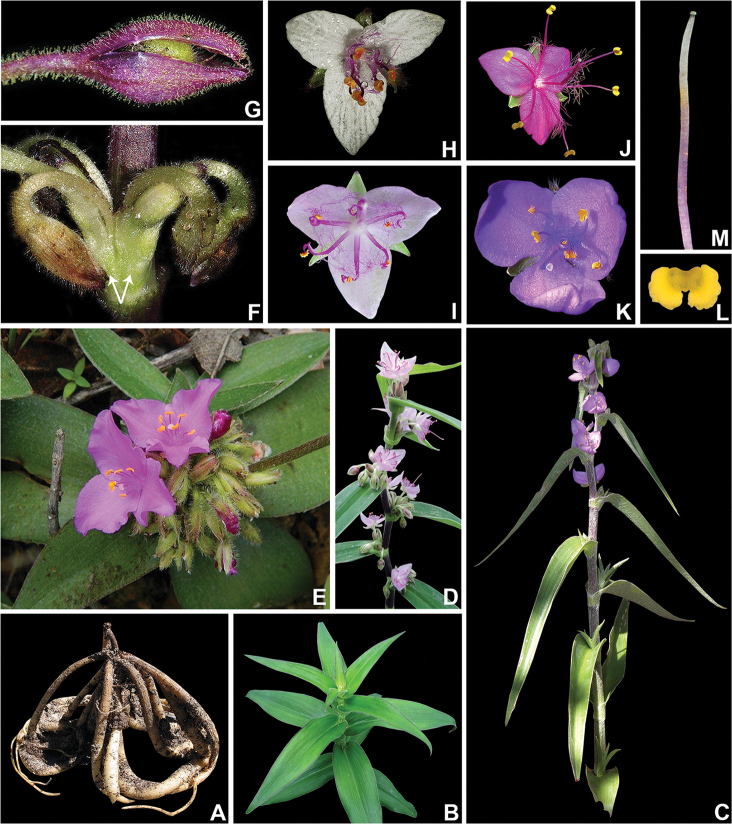
Tradescantia subg. Mandonia (D.R.Hunt) M.Pell. **A** thick tuberous roots on *T. boliviana* (Hassk.) J.R.Grant. **B–E** habit: **B** vegetative shoot of *T. ambigua* Mart. *ex* Schult. & Schult.f., showing the spirally-alternate leaves **C** flowering shoot of *T. crassifolia* Cav., showing the sessile and axillary inflorescences restricted to the apex of the branch **D** flowering shoot of *T. ambigua* Mart. *ex* Schult. & Schult.f., showing the sessile and axillary inflorescences evenly distributed along the stem **E** rosette habit of *T. iridescens* Lindl., showing the inflorescences restricted to the apex of the stem or in lateral shoots **F** detail of an inflorescence of *T. ambigua*, with arrows indicating the reduced cincinni bracts **G** post-anthesis flower of *T. boliviana*, showing glandular-pubescent sepals and hispid immature capsule **H–K** flowers: **H** flower of *T. ambigua* at anthesis **I** flower of *T. ambigua* at post-anthesis, showing the spirally-coiled filaments **J** flower of *T. boliviana* at anthesis, showing the peculiarly long filaments and style; **K** flower of *T. crassioflia* at anthesis, showing the campanulate perianth **L** anther of *T. ambigua*, showing the C-shaped anther sacs and quadrangular and slightly curved connective **M** style of *T. ambigua*, showing the capitulate stigma. **A, J** by P. Christian (RarePlants.co.uk), **B** by E.O. Moura, **C, K** by T.R. Van Devender, **D, I** by L.J. Leitão, **E** by J.C. Garcia Morales, **F, H, L–M** by M.O.O. Pellegrini, and **G** by Instituto Darwinion.

Tradescantia subg. Mandonia can be easily differentiated from the remaining subgenera due to its peculiar general morphology. It is characterized by its mainly axillary inflorescences, producing a raceme-like synflorescence, sessile main florescences (Fig. 12C–E), generally presenting supernumerary bracts, reduced cincinni bracts (rarely leaf-like in the terminal main florescences; Fig. 12F); chartaceous sepals, filaments apically spirally-coiled at post-anthesis (Fig. 12I), and style ½ time longer than the stamens, becoming spirally-coiled at post-anthesis (Fig. 12D, H–K, M). The leaves are commonly spirally-alternate and evenly distributed along the stems (Fig. 12B–D), but in few species the leaves can also be distichously-alternate or congested at the apex of the stems, forming a rosette (Fig. 12E). The architecture of the main florescence is of the double-cincinni type, although mutations seem to be much more frequent than in other subgenera. The main florescence can either be reduced to a solitary cincinnus or present more than two cincinni. Added to that, the number of cincinni bracts seems to vary greatly, although being generally hard to infer, due to great amount of reduction in the group’s inflorescence. Tubular flowers are known for few species (e.g. *T. andrieuxii*, *T. crassifolia*, and *T. guiengolensis*), while sympetaly is only described for *T. andrieuxii*. The connectives and anther sacs generally match the morphology described for Core *Tradescantia* (Fig. 12L), but some exceptions can be observed in some species and/or populations where anther morphology seems to be reminiscent of T. subg. Austrotradescantia, with rhomboid connectives and elliptic anther sacs. As expressed by Pellegrini et al. (2017), T. subg. Mandonia is a poorly understood group with species of complex delimitation, which is highlighted by the herein presented results by the poorly resolved relationship between its species. This could be easily explained by the great vegetative plasticity within species, conserved reproductive features, and lack of focused field and taxonomic studies for this subgenus. Currently, species identification greatly relies on the species allopatric distributions, with little morphological differentiation (Pellegrini et al. 2017). Further studies are surely necessary in order to better understand specific boundaries in the subgenus, and its biogeographical history.

### 2.4. Tradescantia subg. Setcreasea

Taxon classificationPlantaeCommelinalesCommelinaceae

(K.Schum. & Sydow) M.Pell., comb. et stat. nov.

urn:lsid:ipni.org:names:77166531-1

Figs 6S , 11 , 13

Tradescantia sect. Setcreasea (K.Schum. & Sydow) D.R.Hunt, Kew Bull. 30(3): 448. 1975.
Neotreleasea Rose, Contr. U.S. Natl. Herb. 8: 5. 1903, nom. superfluous.
Setcreasea K.Schum. & Sydow, Just’s Bot. Jahresber. 27(1): 452. 1901.
Treleasea Rose, Contr. U.S. Natl. Herb. 5: 207. 1899, nom. illeg., non Treleasia Speg., Revista Fac. Agron. Univ. Nac. La Plata 2: 235. 1896. Type species. Tradescantia leiandra var. brevifolia Torr. [≡ T. brevifolia (Torr.) Rose]
Tradescantia sect. Separotheca (Waterf.) D.R.Hunt, Kew Bull. 30(3): 454. 1975.
Separotheca Waterf., Rhodora 61: 138. 1959, **Syn. nov.** Type species. Zebrina pumila Greene (≡ T. pygmaea D.R.Hunt).
Tradescantia sect. Tradescantia ser. Sillamontanae D.R.Hunt, Kew Bull. 35(2): 440. 1980, **Syn. nov.** Type species. Tradescantia sillamontana Matuda
Tradescantia sect. Tradescantia ser. Orchidophyllae D.R.Hunt, Kew Bull. 35(2): 441. 1980, **Syn. nov.** Type species. Tradescantia orchidophylla Rose & Hemsl.

#### Description.

*Herbs* geophytes, base definite, perennial, succulent, terrestrial or rupicolous. *Roots* thick, tuberous. *Stems* erect, sometimes prostrate with ascending apex, succulent, little branched to densely branched, rarely unbranched, rooting at the basal nodes, sometimes rooting at the distal ones when they touch the substrate. *Leaves* sessile; spirally-alternate, rarely distichously-alternate, evenly distributed along the stem or congested at the apex of the stems; sheaths closed; blades falcate and/or complicate, base symmetric, midvein conspicuous to inconspicuous, adaxially impressed, abaxially prominent, rounded, secondary veins conspicuous or inconspicuous. *Synflorescences* terminal in the distal portion of the stems, composed of a solitary main florescence. *Inflorescences (main florescences)* consisting of a pedunculate double-cincinni fused back to back; inflorescence bract hyaline, tubular, inconspicuous; peduncle bracts absent; supernumerary bracts absent; cincinni bracts leaf-like, unequal to each other, saccate, conduplicate, free, overlapping each other; bracteoles expanded, imbricate or completely involving the cincinnus, linear-triangular to triangular or flabellate, hyaline. *Flowers* bisexual, actinomorphic, tubular, floral tube infundibuliform to hypocrateriform or campanulate; pedicel gibbous at apex, straight at anthesis and pre-anthesis, deflexed at post-anthesis; sepals equal, free, membranous, elliptic to broadly elliptic, not dorsally keeled, apex acute; petals sessile or clawed, equal, free to conate, blade elliptic to ovate to broadly ovate or rhomboid to broadly obovoid to obovoid, flat, base cuneate to obtuse, margin entire, apex acute to obtuse; stamens 6, arranged in two series, equal, filaments epipetalous, straight at anthesis and post-anthesis, glabrous to medially sparsely bearded with moniliform hairs, when present hairs shorter than the stamens, variously colored, anthers with connective quadrangular to rectangular, rarely rhomboid, yellow, anther sacs C-shaped, rarely ellipsoid, yellow, pollen yellow; ovary glabrous or pubescent, locules 2-ovulate, style straight at anthesis and post-anthesis, variously colored, cylindrical at base, cylindrical to obconical at the apex, stigma capitate to trilobate, pistil the same length as the stamens. *Capsules* subglobose to globose, light to medium brown when mature, glabrous or pubescent, loculicidal, 3-valved, sometimes apiculate due to persistent style base. *Seeds* exarillate, 1–2 per locule, ellipsoid to narrowly trigonal, ventrally flattened, not cleft towards the embryotega, testa scrobiculate to rugose, with ridges radiating from the embryotega, embryotega dorsal, conspicuous, with a prominent apicule.

#### Habitat, distribution and ecology.

Tradescantia subg. Setcreasea is restricted to southern USA and Mexico (Fig. 11). Its species are generally related to rocky outcrops and open dry areas. This is reflected in its species with tuberous roots and succulent vegetative organs.

#### Included species.

This subgenus is composed by 10 species: *Tradescantia brevifolia* (Torr.) Rose, *T. buckleyi* (I.M.Johnst.) D.R.Hunt, *T. hirta* D.R.Hunt, *T. leiandra* Torr., *T. mirandae* Matuda, *T. orchidophylla* Rose & Hemsl., *T. pallida* (Rose) D.R.Hunt, *T. pygmaea* D.R.Hunt, *T. rozynskii* Matuda, and *T. sillamontana* Matuda.

#### Comments.

**Figure 13.**
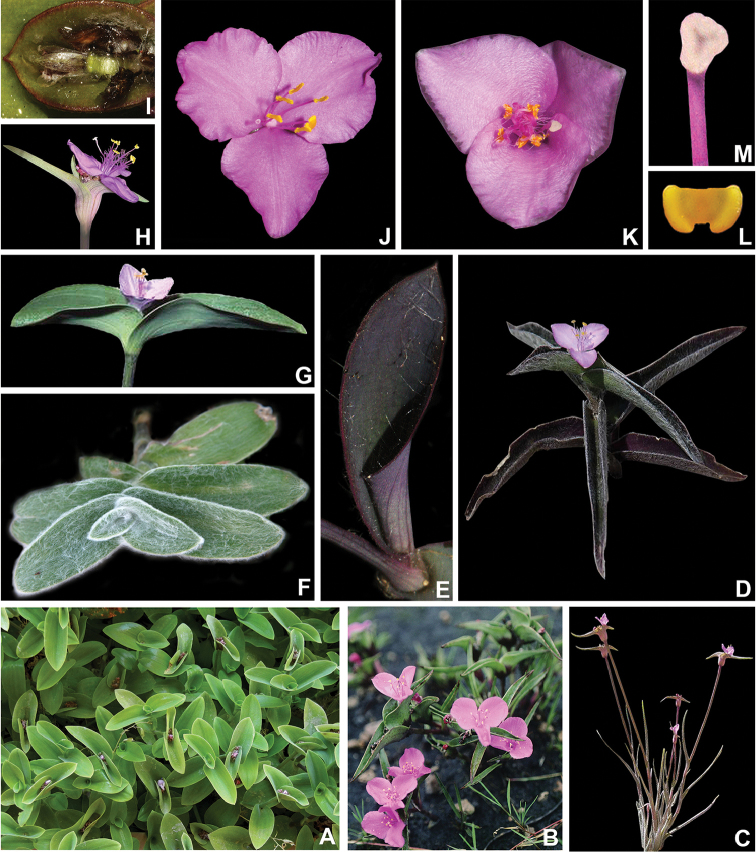
Tradescantia subg. Setcreasea (K.Schum. & Sydow) M.Pell. **A–D** habit: **A** prostrate habit with ascending apex to *T. buckleyi* (I.M.Johnst.) D.R.Hunt **B** the dwarf habit of *T. pygmaea* D.R.Hunt **C** erect habit of *T. hirta* D.R.Hunt **D** habit of *T. rozynskii* Matuda, showing the spirally-alternate and strongly complicate leaves **E–F** leaves: **E** young leaf of *T. pallida* (Rose) D.R.Hunt cv. *Purpurea*, showing the glabrous leaves with lanate hairs at the margin **F** branch of *T. sillamontana* Matuda, showing the distichously-alternate leaves, densely covered by lanate hairs **G–H** inflorescence: **G** main florescence of *T. brevifolia* **H** inflorescence of *T. hirta* **I** post-anthesis flower of *T. sillamontana*, showing the hyaline sepals. **J–K** flowers: **J** front view of a flower of *T. sillamonata* **K** front view of a flower of *T. pallida* **L** anther of *T. pallida*, showing the quadrangular connective and C-shaped anther sacs **M** style of *T. pallida*, showing the trilobate stigma. **A** by J.M. Jenkins, **B** by M. Egger, **C** by J.-P. Piquet, **D** by J. Vích, **E, I–M** by M.O.O. Pellegrini, **F** by D. Stang, **G** by K. Yatskievych, and **H** by O. Peri.

Tradescantia subg. Setcreasea comprises succulent plants with complicate leaves (Fig. 13A–F), tubular flowers (generally sympetalous and epipetalous; Fig13D, G–H, J–K) and filaments that range from glabrous to sparsely barbate with short moniliform hairs (Fig. 13H, J, K). This group was thoroughly studied and almost completely monographed by Hunt (1975), with only four of its currently accepted species not included in the key. Its morphology is considerably homogeneous, with species related with the commonly cultivated *T. pallida* forming a species complex (Fig. 13A, E, G, K). Tradescantia sect. Tradescantia ser. Sillamontanae was differentiated from T. sect. Setcreasea by Hunt (1980) by the free petals and stamens (Fig. 13J), and densely lanate leaves (Fig. 13D, F); while T. sect. Tradescantia ser. Orchidophyllae was differentiated by its free petals and stamens, and generally rotund leaves congested in a rosette. Nevertheless, these two groups share all the diagnostic features of T. subg. Setcreasea (i.e. tubular flowers, pedicel the same length as the floral buds, hyaline sepals, fused and clawed petals, and epipetalous stamens), and there seems to be no good reason for treating them as separate groups inside T. subg. Setcreasea. Furthermore, in the majority rule topology (Fig. 4A), these species are nested deep within T. subg. Setcreasea and there is no way to recognize them as separate groups, without creating other non-monophyletic groups inside the subgenus. The peculiar-looking *T. hirta* (Fig. 13C, H), was originally included by Hunt (1975) in his T. sect. Setcreasea, and is morphologically very similar to *T. mirandae*, differing primarily in leaf shape and androecium morphology. Furthermore, *T. rozynskii* (Fig. 13D) and *T. sillamontana* (Fig. 13F, I, J) can only be differentiated from the *T. pallida* species complex due to their lanate indumentum covering the entire leaf-blade, and lack of clawed petals. Aside from that, these plants are morphologically very similar (see Fig. 13). *Tradescantia orchidophylla* is the morphologically most discrepant species in the subgenus, due to its wide leaf-blades and very long pedicels. Nonetheless, this morphology could be easily explained as a return to understory environments. Despite being placed by Hunt (1975) in a separate section, *T. pygmaea* is undeniably similar to the species from the *T. pallida* species complex. Besides the obvious stature difference (hence the species’ name; Fig. 13B), and the thicker tuberous roots, the only marking morphological difference between it and the species from the *T. pallida* complex is the shape of the connectives and anthers sacs that are sagittate and elliptic, similar to the ones of *T. mirandae*.

### 2.5. Tradescantia L. subg. Tradescantia

Taxon classificationPlantaeCommelinalesCommelinaceae

Figs 6T , 11 , 14

Tradescantia L. sect. Tradescantia *sensu*Hunt (1980), *pro parte*
Tradescantia sect. Tradescantia ser. Virginianae D.R.Hunt, Kew Bull. 35(2): 440. 1980, **Syn. nov.** Type species. Tradescantia virginiana L.
Tradescantia sect. Tradescantia ser. Tuberosae D.R.Hunt, Kew Bull. 35(2): 441. 1980, **Syn. nov.** Type species. Tradescantia tuberosa Greene (≡ T. pinetorum Greene)
Ephemerum Mill., Gard. Dict. Abr., ed. 4.: 462. 1754, **Syn. nov.** Type species. Ephemerum virginianum (L.) Mill. (≡ Tradescantia virginiana L.).

#### Type species.

*Tradescantia virginiana* L.

#### Description.

*Herbs* geophytes, base definite, perennial, sometimes annual, succulent, terrestrial or rupicolous. *Roots* thick, tuberous. *Stems* erect, sometimes prostrate with apex, succulent, unbranched to little branched to branched only at base, rooting at the basal nodes, rarely rooting at the distal ones when they touch the substrate. *Leaves* sessile; spirally-alternate, evenly distributed along the stem, sometimes congested at the apex of the stems; sheaths closed, commonly splitting open at maturity; blades falcate and/or complicate, base symmetric, midvein conspicuous, adaxially impressed, abaxially prominent, rounded, secondary veins conspicuous. *Synflorescences* terminal in the distal portion of the stems, composed of a solitary main florescence. *Inflorescences (main florescences)* consisting of a pedunculate double-cincinni fused back to back; inflorescence bract hyaline, tubular, inconspicuous; peduncle bracts absent; supernumerary bracts absent; cincinni bracts leaf-like, unequal to each other, saccate or not, conduplicate, free, overlapping each other; bracteoles expanded, imbricate, linear-triangular to triangular, hyaline. *Flowers* bisexual, actinomorphic, flat; pedicel non-gibbous at apex, straight at anthesis and pre-anthesis, deflexed at post-anthesis; sepals equal, free, membranous, elliptic to broadly elliptic, not dorsally keeled, apex acute; petals sessile, equal, free, blade ovate to broadly ovate or rhomboid to broadly obovoid to obovoid, flat or plicate, base cuneate to obtuse, margin entire, apex acute to obtuse; stamens 6, arranged in two series, equal, filaments free, straight at anthesis and post-anthesis, to medially densely bearded with moniliform hairs, hairs shorter than the stamens, variously colored, anthers with connective quadrangular to rectangular, yellow, anther sacs C-shaped, yellow, pollen yellow; ovary glabrous, locules 2-ovulate, style straight at anthesis and post-anthesis, variously colored, cylindrical at base, obconical at the apex, stigma capitate to trilobate, pistil the same length as the stamens. *Capsules* subglobose to globose, light to medium brown when mature, glabrous, loculicidal, 3-valved, sometimes apiculate due to persistent style base. *Seeds* exarillate, 1–2 per locule, ellipsoid to narrowly trigonal, ventrally flattened, not cleft towards the embryotega, testa scrobiculate to rugose, with ridges radiating from the embryotega, embryotega dorsal, conspicuous, with a prominent apicule.

#### Habitat, distribution and ecology.

Tradescantia subg. Tradescantia is restricted to Canada, USA and Mexico, but considerably more diverse in the USA (Fig. 11). Its species generally grow in open grasslands, pine forests or open rocky areas.

#### Included species.

The subgenus includes ca. 30 species, namely: *Tradescantia bracteata* Small *ex* Britton, *T. cirrifera* Mart., *T. edwardsiana* Tharp, *T. ernestiana* E.S.Anderson & Woodson, *T. gigantea* Rose, *T. gypsophila* B.L.Turner, *T. hirsuticaulis* Small, *T. hirsutiflora* Bush, *T. humilis* Rose, *T. longipes* E.S.Anderson & Woodson, *T. monosperma* Brandegee, *T. occidentalis* (Britton) Smyth, *T. ohiensis* Raf., *T. ozarkana* E.S.Anderson & Woodson, *T. pedicellata* Celarier, *T. pinetorum* Greene, *T. reverchonii* Bush, *T. roseolens* Small, *T. stenophylla* Brandegee, *T. subacaulis* Bush, *T. subaspera* Ker Gawl., *T. subtilis* Matuda (= *T. maysillesii* Matuda), *T. tharpii* E.S.Anderson & Woodson, *T. virginiana* L., and *T. wrightii* Rose & Bush. The species native to the United States have been thoroughly revised by Anderson and Woodson Jr. (1935). However, as stated by the authors, the species concentrated in Mexico are still in need of taxonomic revision, and might reveal taxonomic novelties.

#### Comments.

**Figure 14.**
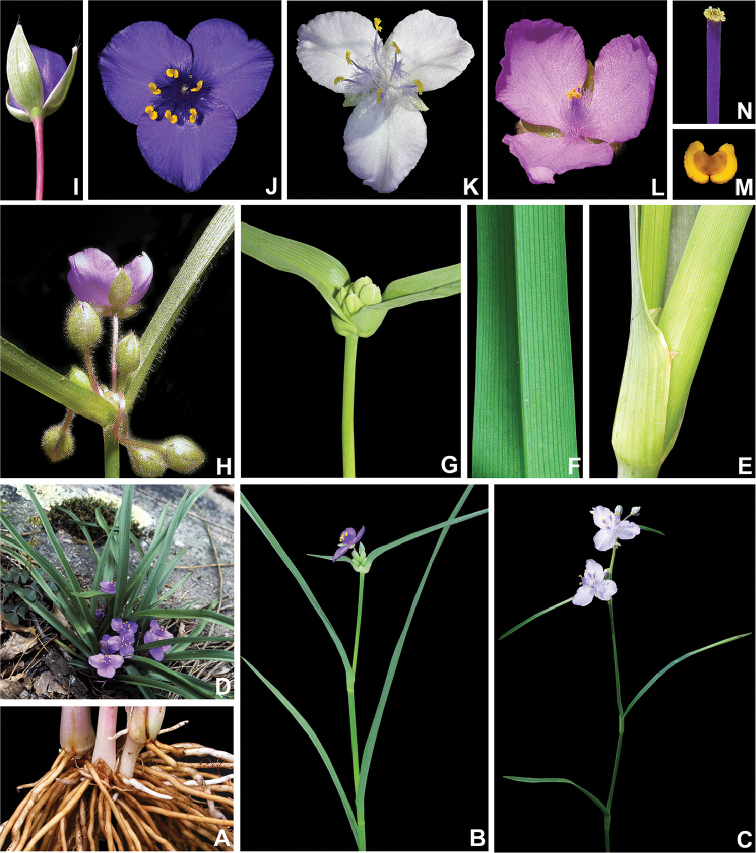
Tradescantia L. subg. Tradescantia. **A** tuberous roots of *T. ohiensis* Raf. **B–D** habit: **B** erect robust habit of *T. ohiensis* **C** erect delicate habit of *T. pinetorum* Greene **D** rosette habit of *T. longipes* E.S.Anderson & Woodson. **E**, leaf-sheath split at maturity of *T. ohiensis* **F** detail of the leaf-blade of *T. ohiensis*, showing the conspicuous secondary veins **G–H** inflorescence: **G** inflorescence of *T. ohiensis*, showing the saccate cincinni bracts **H** inflorescence of *T. virginiana* L., showing the non-saccate cincinni bracts and densely pubescent bracts, pedicels and sepals **I** floral bud of *T. ohiensis*, showing the non-gibbous pedicel apex **J–L** flowers: **J** front view of a flower of *T. ohiensis* **K** front view of a flower of *T. pinetorum* **L** oblique view of a flower of *T. virginiana* **M** anther of *T. ohiensis*, showing the quadrangular and slightly curved connective, and the C-shaped anther sacs. **N**, style of *T. ohiensis*, showing the capitate stigma. **A–B, E–F, H–I, L** by G. Davidse, **C, K** by R.W. Van Devender, **D** by B. Nellums, and **G, J, M–N** by M.O.O. Pellegrini.

Tradescantia subg. Tradescantia can be easily differentiated from the remaining subgenera by its grass-like appearance (Fig. 14 B–D, F), leaf-sheaths split open at maturity (Fig. 14E), linear leaf-blades (Fig. 14 B–D, F), pedicels apically non-gibbous (Fig. 14H, I), filaments densely bearded with moniliform hairs (Fig. 14J–L), and stigmatic papillae restricted to the margins of the stigma (i.e. leaving the stylar canal evident; Fig. 14N)). As aforementioned, this subgenus contains the biggest flowers of *Tradescantia*, being commonly cultivated all around the world. The group’s taxonomy, ontogeny, cytology, reproductive system, and hybridization were heavily studied by Anderson and Diehl (1932), Anderson and Woodson Jr. (1935), Anderson (1936a, 1936b), Anderson and Hubricht (1938), Anderson and Sax (1936), Celarier (1955), Darlington (1929, 1937), Hubricht and Anderson (1941), King (1933), Riley (1937), Sax and Anderson (1933), Sax and Edmonds (1933), Sax and Humphrey (1934), and Showalter (1938). Nonetheless, the group’s taxonomy remains challenging due to the high frequency of hybridization in nature (see references above) and the recent origin of the group, illustrated by the extremely short branches and poorly-resolved internal relationships recovered by Hertweck and Pires (2014).

Hunt (1980) proposed the distinction of T. subg. Tradescantia ser. Virginianae and T. subg. Tradescantia ser. Tuberosae, based solely on the degree of thickening of the roots. Nonetheless, T. subg. Tradescantia ser. Virginianae is recovered as non-monophyletic in the present analysis, due to the two species from T. subg. Tradescantia ser. Tuberosae sampled as nested within it. All species in Core *Tradescantia* (including T. subg. Tradescantia) possess somewhat tuberous roots, and the degree of thickening seems to be of little phylogenetic relevance. Thus, I chose not to recognized any sections or series in T. subg. Tradescantia. Furthermore, due to the relatively small size of *Tradescantia*, and reduced number of species in each subgenus, the recognition of sections and series in the present infrageneric classification seems unnecessary.

## Conclusions

One of the main paradigms of modern phylogenetic systematics is the proposal of new classification systems that reflect the evolutionary history of the studied group, and being at the same time easy to use (Simpson 2006). It means that classification systems should be based on molecular phylogenetic studies, but also present morphological synapomorphies to easily characterize the proposed taxonomic ranks. On the other hand, it is widely believed that morphologically based phylogenies are less reliable than molecular based ones, due to the high degree of homoplasy expected to exist in morphological datasets. Furthermore, most modern taxonomists expect high degrees of incongruence between morphological and molecular datasets (Evans and Faden 1998). Nonetheless, the present study, along with other cases in two other families in the order Commelinales (Haemodoraceae – Simpson 1990, Hopper et al. 2009, Aerne-Hains and Simpson 2017; Pontederiaceae – Eckenwalder and Barrett 1986, Graham and Barrett 1995, Kohn et al. 1996, Barret and Graham 1997, Graham et al. 1998, Simpson and Burton 2006, Ness et al. 2011), yield phylogenetically congruent results. Thus, morphological phylogenies reconstructed with properly coded matrixes, and also including different data types (e.g. macromorphology, micromorphology, cytology, phytochemistry, etc.), can indeed recover evolutionary hypotheses congruent with molecular-based phylogenies. In the past years, morphology-based phylogenies have become much less common than molecular-based phylogenies (Saraiva et al. 2015). Nonetheless, morphological characters can greatly improve the resolution of phylogenetic hypothesis in plant groups (Aagesen and Sanso 2003; Evans et al. 2003; Wade et al. 2003; Barfuss et al. 2005, 2015; Faria 2006; Rex et al. 2009; Saraiva et al. 2015; Gomes-da-Silva and Souza-Chies 2017). Furthermore, without the inclusion of morphological characters in a phylogenetic analysis, there is no way to obtain morphological synapomorphies to support the recovered relationships and any proposed new classification (Lipscomb et al. 2003; Wiens 2004; Assis and Rieppel 2011). Thus, it is desirable for taxonomists and systematists to embrace once again morphological characters and phylogenies so they can reflourish as effective low-cost tools to better understand phylogenetic relationships of plant taxa. This study is the first of a series of publications dealing with the systematics and generic limits in Commelinaceae, uniting morphology, little used morphological traits and molecular evidence. Future studies focusing on the remaining generic problems of subtribe Tradescantiinae (i.e. the *Callisia*/*Tripogandra* generic complex) are currently in preparation, to conclude the herein presented systematic overview of the subtribe and carry on the studies of Dr. David R. Hunt on Neotropical Commelinaceae.

## Supplementary Material

XML Treatment for Tradescantiinae

XML Treatment for Thyrsantheminae

XML Treatment for Elasis

XML Treatment for Elasis guatemalensis

XML Treatment for Tradescantia

XML Treatment for Tradescantia subg. Austrotradescantia

XML Treatment for Tradescantia subg. Campelia

XML Treatment for Tradescantia subg. Mandonia

XML Treatment for Tradescantia subg. Setcreasea

XML Treatment for Tradescantia L. subg. Tradescantia

## Supplementary materials

Supplementary material 1 List of morphological characters and coding Data type: phylogenetic data This dataset is made available under the Open Database License (http://opendatacommons.org/licenses/odbl/1.0/). The Open Database License (ODbL) is a license agreement intended to allow users to freely share, modify, and use this Dataset while maintaining this same freedom for others, provided that the original source and author(s) are credited.Author: Marco O. O. Pellegrini

Supplementary material 2 Matrix with the 60 terminals and the 114 characters Data type: phylogenetic data This dataset is made available under the Open Database License (http://opendatacommons.org/licenses/odbl/1.0/). The Open Database License (ODbL) is a license agreement intended to allow users to freely share, modify, and use this Dataset while maintaining this same freedom for others, provided that the original source and author(s) are credited.Author: Marco O. O. Pellegrini
