# Supplementary material for: Morphological phylogeny of Tradescantia L. (Commelinaceae) sheds light on a new infrageneric classification for the genus and novelties on the systematics of subtribe Tradescantiinae
Source: PhytoKeys. 2017 Oct 26;(89):11–72. doi: 10.3897/phytokeys.89.20388 (PMC5672149; doi:10.3897/phytokeys.89.20388)
Supplement: Supplementary material 1 — List of morphological characters and coding [file phytokeys-89-011-s001.doc]

**Supplement 1.** List of morphological characters and coding. The terminology for indumentum (Characters 12–17, 58, 66–71 & 85) and shapes (Characters 18–20, 40, 43, 47, 54–55, 61–63, 72–82 & 89–91) follows Radford et al. (1974); inflorescence terminology (Characters 24–45) follows Weberling (1965, 1989) and Panigo et al. (2011); stigmatic micromorphology terminology (Characters 92–94) follows Owens and Kimmins (1981) and Owens et al. (1984); seed terminology (Characters 95–102) follows Faden (1991); phytochemical terminology (Characters 110–114) follows (Martínez and Martínez 1993); cytology terminology (Characters 106–109) follows (Jones and Jopling 1972; Martínez and Ginzo 1985); pollen terminology (Characters 83–84) follows (Poole and Hunt 1980); anatomical terminology (Characters 103–105) follows Tomlinson (1966, 1969); and general macromorphological terminology follows Pellegrini (2015; remaining characters).

1. Roots, shape: non-tuberous (0); tuberous (1)
2. Stem, base: definite (0); indefinite (1)
3. Stem, posture: prostrate with decumbent or ascendant apex (0); erect (1)
4. Stem, consistency: herbaceous (0); crass to fibrous (1)
5. Stem, branching: unbranched to branched only at base (0); densely branched (1)
6. Leaves, phyllotaxy: distichously-alternate (0); distichously-spiral (1)
7. Leaves, distribution on the stem: evenly distributed (0); congested at the apex (1)
8. Leaves, insertion: sessile (0); subpetiolate (1)
9. Leaves, blade, shape: oblong or elliptic or lanceolate (0); linear (1); ovate (2); obovate or rotund (3)
10. Leaves, blade, consistency: chartaceous (0); membranous (1); crass (2)
11. Leaves, blade, architecture: flat (0); falcate or conduplicate (1)
12. Leaves, blade, pubescence, adaxial side: absent (0); present (1)
13. Leaves, blade, pubescence, adaxial side, type: velutine (0); pilose (1); hispid to hirsute (2) lanate (3); glandular-pubescent (4); scabrid (5)
14. Leaves, blade, pubescence, adaxial side, color: hyaline (0); golden to brown (1)
15. Leaves, blade, pubescence, abaxial side: absent (0); present (1)
16. Leaves, blade, pubescence, abaxial side, type: velutine (0); pilose (1); hispid to hirsute (2) lanate (3); glandular-pubescent (4); scabrid (5)
17. Leaves, blade, pubescence, abaxial side, color: hyaline (0); golden to brown (1)
18. Leaves, blade, base, shape: cuneate (0); truncate to amplexicaulous (1); obtuse to cordate (2)
19. Leaves, blade, base, symmetry: symmetric (0); asymmetric (1)
20. Leaves, blade, apex, shape: round to obtuse (0); acute (1); acuminate (2)
21. Leaves, blade, adaxial side, secondary veins, impression: conspicuous (0); inconspicuous (1)
22. Leaves, blade, adaxial side, longitudinal stripes: absent (0); present (1)
23. Leaves, blade, adaxial side, color: green (0); vinaceous to brown (1)
24. Synflorescence, structure: exclusively terminal or restricted to the apex of the stem (0); mainly axillary to thyrse-like (1)
25. Inflorescence, basal bract, shape: reduced and tubular (0); leaf-like (1)
26. Inflorescence, structure: solitary main florescence (0); main florescence plus 1–several coflorescences (1)
27. Inflorescence, emergence: not perforating the leaf-sheaths (0); perforating the leaf-sheaths (1)
28. Inflorescence, peduncle, development: sessile (0); pedunculate (1)
29. Inflorescence, peduncle, accessory bracts: absent (0); present (1)
30. Cincinni, number per main florescence: two (0); variable (1)
31. Cincinni, arrangement: subopposite (0); verticillate (1); fasciculate/side by side (2)
32. Cincinni, peduncle, development: sessile (0); shortly-pedunculate (1); long-pedunculate (2)
33. Cincinni, peduncle, posture: erect (0); geniculate (1)
34. Cincinni, elongation: contracted (0); elongate (1)
35. Cincinni, fusion: free (0); fused back to back (1)
36. Cincinni, number per cincinni bract: one (0); more than one (1)
37. Cincinni, supernumerary bracts: absent (0); present (1)
38. Cincinni, bracts, development: reduced to hyaline crest or bracteose (0); frondose (1)
39. Cincinni, bracts, frondose, shape: leaf-like (0); spathaceous (1)
40. Cincinni, bracts, shape, among themselves: equal (0); unequal (1)
41. Cincinni, bracts, architecture: flat (0); falcate or conduplicate (1)
42. Cincinni, bracts, base, fusion: free (0); fused (1)
43. Cincinni, bracts, base, shape: non-saccate (0); saccate (1)
44. Cincinni, bracts, base, overlapping: not overlapping (0); overlapping (1)
45. Bracteoles, development: reduced (0); expanded (1)
46. Flower, sex: bisexual (0); sometimes unisexual, either pistillate or staminate (1)
47. Flower, perianth, shape: flat/rotate (0); tubular (1)
48. Flower, display angle: 0° (0); with a 60° torsion (1)
49. Flower, pedicel, apex: thin (0); gibbous (1)
50. Flower, pedicel, relative length: sessile to subsessile (0); ca. the same length as the floral bud (1); longer than the floral bud (2)
51. Flower, pedicel, posture, at anthesis and pre-anthesis: straight (0); geniculate (1)
52. Flower, pedicel, posture, at post-anthesis: straight (0); deflexed (1); reflexed (2); oblique (3)
53. Sepals, fusion: free (0); fused at least at one point (1)
54. Sepals, shape among themselves: equal to subequal (0); one sepal strongly different from the other two (1)
55. Sepals, shape: elliptic or broadly elliptic or obovate (0); ovate (1); linear to lanceolate (2)
56. Sepals, keel: absent (0); present in all sepals (1); present in only one sepal (2)
57. Sepals, consistency: membranous (0); chartaceous (1); fleshy (2)
58. Sepals, pubescence: glabrous (0); pilose or velutine or lanate or setose (1); hispid to hirsute (2); glandular-pubescent (3)
59. Sepals, color: hyaline or white (0); mainly green (1); vinaceous or red or pink (2)
60. Petals, fusion: free (0); fused at least at one point (1)
61. Petals, shape: elliptic or ovate (0); obovate (1); oblong to linear (2); rhomboid or rotund (3)
62. Petals, claw: absent (0); present (1)
63. Petals, shape among themselves: equal to subequal (0); one sepal strongly different from the other two (1)
64. Petals, color: blue (0); white (1); pink or lilac to purple (2); green or hyaline (3)
65. Androecium, stamens, number (including staminodes): six (0); three to one (1)
66. Androecium, stamens, outer whorl (antesepalous), pubescence, density: absent to sparse (0); dense (1)
67. Androecium, stamens, outer whorl (antesepalous), pubescence, position: basal (0); until mid-length (1); terminal (2)
68. Androecium, stamens, outer whorl (antesepalous), pubescence, length: short (0); half the length of the filaments (1); ca. the same length as the filaments (2)
69. Androecium, stamens, inner whorl (antepetalous), pubescence, density: absent to sparse (0); dense (1)
70. Androecium, stamens, inner whorl (antepetalous), pubescence, position: basal (0); until mid-length (1); terminal (2)
71. Androecium, stamens, inner whorl (antepetalous), pubescence, length: short (0); half the length of the filaments (1); ca. the same length as the filaments (2)
72. Androecium, stamens, whorls, relative size: equal (0); shorter outer whorl (longer inner whorl) (1); different sizes in the same whorl (non-comparable whorls) (2)
73. Androecium, stamens, filaments, curvature, at anthesis: sigmoid (0); straight (1)
74. Androecium, stamens, filaments, curvature, at post-anthesis: straight (0); sigmoid (1); spirally-coiled (2)
75. Androecium, fusion to the corolla: free (0); epipetalous (1)
76. Androecium, symmetry: actinomorphic (0); zygomorphic (1)
77. Androecium, stamens, outer whorl (antesepalous), connective, development: inconspicuous (0); expanded (1)
78. Androecium, stamens, outer whorl (antesepalous), connective, shape: cordate or sagittate or linearly-tapered (0); quadrangular to slightly curved (1); flabellate (2); rhomboid (3)
79. Androecium, stamens, inner whorl (antepetalous), connective, development: inconspicuous (0); expanded (1)
80. Androecium, stamens, inner whorl (antepetalous), connective, shape: cordate or sagittate or linearly-tapered (0); quadrangular to slightly curved (1); flabellate (2); rhomboid (3)
81. Androecium, stamens, outer whorl (antesepalous), anther sacs, shape: round (0); elliptic (1); reniform to C-shaped (2); linear (3)
82. Androecium, stamens, inner whorl (antepetalous), anther sacs, shape: round (0); elliptic (1); reniform to C-shaped (2); linear (3)
83. Androecium, stamens, pollen, color *in vivo*: white or the same color as the petals (0); yellow (1)
84. Androecium, stamens, pollen, tectum, ornamentation: espinulose (0); reticulate (1); insulate cerebroid (2); areolate (3); insulate domed (4)
85. Gynoecium, ovary, pubescence: glabrous (0); eglandular (1); glandular-pubescent (2)
86. Gynoecium, pistil, relative length: shorter than the stamens (0); ca. the same length as the stamens (1); longer than the stamens (2); ½ times longer than the stamens (3)
87. Gynoecium, style, curvature, at anthesis: straight (0); sigmoid (1)
88. Gynoecium, style, curvature, at anthesis: straight (0); sigmoid (1); spirally-coiled (2)
89. Gynoecium, style, base, shape: cylindrical (0); obconic (1)
90. Gynoecium, style, apex, shape: cylindrical (0); conic (1); obconic (2)
91. Gynoecium, stigma, shape: capitate or subtrilobate to trilobate (0); punctate (1); truncate to capitulate (2); penicilliform (3)
92. Gynoecium, stigma, papillae, type: A (0); B (1); C (2); D (3); E (4)
93. Gynoecium, stigma, papillae, length: longer than 1μm (0); equal or shorter than 1μm (1); equal or shorter than 0.5μm (2)
94. Gynoecium, stigma, papillae, distribution in the stigma: evenly distributed (0); restricted to the margins (1)
95. Seeds, outline, shape: elliptic to oblong (0); triangular to round triangular or tetrahedral (1); quadrangular (2)
96. Seeds, sulcus towards the embryotega: absent (0); present (1)
97. Seeds, ventral side, shape: flat (0); ridged (1); depressed (2)
98. Seeds, embryotega, position: dorsal (0); semilateral (1)
99. Seeds, embryotega, apicule: inconspicuous (0); conspicuous (1)
100. Seeds, testa, ornamentation: smooth (0); reticulate to foveolate (1); costate (2); rugose to scrobiculate (3)
101. Seeds, hilum: punctate to elliptic (0); linear (1); curved to C-shaped (2)
102. Seeds, hilum, relative length: shorter than ½ the length of the seed (0); ca. ½ the length of the seed (1); longer than ½ the length of the seed (2)
103. Anatomy, leaf epidermis, silica crystals, in specialized cells, presence: absent (0); present (1)
104. Anatomy, leaf epidermis, silica crystals, in specialized cells, cell walls: thin (0); thickened (1)
105. Anatomy, mesophyll, longitudinal bundles: diffuse (0); with fibrous extensions (1)
106. Chromosomes, number: n= 4–5 (0); n= 6 (1); n= 7–8 or ranging between 5–6–10–11 due to Robertsonian Translocations (2); n= 8 (3); n= 10 (4)
107. Chromosomes, size: equal or shorter than 5µm (0); longer than 5µm and shorter than 10µm (1); equal or longer than 10µm (2)
108. Chromosomes, karyotype, homogeneity: unimodal (0); bimodal (1)
109. Chromosomes, complements, symmetry: symmetric (0); asymmetric (1)
110. Phytochemistry, hidroxiluteolin: absent (0); present (1)
111. Phytochemistry, C-glycosides: absent (0); 1 C-glycosides (1); 2–8 C-glycosides (2)
112. Phytochemistry, flavonoids, quercetin: absent (0); present (1)
113. Phytochemistry, flavones, 6-hidroxiluteolin: absent (0); present (1)
114. Phytochemistry, sulphated phenolic acids: absent (0); present (1)
