# Supplementary material for: Morphological phylogeny of Tradescantia L. (Commelinaceae) sheds light on a new infrageneric classification for the genus and novelties on the systematics of subtribe Tradescantiinae
Source: PhytoKeys. 2017 Oct 26;(89):11–72. doi: 10.3897/phytokeys.89.20388 (PMC5672149; doi:10.3897/phytokeys.89.20388)
Supplement: Supplementary material 2 — Matrix with the 60 terminals and the 114 characters [file phytokeys-89-011-s002.doc]

**Supplement 2.** Matrix with the 60 terminals and the first 29 characters. The characters that were not coded due to lack of data of the analyzed specimens and/or from literatures sources are coded as “?”; characters that did not apply were coded as “-”; and the polymorphic characters were coded with a “/” between each state it presented.

| **Taxon** | **1** | **2** | **3** | **4** | **5** | **6** | **7** | **8** | **9** | **10** | **11** | **12** | **13** | **14** | **15** | **16** | **17** | **18** | **19** | **20** | **21** | **22** | **23** | **24** | **25** | **26** | **27** | **28** | **29** |
| --- | --- | --- | --- | --- | --- | --- | --- | --- | --- | --- | --- | --- | --- | --- | --- | --- | --- | --- | --- | --- | --- | --- | --- | --- | --- | --- | --- | --- | --- |
| ***Tin. erecta*** | 0 | 0 | 1 | 1 | 0 | 1 | 1 | 1 | 0 | 1 | 0 | 0/1 | 2 | 0 | 1 | 2 | 0 | 0 | 0 | 2 | 0 | 0 | 0 | 0 | 1 | 0 | 0 | 1 | 0 |
| ***Tin. sprucei*** | 0 | 0 | 1 | 1 | 0 | 1 | 1 | 1 | 0 | 1 | 0 | 0/1 | 2 | - | 0/1 | 2 | - | 0 | 0 | 1/2 | 0 | 0 | 0 | 0 | 1 | 0 | 0 | 1 | 0 |
| ***Trad. tenella*** | 0 | 0 | 1 | 1 | 1 | 0 | 0 | 1 | 0/2 | 1 | 0 | 0/1 | 2 | 0 | 0/1 | 2 | 0 | 0/2 | 1 | 1/2 | 0 | 0/1 | 0/1 | 0 | 0 | 0 | 0 | 1 | 0 |
| ***Trad. cerinthoides*** | 0 | 0 | 1 | 1 | 0 | 0/1 | 0 | 0 | 0/2/3 | 2 | 1 | 0/1 | 1/2 | 0/1 | 0/1 | 1/2 | 0/1 | 1/2 | 1 | 0/1 | 0/1 | 0/1 | 0/1 | 0 | 0 | 0 | 0 | 1 | 0 |
| ***Trad. crassula*** | 0 | 0 | 1 | 1 | 0 | 0/1 | 0 | 0 | 0/2/3 | 2 | 1 | 0 | - | - | 0 | - | - | 1/2 | 1 | 0/1 | 0/1 | 0 | 0 | 0 | 0 | 0 | 0 | 1 | 0 |
| ***Trad. fluminensis*** | 0 | 1 | 0 | 0 | 1 | 0 | 0 | 1 | 0/2 | 1 | 0 | 0 | - | - | 0 | - | - | 0/2 | 1 | 1 | 0 | 0 | 0 | 0 | 0 | 0 | 0 | 1 | 0 |
| ***Trad. mundula*** | 0 | 1 | 0 | 0 | 1 | 0 | 0 | 1 | 0/2 | 1 | 0 | 0/1 | 5 | 0 | 1 | 5 | 0 | 0/2 | 1 | 1/2 | 0 | 0 | 0/1 | 0 | 0 | 0 | 0 | 1 | 0 |
| ***Trad. cymbispatha*** | 0 | 1 | 0 | 0 | 1 | 0 | 0 | 0 | 2 | 2 | 0 | 1 | 5 | 0 | 1 | 5 | 0 | 2 | 1 | 1/2 | 1 | 0 | 1 | 0 | 0 | 0 | 0 | 1 | 0 |
| ***Trad. umbraculifera*** | 0 | 1 | 0 | 0 | 1 | 0 | 0 | 0 | 0/1 | 0 | 0 | 0/1 | 1 | 0 | 0/1 | 1 | 0 | 0/2 | 1 | 2 | 0 | 0 | 0/1 | 0 | 0 | 0 | 0 | 1 | 0 |
| ***Trad. seubertiana*** | 0 | 0 | 1 | 1 | 0 | 0 | 0 | 0 | 2 | 2 | 1 | 0 | - | - | 0 | - | - | 1/2 | 1 | 0/1 | 1 | 0 | 0/1 | 0 | 0 | 0 | 0 | 1 | 0 |
| ***Trad. valida*** | 0 | 0 | 1 | 1 | 0 | 1 | 0 | 0 | 0/1 | 2 | 1 | 0 | - | - | 0 | - | - | 1/2 | 1 | 1/2 | 1 | 0 | 0/1 | 0 | 0 | 0 | 0 | 1 | 0 |
| ***Trad. chrysophylla*** | 0 | 1 | 0 | 0 | 1 | 0 | 0 | 0 | 2 | 2 | 0 | 1 | 2 | 1 | 1 | 2 | 1 | 2 | 1 | 1/2 | 1 | 0 | 1 | 0 | 0 | 0 | 0 | 1 | 0 |
| ***Trad.* sp. 1** | 0 | 0 | 1 | 1 | 1 | 0 | 0 | 0 | 0/2 | 0 | 0 | 1 | 0 | 0 | 1 | 0 | 0 | 2 | 1 | 1/2 | 0 | 0 | 0/1 | 0 | 0 | 0 | 0 | 1 | 0 |
| ***Trad.* sp. 2** | 0 | 0 | 0 | 1 | 1 | 0 | 0 | 1 | 0/2 | 1 | 0 | 1 | 2 | 1 | 1 | 2 | 1 | 0/2 | 1 | 1/2 | 0 | 0 | 0/1 | 0 | 0 | 0 | 0 | 1 | 0 |
| ***Trad. schippii*** | 0 | 0 | 1 | 1 | 1 | 1 | 1 | 1 | 0/3 | 2 | 0 | 0 | - | - | 1 | 2 | 1 | 0 | 0 | 2 | 0 | 1 | 1 | 1 | 0 | 1 | 1 | 1 | 1 |
| ***Trad. zanonia*** | 0 | 0 | 1 | 1 | 1 | 1 | 1 | 1 | 0 | 2 | 0 | 0 | - | - | 1 | 0 | 0 | 0 | 0 | 2 | 0 | 0 | 0/1 | 1 | 0 | 1 | 1 | 1 | 1 |
| ***Trad. commelinoides*** | 1 | 0 | 1 | 0 | 1 | 0 | 0 | 1 | 0/2 | 1 | 0 | 0/1 | 1 | 0 | 0/1 | 1 | 0 | 2 | 1 | 1/2 | 0 | 0 | 0/1 | 0 | 0 | 1 | 0 | 1 | 1 |
| ***Trad. gracilima*** | 0 | 1 | 0 | 0 | 1 | 0 | 0 | 1 | 2 | 1 | 0 | 0/1 | 2 | 0 | 0/1 | 2 | 0 | 2 | 1 | 1 | 0 | 0 | 0 | 0 | 0 | 1 | 0 | 1 | 1 |
| ***Trad. grantii*** | 0 | 0 | 0 | 0 | 1 | 1 | 0 | 1 | 0 | 1 | 0 | 0 | - | - | 1 | 1 | 0 | 0 | 0 | 1/2 | 0 | 0 | 0 | 0 | 0 | 1 | 0 | 1 | 1 |
| ***Trad. poelliae*** | 0 | 0 | 0 | 0 | 1 | 0 | 0 | 1 | 0 | 1 | 0 | 0/1 | 2 | 0 | 0 | - | - | 0 | 1 | 1/2 | 0 | 0 | 1 | 1 | 0 | 0 | 0 | 1 | 0 |
| ***Trad. praetermissa*** | 0 | 0 | 1 | 0 | 1 | 0 | 0 | 1 | 0/2 | 1 | 0 | 0/1 | 2 | 0/1 | 0/1 | 2 | 0/1 | 0/2 | 1 | 1/2 | 0 | 0 | 0/1 | 0 | 0 | 1 | 0 | 1 | 1 |
| ***Trad. standleyi*** | 0 | 0 | 1 | 1 | 1 | 0 | 0 | 0 | 0/2 | 1 | 0 | 1 | 2 | 1 | 1 | 2 | 1 | 0/2 | 1 | 1 | 0 | 0 | 0 | 0 | 0 | 1 | 0 | 1 | 1 |
| ***Trad. guatemalensis*** | 0 | 1 | 0 | 0 | 1 | 1 | 0 | 0 | 0/2 | 1 | 0 | 0/1 | 2 | 0 | 0/1 | 2 | 0 | 0/2 | 1 | 1/2 | 0 | 0 | 0 | 0 | 0 | 0 | 0 | 1 | 0 |
| ***Trad. soconuscana*** | 0 | 0 | 1 | 1 | 1 | 1 | 1 | 1 | 0 | 2 | 0 | 0 | - | - | 1 | 0 | 1 | 0/2 | 1 | 1/2 | 0 | 1 | 0/1 | 0 | 0 | 0 | 0 | 0 | 0 |
| ***Trad. ambigua*** | 1 | 0 | 1 | 1 | 0 | 1 | 0 | 0 | 0 | 2 | 0 | 0/1 | 2 | 0 | 1 | 2 | 0 | 1/2 | 0 | 2 | 0 | 0 | 0/1 | 1 | 0 | 0 | 0 | 0 | 0 |
| ***Trad. boliviana*** | 1 | 0 | 1 | 1 | 0 | 1 | 0 | 0 | 0/2 | 2 | 1 | 0/1 | 0 | 1 | 1 | 0 | 1 | 1/2 | 0 | 2 | 1 | 0 | 0/1 | 1 | 0 | 0 | 0 | 0 | 0 |
| ***Trad. crassifolia*** | 1 | 0 | 1 | 1 | 0 | 1 | 0/1 | 0 | 0/2 | 2 | 1 | 0/1 | 2 | 0 | 1 | 2/3 | 0 | 1 | 0 | 1/2 | 1 | 0 | 0 | 1 | 0 | 0 | 0 | 0 | 0 |
| ***Trad. gentryi*** | 1 | 0 | 1 | 1 | 0 | 1 | 0 | 0 | 0/1 | 2 | 0 | 1 | 1 | 0 | 1 | 1 | 0 | 1 | 0 | 2 | 0 | 0 | 0 | 1 | 0 | 0 | 0 | 0 | 0 |
| ***Trad. petricola*** | 1 | 0 | 1 | 1 | 0 | 1 | 0 | 0 | 0 | 2 | 1 | 0 | - | - | 1 | 1 | 0 | 2 | 0 | 2 | 0 | 0 | 0 | 1 | 0 | 0 | 0 | 0 | 0 |
| ***Trad. tepoxtlana*** | 1 | 0 | 1 | 1 | 0 | 1 | 0 | 0 | 0/1 | 2 | 0 | 0 | - | - | 0 | - | - | 1 | 0 | 2 | 0 | 0 | 0 | 1 | 0 | 0 | 0 | 0 | 0 |
| ***Trad. andrieuxii*** | 1 | 0 | 1 | 1 | 0 | 1 | 0 | 0 | 0/1 | 2 | 1 | 0 | - | - | 1 | 0 | 0 | 0/2 | 0 | 1/2 | 1 | 0 | 0/1 | 1 | 0 | 0 | 0 | 0 | 0 |
| ***Trad. spathacea*** | 0 | 0 | 1 | 1 | 1 | 1 | 1 | 0/1 | 0 | 2 | 0 | 0 | - | - | 0 | - | - | 1 | 0 | 2 | 1 | 0 | 1 | 1 | 0 | 1 | 0 | 1 | 1 |
| ***Trad. virginiana*** | 1 | 0 | 1 | 1 | 0 | 1 | 0 | 0 | 1 | 2 | 1 | 1 | 1 | 0 | 1 | 1 | 0 | 1 | 0 | 2 | 0 | 0 | 0 | 0 | 0 | 0 | 0 | 1 | 0 |
| ***Trad. occidentalis*** | 1 | 0 | 1 | 1 | 0 | 1 | 0 | 0 | 1 | 2 | 1 | 0 | - | - | 0 | - | - | 1 | 0 | 2 | 0 | 0 | 0 | 0 | 0 | 0 | 0 | 1 | 0 |
| ***Trad. sillamontana*** | 1 | 0 | 1 | 1 | 0 | 0 | 1 | 0 | 2 | 2 | 1 | 1 | 3 | 0 | 1 | 3 | 0 | 0/2 | 0 | 0/1 | 0/1 | 0 | 0/1 | 0 | 0 | 0 | 0 | 0 | 0 |
| ***Trad. pinetorum*** | 1 | 0 | 1 | 1 | 0 | 1 | 0 | 0 | 1 | 2 | 1 | 1 | 1 | 0 | 1 | 1 | 0 | 1 | 0 | 2 | 0 | 0 | 0 | 0 | 0 | 0 | 0 | 1 | 0 |
| ***Trad. wrightii*** | 1 | 0 | 1 | 1 | 0 | 1 | 0 | 0 | 1 | 2 | 1 | 0 | - | - | 0 | - | - | 1 | 0 | 2 | 0 | 0 | 0 | 0 | 0 | 0 | 0 | 1 | 0 |
| ***Trad. orchidophylla*** | 1 | 0 | 1 | 1 | 0 | 1 | 1 | 0 | 3 | 2 | 1 | 1 | 2 | 0 | 1 | 2 | 0 | 0 | 0 | 0/1 | 0 | 0 | 0/1 | 0 | 0 | 0 | 0 | 0 | 0 |
| ***Trad. mirandae*** | 1 | 0 | 1 | 1 | 0 | 1 | 1 | 0 | 3 | 2 | 1 | 1 | 2 | 0 | 1 | 2 | 0 | 0 | 0 | 1 | 0 | 0 | 0/1 | 0 | 0 | 0 | 0 | 1 | 0 |
| ***Trad. pygmaea*** | 1 | 0 | 1 | 1 | 0 | 0 | 1 | 0 | 2 | 2 | 1 | 0 | - | - | 0 | - | - | 2 | 0 | 1 | 1 | 0 | 1 | 0 | 0 | 0 | 0 | 1 | 0 |
| ***Trad. brevifolia*** | 1 | 0 | 1 | 1 | 0 | 1 | 0 | 0 | 0/3 | 2 | 1 | 0 | - | - | 0 | - | - | 0/2 | 0 | 1 | 1 | 0 | 0 | 0 | 0 | 0 | 0 | 1 | 0 |
| ***Trad. hirta*** | 1 | 0 | 1 | 1 | 0 | 1 | 1 | 0 | 1 | 2 | 1 | 1 | 2 | 0 | 1 | 2 | 0 | 1 | 0 | 1 | 1 | 0 | 1 | 0 | 0 | 0 | 0 | 1 | 0 |
| ***Trad. pallida*** | 1 | 0 | 1 | 1 | 0 | 1 | 0 | 0 | 0 | 2 | 1 | 0 | - | - | 0 | - | - | 0 | 0 | 0/1 | 1 | 0 | 0/1 | 0 | 0 | 0 | 0 | 1 | 0 |
| ***Trad. zebrina*** | 0 | 1 | 0 | 1 | 1 | 0 | 0/1 | 1 | 0/2 | 1/2 | 0 | 0 | - | - | 0 | - | - | 2 | 1 | 2 | 0 | 1 | 1 | 0 | 0 | 0 | 0 | 1 | 0 |
| ***E. hirsuta*** | 0 | 1 | 0 | 0 | 1 | 1 | 0 | 0 | 0/2 | 1 | 0 | 1 | 2 | 0 | 1 | 2 | 0 | 2 | 1 | 2 | 0 | 0 | 0 | 0/1 | 0 | 0 | 0 | 1 | 0 |
| ***G. consobrina*** | 1 | 1 | 1 | 1 | 1 | 0 | 0 | 0 | 0 | 2 | 0 | 0/1 | 1 | 0 | 0/1 | 1 | 0 | 2 | 1 | 2 | 0 | 0 | 0/1 | 0 | 0 | 0 | 0 | 1 | 0 |
| ***G. geniculata*** | 0 | 1 | 0 | 0 | 1 | 0 | 0 | 0 | 0/1/2 | 1 | 0 | 0/1 | 1/3 | 0 | 1 | 1/3 | 0 | 2 | 1 | 1/2 | 0 | 0 | 0/1 | 0 | 0 | 0 | 0 | 1 | 0 |
| ***G. oxacana*** | 0 | 1 | 1 | 1 | 0 | 1 | 0 | 1 | 0/2 | 2 | 0 | 1 | 1 | 0 | 1 | 1 | 0 | 2 | 0 | 1/2 | 0 | 0 | 0/1 | 0 | 0 | 0 | 0 | 1 | 0 |
| ***G. pellucida*** | 0 | 1 | 0 | 0 | 1 | 0 | 1 | 0 | 0 | 0 | 0 | 0 | - | - | 0 | - | - | 2 | 1 | 1 | 0 | 0 | 1 | 0 | 0 | 0 | 0 | 1 | 0 |
| ***G. karwinskyana*** | 1 | 1 | 1 | 1 | 0 | 1 | 0 | 0 | 0 | 2 | 1 | 0 | - | - | 0 | - | - | 2 | 0 | 2 | 0 | 0 | 0 | 0 | 0 | 0 | 0 | 1 | 0 |
| ***C. filiformis*** | 0 | 0 | 0 | 0 | 1 | 0 | 0 | 0 | 0 | 1 | 1 | 0 | - | - | 0 | - | - | 0 | 0 | 0/1 | 1 | 0 | 0 | 0 | 0 | 0 | 0 | 1 | 0 |
| ***C. fragrans*** | 0 | 0 | 0 | 1 | 1 | 1 | 1 | 0 | 0 | 2 | 1 | 0 | - | 0 | 0 | - | 0 | 1 | 0 | 1/2 | 1 | 0 | 0 | 1 | 0 | 0 | 0 | 1 | 0 |
| ***C. gentlei*** | 0 | 1 | 0 | 1 | 1 | 0 | 1 | 0 | 2 | 2 | 1 | 1 | 0 | - | 1 | 0 | - | 2 | 1 | 1/2 | 0 | 0 | 1 | 1 | 0 | 0 | 0 | 1 | 0 |
| ***C. monandra*** | 0 | 1 | 0 | 1 | 1 | 0 | 1 | 1 | 0/2 | 1 | 0 | 0 | - | - | 0 | - | - | 0 | 1 | 1/2 | 0 | 0 | 0 | 1 | 0 | 0 | 0 | 1 | 0 |
| ***C. repens*** | 0 | 1 | 0 | 1 | 1 | 0 | 1 | 0 | 0/2/3 | 1/2 | 1 | 0 | - | - | 0 | - | - | 0/2 | 1 | 1/2 | 1 | 0 | 1 | 1 | 0 | 0 | 0 | 1 | 0 |
| ***Trip. diuretica*** | 0 | 1 | 0 | 0 | 1 | 0 | 1 | 0 | 0/2 | 0/1 | 0 | 0/1 | 0 | 0 | 0/1 | 0 | 0 | 1/2 | 1 | 1/2 | 0 | 0 | 0/1 | 0 | 0 | 0 | 0 | 1 | 0 |
| ***Trip. elata*** | 0 | 0 | 1 | 1 | 1 | 1 | 0 | 0 | 0/1 | 2 | 1 | 0 | - | - | 0 | - | - | 1 | 0 | 2 | 1 | 0 | 0 | 1 | 0 | 0 | 0 | 1 | 0 |
| ***Trip. glandulosa*** | 0 | 1 | 0 | 1 | 1 | 0 | 0 | 0 | 0/2 | 2 | 0/1 | 0 | - | - | 0 | - | - | 1/2 | 1 | 1 | 1 | 0 | 0 | 0 | 0 | 0 | 0 | 1 | 0 |
| ***Trip. multiflora*** | 0 | 0 | 1 | 1 | 1 | 1 | 0 | 0 | 0/2 | 2 | 1 | 0/1 | 0 | 0 | 1 | 0 | 0 | 2 | 0 | 1 | 1 | 0 | 0 | 0 | 0 | 0 | 0 | 1 | 0 |
| ***Trip. warmingiana*** | 0 | 1 | 0 | 0 | 1 | 0 | 0 | 1 | 0/2 | 1 | 0 | 0 | - | - | 0 | - | - | 2 | 1 | 1/2 | 1 | 0 | 0 | 0 | 0 | 0 | 0 | 1 | 0 |

**Continuation.** Matrix with the 60 terminals and characters 30 to 58. The characters that were not coded due to lack of data of the analyzed specimens and/or from literatures sources are coded as “?”; characters that did not apply were coded as “-”; and the polymorphic characters were coded with a “/” between each state it presented.

| **Taxon** | **30** | **31** | **32** | **33** | **34** | **35** | **36** | **37** | **38** | **39** | **40** | **41** | **42** | **43** | **44** | **45** | **46** | **47** | **48** | **49** | **50** | **51** | **52** | **53** | **54** | **55** | **56** | **57** | **58** |
| --- | --- | --- | --- | --- | --- | --- | --- | --- | --- | --- | --- | --- | --- | --- | --- | --- | --- | --- | --- | --- | --- | --- | --- | --- | --- | --- | --- | --- | --- |
| ***Tin. erecta*** | 1 | 1 | 1 | 0 | 1 | 0 | 1 | 0 | 0 | - | 0 | 0 | 0 | 0 | 0 | 1 | 0 | 0 | 0 | 0 | 1 | 0 | 0 | 0 | 0 | 0 | 0 | 1 | 2/3 |
| ***Tin. sprucei*** | 1 | 1 | 1 | 0 | 1 | 0 | 1 | 0 | 0 | - | 0 | 0 | 0 | 0 | 0 | 1 | 0 | 0 | 0 | 0 | 1 | 0 | 0 | 0 | 0 | 0 | 0 | 1 | 0 |
| ***Trad. tenella*** | 0 | 0 | 0 | 0 | 0 | 1 | 0 | 0 | 1 | 0 | 1 | 0 | 0 | 1 | 0 | 0 | 0 | 0 | 0 | 1 | 2 | 0 | 1 | 0 | 0 | 1 | 1 | 1 | 0/3 |
| ***Trad. cerinthoides*** | 0 | 0 | 0 | 0 | 0 | 1 | 0 | 0 | 1 | 0 | 0 | 1 | 0 | 0 | 0 | 0 | 0 | 0 | 0 | 1 | 2 | 0 | 1 | 0 | 0 | 1 | 0 | 1 | 1/2/3 |
| ***Trad. crassula*** | 0 | 0 | 0 | 0 | 0 | 1 | 0 | 0 | 1 | 0 | 0 | 1 | 0 | 0 | 0 | 0 | 0 | 0 | 0 | 1 | 2 | 0 | 1 | 0 | 0 | 1 | 1 | 1 | 1 |
| ***Trad. fluminensis*** | 0 | 0 | 0 | 0 | 0 | 1 | 0 | 0 | 1 | 0 | 0 | 0 | 0 | 1 | 0 | 0 | 0 | 0 | 0 | 1 | 2 | 0 | 1 | 0 | 0 | 1 | 1 | 1 | 1 |
| ***Trad. mundula*** | 0 | 0 | 0 | 0 | 0 | 1 | 0 | 0 | 1 | 0 | 0 | 0 | 0 | 1 | 0 | 0 | 0 | 0 | 0 | 1 | 2 | 0 | 1 | 0 | 0 | 1 | 1 | 1 | 1 |
| ***Trad. cymbispatha*** | 0 | 0 | 0 | 0 | 0 | 1 | 0 | 0 | 1 | 0 | 0 | 0 | 0 | 1 | 0 | 0 | 0 | 0 | 0 | 1 | 2 | 0 | 1 | 0 | 0 | 1 | 0 | 1 | 1 |
| ***Trad. umbraculifera*** | 0 | 0 | 0 | 0 | 0 | 1 | 0 | 0 | 1 | 1 | 0 | 0 | 0 | 1 | 0 | 0 | 0 | 0 | 0 | 1 | 2 | 0 | 1 | 0 | 0 | 1 | 1 | 1 | 0/1 |
| ***Trad. seubertiana*** | 0 | 0 | 0 | 0 | 0 | 1 | 0 | 0 | 1 | 0 | 1 | 1 | 0 | 0 | 0 | 0 | 0 | 0 | 0 | 1 | 2 | 0 | 1 | 0 | 0 | 1 | 1 | 1 | 0 |
| ***Trad. valida*** | 0 | 0 | 0 | 0 | 0 | 1 | 0 | 1 | 1 | 1 | 0 | 1 | 0 | 0 | 0 | 0 | 0 | 0 | 0 | 1 | 2 | 0 | 1 | 0 | 0 | 1 | 0 | 1 | 0/1 |
| ***Trad. chrysophylla*** | 0 | 0 | 0 | 0 | 0 | 1 | 0 | 0 | 1 | 0 | 1 | 0 | 0 | 1 | 0 | 0 | 0 | 0 | 0 | 1 | 2 | 0 | 1 | 0 | 0 | 1 | 0 | 1 | 2/3 |
| ***Trad.* sp. 1** | 0 | 0 | 0 | 0 | 0 | 1 | 0 | 0 | 1 | 0 | 1 | 0 | 0 | 1 | 0 | 0 | 0 | 0 | 0 | 1 | 2 | 0 | 1 | 0 | 0 | 1 | 1 | 1 | 2 |
| ***Trad.* sp. 2** | 0 | 0 | 0 | 0 | 0 | 1 | 0 | 0 | 1 | 0 | 1 | 0 | 0 | 1 | 0 | 0 | 0 | 0 | 0 | 1 | 2 | 0 | 1 | 0 | 0 | 1 | 1 | 1 | 2/3 |
| ***Trad. schippii*** | 0 | 0 | 0 | 0 | 0 | 1 | 0 | 1 | 1 | 1 | 1 | 0 | 0 | 1 | 1 | 1 | 0 | 1 | 0 | 1 | 0 | 1 | 1 | 1 | 1 | 0 | 0 | 0 | 2 |
| ***Trad. zanonia*** | 0 | 0 | 0 | 0 | 0 | 1 | 0 | 1 | 1 | 1 | 1 | 0 | 0 | 1 | 1 | 1 | 0 | 1 | 0 | 1 | 0 | 1 | 1 | 1 | 1 | 0 | 0 | 2 | 0 |
| ***Trad. commelinoides*** | 0 | 0 | 0 | 0 | 0 | 1 | 0 | 0 | 1 | 1 | 0 | 1 | 1 | 0 | 0 | 1 | 0 | 0 | 0 | 1 | 0 | 1 | 1 | 0 | 1 | 0 | 2 | 1 | 3 |
| ***Trad. gracilima*** | 0 | 0 | 0 | 0 | 0 | 1 | 0 | 1 | 1 | 1 | 0 | 1 | 1 | 0 | 0 | 1 | 0 | 0 | 0 | 1 | 0 | 1 | 1 | 0 | 1 | 0 | 2 | 1 | 1 |
| ***Trad. grantii*** | 0 | 0 | 0 | 0 | 0 | 1 | 0 | 1 | 1 | 1 | 0 | 1 | 1 | 0 | 0 | 1 | 0 | 0 | 0 | 1 | 0 | 1 | 1 | 0 | 1 | 0 | 2 | 1 | 1 |
| ***Trad. poelliae*** | 0 | 0 | 0 | 0 | 0 | 1 | 0 | 0 | 1 | 1 | 0 | 0 | 1 | 0 | 0 | 1 | 0 | 0 | 0 | 1 | 0 | 1 | 1 | 0 | 1 | 0 | 2 | 1 | 1 |
| ***Trad. praetermissa*** | 0 | 0 | 0 | 0 | 0 | 1 | 0 | 1 | 1 | 1 | 0 | 1 | 1 | 0 | 1 | 1 | 0 | 0 | 0 | 1 | 0 | 1 | 1 | 0 | 1 | 0 | 2 | 1 | 1 |
| ***Trad. standleyi*** | 0 | 0 | 0 | 0 | 0 | 1 | 0 | 0 | 1 | 1 | 0 | 0 | 1 | 0 | 0 | 1 | 0 | 0 | 0 | 1 | 0 | 1 | 1 | 0 | 1 | 0 | 2 | 1 | 1 |
| ***Trad. guatemalensis*** | 1 | 2 | 1 | 0 | 1 | 0 | 0 | 0 | 0 | - | 1 | 0 | 0 | 0 | 0 | 0 | 0 | 0 | 0 | 1 | 2 | 0 | 2 | 0 | 0 | 0 | 0 | 1 | 2 |
| ***Trad. soconuscana*** | 0 | 0 | 0 | 0 | 0 | 1 | 0 | 1 | 1 | 1 | 1 | 0 | 0 | 1 | 1 | 1 | 0 | 1 | 0 | 1 | 0 | 1 | 1 | 1 | 1 | 0 | 0 | 0 | 1 |
| ***Trad. ambigua*** | 0 | 0 | 0 | 0 | 0 | 1 | 0 | 1 | 0 | 0 | 1 | 1 | 0 | 0 | 0 | 1 | 0 | 0 | 0 | 1 | 2 | 0 | 1 | 0 | 0 | 0 | 0 | 1 | 1 |
| ***Trad. boliviana*** | 0 | 0 | 0 | 0 | 0 | 1 | 0 | 1 | 0 | 0 | 1 | 1 | 0 | 0 | 0 | 1 | 0 | 0 | 0 | 1 | 2 | 0 | 1 | 0 | 0 | 0 | 0 | 1 | 2/3 |
| ***Trad. crassifolia*** | 0 | 0 | 0 | 0 | 0 | 1 | 0 | 1 | 0 | 0 | 1 | 1 | 0 | 0 | 0 | 1 | 0 | 1 | 0 | 1 | 2 | 0 | 1 | 0 | 0 | 0 | 0 | 1 | 1 |
| ***Trad. gentryi*** | 0 | 0 | 0 | 0 | 0 | 1 | 0 | 1 | 0 | 0 | 1 | 1 | 0 | 0 | 0 | 1 | 0 | 0 | 0 | 1 | 2 | 0 | 1 | 0 | 0 | 0 | 0 | 1 | 1 |
| ***Trad. petricola*** | 0 | 0 | 0 | 0 | 0 | 1 | 0 | 1 | 0 | 0 | 1 | 1 | 0 | 0 | 0 | 1 | 0 | 0 | 0 | 1 | 2 | 0 | 1 | 0 | 0 | 0 | 0 | 1 | 1 |
| ***Trad. tepoxtlana*** | 0 | 0 | 0 | 0 | 0 | 1 | 0 | 1 | 0 | 0 | 1 | 1 | 0 | 0 | 0 | 1 | 0 | 0 | 0 | 1 | 2 | 0 | 1 | 0 | 0 | 0 | 0 | 1 | 0/1 |
| ***Trad. andrieuxii*** | 0 | 0 | 0 | 0 | 0 | 1 | 0 | 1 | 0 | 0 | 1 | 1 | 0 | 0 | 0 | 1 | 0 | 1 | 0 | 1 | 2 | 0 | 1 | 0 | 0 | 0 | 0 | 1 | 0/1 |
| ***Trad. spathacea*** | 0 | 0 | 0 | 0 | 0 | 1 | 0 | 1 | 1 | 1 | 1 | 1 | 0 | 0 | 1 | 1 | 0 | 0 | 0 | 1 | 2 | 1 | 1 | 0 | 1 | 0 | 0 | 0 | 0 |
| ***Trad. virginiana*** | 0 | 0 | 0 | 0 | 0 | 1 | 0 | 0 | 1 | 0 | 1 | 1 | 0 | 0 | 1 | 1 | 0 | 0 | 0 | 0 | 2 | 0 | 1 | 0 | 0 | 0 | 0 | 0 | 1 |
| ***Trad. occidentalis*** | 0 | 0 | 0 | 0 | 0 | 1 | 0 | 0 | 1 | 0 | 1 | 1 | 0 | 1 | 1 | 1 | 0 | 0 | 0 | 0 | 2 | 0 | 1 | 0 | 0 | 0 | 0 | 0 | 1/3 |
| ***Trad. sillamontana*** | 0 | 0 | 0 | 0 | 0 | 1 | 0 | 0 | 1 | 0 | 1 | 1 | 0 | 1 | 1 | 1 | 0 | 1 | 0 | 1 | 1 | 0 | 1 | 0 | 0 | 0 | 0 | 0 | 1 |
| ***Trad. pinetorum*** | 0 | 0 | 0 | 0 | 0 | 1 | 0 | 0 | 1 | 0 | 1 | 1 | 0 | 1 | 1 | 1 | 0 | 0 | 0 | 0 | 2 | 0 | 1 | 0 | 0 | 0 | 0 | 0 | 3 |
| ***Trad. wrightii*** | 0 | 0 | 0 | 0 | 0 | 1 | 0 | 0 | 1 | 0 | 1 | 1 | 0 | 1 | 1 | 1 | 0 | 0 | 0 | 0 | 2 | 0 | 1 | 0 | 0 | 0 | 0 | 0 | 0/3 |
| ***Trad. orchidophylla*** | 0 | 0 | 0 | 0 | 0 | 1 | 0 | 0 | 1 | 0 | 1 | 1 | 0 | 1 | 1 | 1 | 0 | 1 | 0 | 1 | 2 | 0 | 1 | 0 | 0 | 0 | 0 | 0 | 2 |
| ***Trad. mirandae*** | 0 | 0 | 0 | 0 | 0 | 1 | 0 | 0 | 1 | 0 | 1 | 1 | 0 | 1 | 1 | 1 | 0 | 1 | 0 | 1 | 2 | 0 | 1 | 0 | 0 | 0 | 0 | 0 | 2 |
| ***Trad. pygmaea*** | 0 | 0 | 0 | 0 | 0 | 1 | 0 | 0 | 1 | 0 | 1 | 1 | 0 | 1 | 1 | 1 | 0 | 1 | 0 | 1 | 1 | 0 | 1 | 0 | 0 | 0 | 0 | 0 | 1 |
| ***Trad. brevifolia*** | 0 | 0 | 0 | 0 | 0 | 1 | 0 | 0 | 1 | 0 | 1 | 1 | 0 | 1 | 1 | 1 | 0 | 1 | 0 | 1 | 1 | 0 | 1 | 0 | 0 | 0 | 0 | 0 | 0 |
| ***Trad. hirta*** | 0 | 0 | 0 | 0 | 0 | 1 | 0 | 0 | 1 | 0 | 1 | 1 | 0 | 1 | 1 | 1 | 0 | 1 | 0 | 1 | 1 | 0 | 1 | 0 | 0 | 0 | 0 | 0 | 0 |
| ***Trad. pallida*** | 0 | 0 | 0 | 0 | 0 | 1 | 0 | 0 | 1 | 0 | 1 | 1 | 0 | 1 | 1 | 1 | 0 | 1 | 0 | 1 | 1 | 0 | 1 | 0 | 0 | 0 | 0 | 0 | 1 |
| ***Trad. zebrina*** | 0 | 0 | 0 | 0 | 0 | 1 | 0 | 0 | 1 | 1 | 1 | 1 | 0 | 1 | 1 | 1 | 0 | 1 | 0 | 1 | 0 | 1 | 1 | 1 | 1 | 2 | 2 | 0 | 1 |
| ***E. hirsuta*** | 1 | 2 | 1 | 0 | 1 | 0 | 0 | 0 | 0 | - | 1 | 0 | 0 | 0 | 0 | 0 | 0 | 0 | 0 | 1 | 2 | 0 | 2 | 0 | 0 | 0 | 0 | 1 | 2/3 |
| ***G. consobrina*** | 1 | 1 | 2 | 1 | 1 | 0 | 0 | 0 | 0 | - | 1 | 0 | 0 | 0 | 0 | 0 | 0 | 0 | 0 | 1 | 2 | 0 | 3 | 0 | 0 | 0 | 0 | 1 | 1 |
| ***G. geniculata*** | 0 | 1 | 2 | 1 | 1 | 0 | 0 | 0 | 0 | - | 1 | 0 | 0 | 0 | 0 | 0 | 0 | 0 | 0 | 1 | 2 | 0 | 3 | 0 | 0 | 0 | 0 | 1 | 2/3 |
| ***G. oxacana*** | 1 | 1 | 2 | 1 | 1 | 0 | 0 | 0 | 0 | - | 1 | 0 | 0 | 0 | 0 | 0 | 0 | 0 | 0 | 1 | 2 | 0 | 3 | 0 | 0 | 0 | 0 | 1 | 0 |
| ***G. pellucida*** | 0 | 1 | 2 | 1 | 1 | 0 | 0 | 0 | 0 | - | 1 | 0 | 0 | 0 | 0 | 0 | 0 | 0 | 0 | 1 | 2 | 0 | 3 | 0 | 0 | 0 | 0 | 1 | 0 |
| ***G. karwinskyana*** | 1 | 1 | 2 | 1 | 1 | 0 | 0 | 0 | 0 | - | 1 | 0 | 0 | 0 | 0 | 0 | 0 | 0 | 0 | 1 | 2 | 0 | 3 | 0 | 0 | 0 | 0 | 1 | 0 |
| ***C. filiformis*** | 0 | 0 | 0 | 0 | 0 | 1 | 0 | 0 | 0 | - | 0 | 0 | 0 | 0 | 0 | 0 | 0 | 0 | 1 | 1 | 2 | 0 | 2 | 0 | 0 | 0 | 0 | 1 | 3 |
| ***C. fragrans*** | 0 | 0 | 0 | 0 | 0 | 1 | 0 | 0 | 0 | - | 0 | 0 | 0 | 0 | 0 | 1 | 1 | 1 | 0 | 0 | 0 | 0 | 2 | 0 | 0 | 0 | 0 | 1 | 1 |
| ***C. gentlei*** | 0 | 0 | 0 | 0 | 0 | 1 | 0 | 0 | 0 | - | 0 | 0 | 0 | 0 | 0 | 1 | 0 | 1 | 0 | 0 | 0 | 0 | 2 | 0 | 0 | 0 | 0 | 1 | 1 |
| ***C. monandra*** | 0 | 0 | 0 | 0 | 0 | 1 | 0 | 0 | 0 | - | 0 | 0 | 0 | 0 | 0 | 1 | 0 | 0 | 0 | 0 | 2 | 0 | 2 | 0 | 0 | 2 | 0 | 1 | 3 |
| ***C. repens*** | 0 | 0 | 0 | 0 | 0 | 1 | 0 | 0 | 0 | - | 0 | 0 | 0 | 0 | 0 | 1 | 1 | 1 | 0 | 0 | 0 | 0 | 2 | 0 | 0 | 2 | 0 | 1 | 1 |
| ***Trip. diuretica*** | 0 | 0 | 0 | 0 | 0 | 1 | 0 | 0 | 0 | - | 0 | 0 | 0 | 0 | 0 | 0 | 0 | 0 | 1 | 1 | 2 | 0 | 2 | 0 | 0 | 0 | 0 | 1 | 0 |
| ***Trip. elata*** | 0 | 0 | 0 | 0 | 0 | 1 | 0 | 0 | 0 | - | 0 | 0 | 0 | 0 | 0 | 0 | 0 | 0 | 1 | 1 | 1 | 0 | 2 | 0 | 0 | 0 | 0 | 1 | 1 |
| ***Trip. glandulosa*** | 0 | 0 | 0 | 0 | 0 | 1 | 0 | 0 | 0 | - | 0 | 0 | 0 | 0 | 0 | 0 | 0 | 0 | 1 | 1 | 1 | 0 | 2 | 0 | 0 | 0 | 0 | 1 | 3 |
| ***Trip. multiflora*** | 0 | 0 | 0 | 0 | 0 | 1 | 0 | 0 | 0 | - | 0 | 0 | 0 | 0 | 0 | 0 | 0 | 0 | 1 | 1 | 1 | 0 | 2 | 0 | 0 | 0 | 0 | 1 | 3 |
| ***Trip. warmingiana*** | 0 | 0 | 0 | 0 | 0 | 1 | 0 | 0 | 0 | - | 0 | 0 | 0 | 0 | 0 | 0 | 0 | 0 | 1 | 1 | 2 | 0 | 2 | 0 | 0 | 0 | 0 | 1 | 1 |

**Continuation.** Matrix with the 60 terminals and characters 59 to 87. The characters that were not coded due to lack of data of the analyzed specimens and/or from literatures sources are coded as “?”; characters that did not apply were coded as “-”; and the polymorphic characters were coded with a “/” between each state it presented.

| **Taxon** | **59** | **60** | **61** | **62** | **63** | **64** | **65** | **66** | **67** | **68** | **69** | **70** | **71** | **72** | **73** | **74** | **75** | **76** | **77** | **78** | **79** | **80** | **81** | **82** | **83** | **84** | **85** | **86** | **87** |
| --- | --- | --- | --- | --- | --- | --- | --- | --- | --- | --- | --- | --- | --- | --- | --- | --- | --- | --- | --- | --- | --- | --- | --- | --- | --- | --- | --- | --- | --- |
| ***Tin. erecta*** | 1/2 | 0 | 1/3 | 0 | 1 | 0/2 | 0 | 0 | 1/2 | 1 | 0 | 1 | 1 | 2 | 0 | 1 | 0 | 1 | 0 | - | 0 | - | 3 | 3 | 1 | 3 | 0 | 2 | 1 |
| ***Tin. sprucei*** | 1 | 0 | 1/3 | 0 | 1 | 2 | 0 | 0 | 1/2 | 1 | 0 | 1 | 1 | 2 | 0 | 1 | 0 | 1 | 0 | - | 0 | - | 3 | 3 | 0 | 3 | 0 | 2 | 1 |
| ***Trad. tenella*** | 1 | 0 | 0 | 0 | 0 | 1/2 | 0 | 1 | 0 | 2 | 1 | 0 | 2 | 0 | 1 | 0 | 0 | 0 | 1 | 3 | 1 | 3 | 1 | 1 | 1 | 2 | 0 | 1 | 0 |
| ***Trad. cerinthoides*** | 1/2 | 0 | 0 | 0 | 0 | 1/2 | 0 | 1 | 0 | 2 | 1 | 0 | 2 | 0 | 1 | 0 | 0 | 0 | 1 | 3 | 1 | 3 | 1 | 1 | 1 | 2 | 0 | 2 | 0 |
| ***Trad. crassula*** | 1 | 0 | 0 | 0 | 0 | 1 | 0 | 1 | 0 | 2 | 1 | 0 | 2 | 0 | 1 | 0 | 0 | 0 | 1 | 3 | 1 | 3 | 1 | 1 | 1 | 2 | 0 | 2 | 0 |
| ***Trad. fluminensis*** | 1 | 0 | 0 | 0 | 0 | 1 | 0 | 1 | 0 | 2 | 1 | 0 | 2 | 0 | 1 | 0 | 0 | 0 | 1 | 3 | 1 | 3 | 1 | 1 | 1 | 2 | 0 | 1 | 0 |
| ***Trad. mundula*** | 1 | 0 | 0 | 0 | 0 | 1 | 0 | 1 | 0 | 2 | 1 | 0 | 2 | 0 | 1 | 0 | 0 | 0 | 1 | 3 | 1 | 3 | 1 | 1 | 1 | 2 | 0 | 1 | 0 |
| ***Trad. cymbispatha*** | 2 | 0 | 0 | 0 | 0 | 1 | 0 | 1 | 0 | 2 | 1 | 0 | 2 | 0 | 1 | 0 | 0 | 0 | 1 | 3 | 1 | 3 | 1 | 1 | 1 | 2 | 0 | 1 | 0 |
| ***Trad. umbraculifera*** | 1 | 0 | 0 | 0 | 0 | 1 | 0 | 1 | 0 | 2 | 1 | 0 | 2 | 0 | 1 | 0 | 0 | 0 | 1 | 3 | 1 | 3 | 1 | 1 | 1 | 2 | 0 | 1 | 0 |
| ***Trad. seubertiana*** | 1/2 | 0 | 0 | 0 | 0 | 2 | 0 | 1 | 0 | 2 | 1 | 0 | 2 | 0 | 1 | 0 | 0 | 0 | 1 | 3 | 1 | 3 | 1 | 1 | 1 | 2 | 0 | 2 | 0 |
| ***Trad. valida*** | 1 | 0 | 0 | 0 | 0 | 1/2 | 0 | 1 | 0 | 2 | 1 | 0 | 2 | 0 | 1 | 0 | 0 | 0 | 1 | 3 | 1 | 3 | 1 | 1 | 1 | 2 | 0 | 2 | 0 |
| ***Trad. chrysophylla*** | 2 | 0 | 0 | 0 | 0 | 1 | 0 | 1 | 0 | 2 | 1 | 0 | 2 | 0 | 1 | 0 | 0 | 0 | 1 | 3 | 1 | 3 | 1 | 1 | 1 | 2 | 0 | 1 | 0 |
| ***Trad.* sp. 1** | 1 | 0 | 0 | 0 | 0 | 1 | 0 | 1 | 0 | 2 | 1 | 0 | 2 | 0 | 1 | 0 | 0 | 0 | 1 | 3 | 1 | 3 | 1 | 1 | 1 | 2 | 0 | 1 | 0 |
| ***Trad.* sp. 2** | 1 | 0 | 0 | 0 | 0 | 1/2 | 0 | 1 | 0 | 2 | 1 | 0 | 2 | 0 | 1 | 0 | 0 | 0 | 1 | 3 | 1 | 3 | 1 | 1 | 1 | 2 | 0 | 1 | 0 |
| ***Trad. schippii*** | 0 | 1 | ? | 1 | 0 | 1 | 0 | 0 | 1 | 0 | 0 | 1 | 0 | 1 | 1 | 0 | 1 | 0 | 1 | 0 | 1 | 0 | 0 | 0 | 0 | 2 | 0 | 2 | 0 |
| ***Trad. zanonia*** | 2 | 0 | 1/3 | 0 | 0 | 1 | 0 | 0 | 1 | 0 | 0 | 1 | 0 | 1 | 1 | 0 | 0 | 0 | 1 | 0 | 1 | 0 | 0 | 0 | 0 | 2 | 0 | 2 | 0 |
| ***Trad. commelinoides*** | 1/2 | 0 | 3 | 0 | 0 | 2 | 0 | 0 | 0 | 1 | 0 | 0 | 1 | 1 | 1 | 0 | 0 | 0 | 1 | 0 | 1 | 0 | 0 | 0 | 0 | 2 | 0 | 0 | 0 |
| ***Trad. gracilima*** | 1 | 0 | 0 | 0 | 0 | 1 | 0 | 0 | - | - | 0 | - | - | 1 | 1 | 0 | 0 | 0 | 1 | 0 | 1 | 0 | 0 | 0 | 0 | 2 | 0 | 0 | 0 |
| ***Trad. grantii*** | 1 | 0 | 0/1 | 0 | 0 | 1 | 0 | 0 | - | - | 0 | - | - | 1 | 1 | 0 | 0 | 0 | 1 | 0 | 1 | 0 | 0 | 0 | 0 | 2 | 0 | 0 | 0 |
| ***Trad. poelliae*** | 1 | 0 | 3 | 0 | 0 | 2 | 0 | 0 | 1 | 0 | 0 | 1 | 0 | 1 | 1 | 0 | 0 | 0 | 1 | 0 | 1 | 0 | 0 | 0 | 0 | 2 | 0 | 0 | 0 |
| ***Trad. praetermissa*** | 1 | 0 | 3 | 0 | 0 | 1 | 0 | 0 | 2 | 0 | 0 | 2 | 0 | 1 | 1 | 0 | 0 | 0 | 1 | 0 | 1 | 0 | 0 | 0 | 0 | 2 | 0 | 0 | 0 |
| ***Trad. standleyi*** | 1 | 0 | 1/3 | 0 | 0 | 2 | 0 | 0 | 1 | 1 | 0 | 1 | 1 | 1 | 1 | 0 | 0 | 0 | 1 | 0 | 1 | 0 | 0 | 0 | 0 | 2 | 0 | 0 | 0 |
| ***Trad. guatemalensis*** | 1 | 0 | 0 | 0 | 0 | 0/1 | 0 | 1 | 1 | 1 | 1 | 1 | 1 | 0 | 1 | 0 | 0 | 0 | 0 | - | 0 | - | 3 | 3 | 1 | 2 | 2 | 1 | 0 |
| ***Trad. soconuscana*** | 0 | 0 | 0 | 1 | 0 | 1/2 | 0 | 0 | - | - | 0 | - | - | 1 | 1 | 0 | 1 | 0 | 1 | 0 | 1 | 0 | 0 | 0 | 0 | 2 | 0 | 2 | 0 |
| ***Trad. ambigua*** | 1/2 | 0 | 0/3 | 0 | 0 | 1/2 | 0 | 1 | 1 | 0 | 0 | 1 | 0 | 0 | 1 | 2 | 0 | 0 | 1 | 1/3 | 1 | 1/3 | 2 | 2 | 1 | 2 | 1 | 3 | 0 |
| ***Trad. boliviana*** | 1/2 | 0 | 0/1/3 | 0 | 0 | 2 | 0 | 1 | 1 | 0 | 0 | 1 | 0 | 0 | 1 | 2 | 0 | 0 | 1 | 1 | 1 | 1 | 2 | 2 | 1 | 2 | 1 | 3 | 0 |
| ***Trad. crassifolia*** | 1 | 0 | 0/1/3 | 0 | 0 | 0/2 | 0 | 1 | 1 | 0 | 0 | 1 | 0 | 0 | 1 | 2 | 0 | 0 | 1 | 1 | 1 | 1 | 2 | 2 | 1 | 2 | 1 | 3 | 0 |
| ***Trad. gentryi*** | 1 | 0 | 0 | 0 | 0 | 2 | 0 | 1 | 1 | 0 | 0 | 1 | 0 | 0 | 1 | 2 | 0 | 0 | 1 | 1/3 | 1 | 1/3 | 2 | 2 | 1 | 2 | 1 | 3 | 0 |
| ***Trad. petricola*** | 1 | 0 | 0/2 | 0 | 0 | 2 | 0 | 1 | 1 | 0 | 0 | 1 | 0 | 0 | 1 | 2 | 0 | 0 | 1 | 1/3 | 1 | 1/3 | 2 | 2 | 1 | 2 | 1 | 3 | 0 |
| ***Trad. tepoxtlana*** | 1 | 0 | 0 | 0 | 0 | 2 | 0 | 1 | 1 | 0 | 0 | 1 | 0 | 0 | 1 | 2 | 0 | 0 | 1 | 1/3 | 1 | 1/3 | 2 | 2 | 1 | 2 | 1 | 3 | 0 |
| ***Trad. andrieuxii*** | 1 | 1 | 0/1/3 | 1 | 0 | 0/2 | 0 | 1 | 1 | 0 | 0 | 1 | 0 | 0 | 1 | 2 | 1 | 0 | 1 | 1 | 1 | 1 | 2 | 2 | 1 | 2 | 1 | 3 | 0 |
| ***Trad. spathacea*** | 0 | 0 | 0 | 0 | 0 | 1 | 0 | 0 | 1 | 0 | 0 | 1 | 0 | 1 | 1 | 0 | 0 | 0 | 1 | 3 | 1 | 3 | 1 | 1 | 0 | 2 | 0 | 2 | 0 |
| ***Trad. virginiana*** | 1 | 0 | 1/3 | 0 | 0 | 0/1/2 | 0 | 1 | 1 | 1 | 1 | 1 | 1 | 0 | 1 | 0 | 0 | 0 | 1 | 1 | 1 | 1 | 2 | 2 | 1 | 2 | 0 | 1 | 0 |
| ***Trad. occidentalis*** | 1 | 0 | 1/3 | 0 | 0 | 0/2 | 0 | 1 | 1 | 1 | 1 | 1 | 1 | 0 | 1 | 0 | 0 | 0 | 1 | 1 | 1 | 1 | 2 | 2 | 1 | 2 | 0 | 1 | 0 |
| ***Trad. sillamontana*** | 0/1 | 0 | 1/3 | 0 | 0 | 2 | 0 | 0 | 1 | 0 | 0 | 1 | 0 | 0 | 1 | 0 | 1 | 0 | 1 | 1 | 1 | 1 | 2 | 2 | 1 | 2 | 1 | 1 | 0 |
| ***Trad. pinetorum*** | 1 | 0 | 1/3 | 0 | 0 | 0/2 | 0 | 1 | 1 | 1 | 1 | 1 | 1 | 0 | 1 | 0 | 0 | 0 | 1 | 1 | 1 | 1 | 2 | 2 | 1 | 2 | 0 | 1 | 0 |
| ***Trad. wrightii*** | 1 | 0 | 1/3 | 0 | 0 | 2 | 0 | 1 | 1 | 1 | 1 | 1 | 1 | 0 | 1 | 0 | 0 | 0 | 1 | 1 | 1 | 1 | 2 | 2 | 1 | 2 | 0 | 1 | 0 |
| ***Trad. orchidophylla*** | 0/1 | 0 | 0 | 0 | 0 | 1 | 0 | 0 | - | - | 0 | - | - | 0 | 1 | 0 | 1 | 0 | 1 | 1/3 | 1 | 1/3 | 2 | 2 | 1 | 2 | 1 | 1 | 0 |
| ***Trad. mirandae*** | 0/1 | 0 | 0/3 | 0 | 0 | 1/2 | 0 | 0 | - | - | 0 | - | - | 0 | 1 | 0 | 1 | 0 | 1 | 1/3 | 1 | 1/3 | 2 | 2 | 1 | 2 | 1 | 1 | 0 |
| ***Trad. pygmaea*** | 0 | 1 | 1/3 | 1 | 0 | 2 | 0 | 1 | - | - | 0 | - | - | 0 | 1 | 0 | 1 | 0 | 1 | 1/3 | 1 | 1/3 | 2 | 2 | 1 | 2 | 1 | 1 | 0 |
| ***Trad. brevifolia*** | 0 | 1 | 0/1 | 1 | 0 | 1/2 | 0 | 1 | - | - | 0 | - | - | 0 | 1 | 0 | 1 | 0 | 1 | 1 | 1 | 1 | 2 | 2 | 1 | 2 | 1 | 1 | 0 |
| ***Trad. hirta*** | 0 | 1 | 0/1/3 | 1 | 0 | 2 | 0 | 1 | 1 | 0 | 0 | 1 | 0 | 0 | 1 | 0 | 1 | 0 | 1 | 1/3 | 1 | 1/3 | 2 | 2 | 1 | 2 | 1 | 1 | 0 |
| ***Trad. pallida*** | 0 | 0/1 | 0/1 | 1 | 0 | 2 | 0 | 0 | 1 | 0 | 0 | 1 | 0 | 0 | 1 | 0 | 1 | 0 | 1 | 1 | 1 | 1 | 2 | 2 | 1 | 2 | 1 | 1 | 0 |
| ***Trad. zebrina*** | 0 | 1 | 0/1 | 1 | 0 | 2 | 0 | 0 | 1 | 0 | 0 | 1 | 0 | 1 | 1 | 0 | 1 | 0 | 1 | 0 | 1 | 0 | 0 | 0 | 0 | 2 | 0 | 2 | 0 |
| ***E. hirsuta*** | 2 | 0 | 0 | 0 | 0 | 2 | 0 | 1 | 1 | 1 | 1 | 1 | 1 | 0 | 1 | 0 | 0 | 0 | 0 | - | 0 | - | 3 | 3 | 1 | 2 | 2 | 1 | 0 |
| ***G. consobrina*** | 1 | 0 | 0 | 0 | 0 | 2 | 0 | 0 | 1 | 1 | 0 | 1 | 1 | 0 | 1 | 0 | 0 | 0 | 1 | 3 | 1 | 3 | 1 | 1 | 1 | 2 | 2 | 1 | 0 |
| ***G. geniculata*** | 1 | 0 | 0 | 0 | 0 | 1 | 0 | 0 | 1 | 1 | 0 | 1 | 1 | 0 | 1 | 0 | 0 | 0 | 1 | 3 | 1 | 3 | 1 | 1 | 1 | 2 | 2 | 1 | 0 |
| ***G. oxacana*** | 1 | 0 | 0 | 0 | 0 | 1/2 | 0 | 0 | 0/2 | 1 | 0 | 0/2 | 1 | 0 | 1 | 0 | 0 | 0 | 1 | 0/3 | 1 | 0/3 | 1 | 1 | 1 | 2 | 0 | 1 | 0 |
| ***G. pellucida*** | 1 | 0 | 0/3 | 0 | 0 | 1 | 0 | 0 | 0/2 | 1 | 0 | 0/2 | 1 | 0 | 1 | 0 | 0 | 0 | 1 | 3 | 1 | 3 | 1 | 1 | 1 | 2 | 0 | 1 | 0 |
| ***G. karwinskyana*** | 2 | 0 | 0/3 | 0 | 0 | 1/2 | 0 | 0 | 1 | 1 | 0 | 1 | 1 | 0 | 1 | 0 | 0 | 0 | 1 | 3 | 1 | 3 | 1 | 1 | 1 | 2 | 2 | 1 | 0 |
| ***C. filiformis*** | 1 | 0 | 1/3 | 0 | 0 | 1/2 | 0 | 0 | - | - | 0 | - | - | 1 | 0 | 1 | 0 | 1 | 1 | 2/3 | 1 | 2/3 | 1 | 1 | 0 | ? | 0 | 0 | 0 |
| ***C. fragrans*** | 2 | 0 | 0/2 | 0 | 0 | 1 | 0 | 0 | - | - | 0 | - | - | 0 | 1 | 0 | 0 | 1 | 1 | 2 | 1 | 2 | 1 | 1 | 0 | 1 | 0 | 0 | 0 |
| ***C. gentlei*** | 1 | 0 | 0/3 | 0 | 0 | 1 | 0 | 0 | - | - | 0 | - | - | 1 | 0 | 0 | 0 | 1 | 1 | 0 | 1 | 0 | 0 | 0 | 0 | 1 | 0 | 0 | 0 |
| ***C. monandra*** | 1 | 0 | 2 | 0 | 0 | 3 | 1 | 0 | - | - | 0 | - | - | - | 1 | 0 | 0 | 1 | 0 | - | - | - | 3 | - | 0 | 1 | 2 | 0 | 0 |
| ***C. repens*** | 1 | 0 | 2 | 0 | 0 | 3 | 0/1 | 0 | - | - | 0 | - | - | 0 | 1 | 0 | 0 | 1 | 1 | 2 | 1 | 2 | 1 | 1 | 0 | 1 | 0 | 0 | 0 |
| ***Trip. diuretica*** | 1/2 | 0 | 0/1 | 0 | 0 | 1/2 | 0 | 0 | - | - | 0 | 1 | 1 | 1 | 0 | 1 | 0 | 1 | 0 | - | 0 | - | 3 | 3 | 0 | 0 | 0 | 0 | 0 |
| ***Trip. elata*** | 1/2 | 0 | 1 | 0 | 0 | 1/2 | 0 | 0 | - | - | 0 | 1 | 1 | 1 | 0 | 1 | 0 | 1 | 0 | - | 1 | 2 | 3 | 1 | 0 | 0 | 0 | 0 | 0 |
| ***Trip. glandulosa*** | 1 | 0 | 0/1 | 0 | 0 | 1/2 | 0 | 0 | 1 | 1 | 0 | - | - | 1 | 0 | 1 | 0 | 1 | 0 | - | 1 | 2 | 3 | 1 | 0 | 0 | 0 | 0 | 0 |
| ***Trip. multiflora*** | 1/2 | 0 | 1 | 0 | 0 | 2 | 0 | 0 | 1 | 1 | 0 | - | - | 1 | 0 | 1 | 0 | 1 | 0 | - | 1 | 2 | 3 | 1 | 0 | 0 | 0 | 0 | 0 |
| ***Trip. warmingiana*** | 1/2 | 0 | 0 | 0 | 0 | 2 | 0 | 0 | - | - | 0 | 1 | 1 | 1 | 0 | 1 | 0 | 1 | 0 | - | 1 | 2 | 3 | 1 | 0 | 0 | 0 | 0 | 0 |

**Continuation.** Matrix with the 60 terminals and characters 88 to 114. The characters that were not coded due to lack of data of the analyzed specimens and/or from literatures sources are coded as “?”; characters that did not apply were coded as “-”; and the polymorphic characters were coded with a “/” between each state it presented.

| **Taxon** | **88** | **89** | **90** | **91** | **92** | **93** | **94** | **95** | **96** | **97** | **98** | **99** | **100** | **101** | **102** | **103** | **104** | **105** | **106** | **107** | **108** | **109** | **110** | **111** | **112** | **113** | **114** |
| --- | --- | --- | --- | --- | --- | --- | --- | --- | --- | --- | --- | --- | --- | --- | --- | --- | --- | --- | --- | --- | --- | --- | --- | --- | --- | --- | --- |
| ***Tin. erecta*** | 1/2 | 0 | 0 | 0 | 0 | 2 | 0 | 0 | - | - | 0 | 1 | 1 | 1 | 0 | 1 | 0 | 1 | 0 | - | 1 | 2 | 3 | 1 | 0 | 0 | 0 |
| ***Tin. sprucei*** | 1/2 | 0 | 0 | 0 | 0 | 2 | 0 | 0 | - | - | 0 | 1 | 1 | 1 | 0 | 1 | 0 | 1 | 0 | - | 1 | 2 | 3 | 1 | 0 | 0 | 0 |
| ***Trad. tenella*** | 1/2 | 0 | 0 | 0 | 0 | 2 | 0 | 0 | - | - | 0 | 1 | 1 | 1 | 0 | 1 | 0 | 1 | 0 | - | 1 | 2 | 3 | 1 | 0 | 1 | 0 |
| ***Trad. cerinthoides*** | 1/2 | 0 | 0 | 0 | 0 | 2 | 0 | 0 | - | - | 0 | 1 | 1 | 1 | 0 | 1 | 0 | 1 | 0 | - | 1 | 2 | 3 | 1 | 0 | 0 | 1 |
| ***Trad. crassula*** | 1/2 | 0 | 0 | 0 | 0 | 2 | 0 | 0 | - | - | 0 | 1 | 1 | 1 | 0 | 1 | 0 | 1 | 0 | - | 1 | 2 | 3 | 1 | 0 | 0 | 1 |
| ***Trad. fluminensis*** | 1/2 | 0 | 0 | 0 | 0 | 2 | 0 | 0 | - | - | 0 | 1 | 1 | 1 | 0 | 1 | 0 | 1 | 0 | - | 1 | 2 | 3 | 1 | 0 | 1 | 0 |
| ***Trad. mundula*** | 1/2 | 0 | 0 | 0 | 0 | 2 | 0 | 0 | - | - | 0 | 1 | 1 | 1 | 0 | 1 | 0 | 1 | 0 | - | 1 | 2 | 3 | 1 | 0 | 1 | 0 |
| ***Trad. cymbispatha*** | 1/2 | 0 | 0 | 0 | 0 | 2 | 0 | 0 | - | - | 0 | 1 | 1 | 1 | 0 | 1 | 0 | 1 | 0 | - | 1 | 2 | 3 | 1 | 0 | 1 | 0 |
| ***Trad. umbraculifera*** | 1/2 | 0 | 0 | 0 | 0 | 2 | 0 | 0 | - | - | 0 | 1 | 1 | 1 | 0 | 1 | 0 | 1 | 0 | - | 1 | 2 | 3 | 1 | 0 | 1 | 0 |
| ***Trad. seubertiana*** | 1/2 | 0 | 0 | 0 | 0 | 2 | 0 | 0 | - | - | 0 | 1 | 1 | 1 | 0 | 1 | 0 | 1 | 0 | - | 1 | 2 | 3 | 1 | 0 | 0 | 1 |
| ***Trad. valida*** | 1/2 | 0 | 0 | 0 | 0 | 2 | 0 | 0 | - | - | 0 | 1 | 1 | 1 | 0 | 1 | 0 | 1 | 0 | - | 1 | 2 | 3 | 1 | 0 | 0 | 1 |
| ***Trad. chrysophylla*** | 1/2 | 0 | 0 | 0 | 0 | 2 | 0 | 0 | - | - | 0 | 1 | 1 | 1 | 0 | 1 | 0 | 1 | 0 | - | 1 | 2 | 3 | 1 | 0 | 1 | 0 |
| ***Trad.* sp. 1** | 1/2 | 0 | 0 | 0 | 0 | 2 | 0 | 0 | - | - | 0 | 1 | 1 | 1 | 0 | 1 | 0 | 1 | 0 | - | 1 | 2 | 3 | 1 | 0 | 1 | 0 |
| ***Trad.* sp. 2** | 1/2 | 0 | 0 | 0 | 0 | 2 | 0 | 0 | - | - | 0 | 1 | 1 | 1 | 0 | 1 | 0 | 1 | 0 | - | 1 | 2 | 3 | 1 | 0 | 1 | 0 |
| ***Trad. schippii*** | 1/2 | 0 | 0 | 0 | 0 | 2 | 0 | 0 | - | - | 0 | 1 | 1 | 1 | 0 | 1 | 0 | 1 | 0 | - | 1 | 2 | 3 | 1 | 0 | 0 | 1 |
| ***Trad. zanonia*** | 1/2 | 0 | 0 | 0 | 0 | 2 | 0 | 0 | - | - | 0 | 1 | 1 | 1 | 0 | 1 | 0 | 1 | 0 | - | 1 | 2 | 3 | 1 | 0 | 0 | 1 |
| ***Trad. commelinoides*** | 1/2 | 0 | 0 | 0 | 0 | 2 | 0 | 0 | - | - | 0 | 1 | 1 | 1 | 0 | 1 | 0 | 1 | 0 | - | 1 | 2 | 3 | 1 | 0 | 0 | 1 |
| ***Trad. gracilima*** | 1/2 | 0 | 0 | 0 | 0 | 2 | 0 | 0 | - | - | 0 | 1 | 1 | 1 | 0 | 1 | 0 | 1 | 0 | - | 1 | 2 | 3 | 1 | 0 | 0 | 1 |
| ***Trad. grantii*** | 1/2 | 0 | 0 | 0 | 0 | 2 | 0 | 0 | - | - | 0 | 1 | 1 | 1 | 0 | 1 | 0 | 1 | 0 | - | 1 | 2 | 3 | 1 | 0 | 0 | 1 |
| ***Trad. poelliae*** | 1/2 | 0 | 0 | 0 | 0 | 2 | 0 | 0 | - | - | 0 | 1 | 1 | 1 | 0 | 1 | 0 | 1 | 0 | - | 1 | 2 | 3 | 1 | 0 | 0 | 1 |
| ***Trad. praetermissa*** | 1/2 | 0 | 0 | 0 | 0 | 2 | 0 | 0 | - | - | 0 | 1 | 1 | 1 | 0 | 1 | 0 | 1 | 0 | - | 1 | 2 | 3 | 1 | 0 | 0 | 1 |
| ***Trad. standleyi*** | 1/2 | 0 | 0 | 0 | 0 | 2 | 0 | 0 | - | - | 0 | 1 | 1 | 1 | 0 | 1 | 0 | 1 | 0 | - | 1 | 2 | 3 | 1 | 0 | 0 | 1 |
| ***Trad. guatemalensis*** | 1/2 | 0 | 0 | 0 | 0 | 2 | 0 | 0 | - | - | 0 | 1 | 1 | 1 | 0 | 1 | 0 | 1 | 0 | - | 1 | 2 | 3 | 1 | 0 | 0 | 0 |
| ***Trad. soconuscana*** | 1/2 | 0 | 0 | 0 | 0 | 2 | 0 | 0 | - | - | 0 | 1 | 1 | 1 | 0 | 1 | 0 | 1 | 0 | - | 1 | 2 | 3 | 1 | 0 | 0 | 1 |
| ***Trad. ambigua*** | 1/2 | 0 | 0 | 0 | 0 | 2 | 0 | 0 | - | - | 0 | 1 | 1 | 1 | 0 | 1 | 0 | 1 | 0 | - | 1 | 2 | 3 | 1 | 0 | 1 | 0 |
| ***Trad. boliviana*** | 1/2 | 0 | 0 | 0 | 0 | 2 | 0 | 0 | - | - | 0 | 1 | 1 | 1 | 0 | 1 | 0 | 1 | 0 | - | 1 | 2 | 3 | 1 | 0 | 1 | 0 |
| ***Trad. crassifolia*** | 1/2 | 0 | 0 | 0 | 0 | 2 | 0 | 0 | - | - | 0 | 1 | 1 | 1 | 0 | 1 | 0 | 1 | 0 | - | 1 | 2 | 3 | 1 | 0 | 1 | 0 |
| ***Trad. gentryi*** | 1/2 | 0 | 0 | 0 | 0 | 2 | 0 | 0 | - | - | 0 | 1 | 1 | 1 | 0 | 1 | 0 | 1 | 0 | - | 1 | 2 | 3 | 1 | 0 | 1 | 0 |
| ***Trad. petricola*** | 1/2 | 0 | 0 | 0 | 0 | 2 | 0 | 0 | - | - | 0 | 1 | 1 | 1 | 0 | 1 | 0 | 1 | 0 | - | 1 | 2 | 3 | 1 | 0 | 1 | 0 |
| ***Trad. tepoxtlana*** | 1/2 | 0 | 0 | 0 | 0 | 2 | 0 | 0 | - | - | 0 | 1 | 1 | 1 | 0 | 1 | 0 | 1 | 0 | - | 1 | 2 | 3 | 1 | 0 | 1 | 0 |
| ***Trad. andrieuxii*** | 1/2 | 0 | 0 | 0 | 0 | 2 | 0 | 0 | - | - | 0 | 1 | 1 | 1 | 0 | 1 | 0 | 1 | 0 | - | 1 | 2 | 3 | 1 | 0 | 1 | 0 |
| ***Trad. spathacea*** | 1/2 | 0 | 0 | 0 | 0 | 2 | 0 | 0 | - | - | 0 | 1 | 1 | 1 | 0 | 1 | 0 | 1 | 0 | - | 1 | 2 | 3 | 1 | 0 | 0 | 0 |
| ***Trad. virginiana*** | 1/2 | 0 | 0 | 0 | 0 | 2 | 0 | 0 | - | - | 0 | 1 | 1 | 1 | 0 | 1 | 0 | 1 | 0 | - | 1 | 2 | 3 | 1 | 0 | 0 | 1 |
| ***Trad. occidentalis*** | 1/2 | 0 | 0 | 0 | 0 | 2 | 0 | 0 | - | - | 0 | 1 | 1 | 1 | 0 | 1 | 0 | 1 | 0 | - | 1 | 2 | 3 | 1 | 0 | 0 | 1 |
| ***Trad. sillamontana*** | 1/2 | 0 | 0 | 0 | 0 | 2 | 0 | 0 | - | - | 0 | 1 | 1 | 1 | 0 | 1 | 0 | 1 | 0 | - | 1 | 2 | 3 | 1 | 0 | 1 | 0 |
| ***Trad. pinetorum*** | 1/2 | 0 | 0 | 0 | 0 | 2 | 0 | 0 | - | - | 0 | 1 | 1 | 1 | 0 | 1 | 0 | 1 | 0 | - | 1 | 2 | 3 | 1 | 0 | 0 | 1 |
| ***Trad. wrightii*** | 1/2 | 0 | 0 | 0 | 0 | 2 | 0 | 0 | - | - | 0 | 1 | 1 | 1 | 0 | 1 | 0 | 1 | 0 | - | 1 | 2 | 3 | 1 | 0 | 0 | 1 |
| ***Trad. orchidophylla*** | 1/2 | 0 | 0 | 0 | 0 | 2 | 0 | 0 | - | - | 0 | 1 | 1 | 1 | 0 | 1 | 0 | 1 | 0 | - | 1 | 2 | 3 | 1 | 0 | 1 | 0 |
| ***Trad. mirandae*** | 1/2 | 0 | 0 | 0 | 0 | 2 | 0 | 0 | - | - | 0 | 1 | 1 | 1 | 0 | 1 | 0 | 1 | 0 | - | 1 | 2 | 3 | 1 | 0 | 1 | 0 |
| ***Trad. pygmaea*** | 1/2 | 0 | 0 | 0 | 0 | 2 | 0 | 0 | - | - | 0 | 1 | 1 | 1 | 0 | 1 | 0 | 1 | 0 | - | 1 | 2 | 3 | 1 | 0 | 0 | 0 |
| ***Trad. brevifolia*** | 1/2 | 0 | 0 | 0 | 0 | 2 | 0 | 0 | - | - | 0 | 1 | 1 | 1 | 0 | 1 | 0 | 1 | 0 | - | 1 | 2 | 3 | 1 | 0 | 1 | 0 |
| ***Trad. hirta*** | 1/2 | 0 | 0 | 0 | 0 | 2 | 0 | 0 | - | - | 0 | 1 | 1 | 1 | 0 | 1 | 0 | 1 | 0 | - | 1 | 2 | 3 | 1 | 0 | 1 | 0 |
| ***Trad. pallida*** | 1/2 | 0 | 0 | 0 | 0 | 2 | 0 | 0 | - | - | 0 | 1 | 1 | 1 | 0 | 1 | 0 | 1 | 0 | - | 1 | 2 | 3 | 1 | 0 | 1 | 0 |
| ***Trad. zebrina*** | 1/2 | 0 | 0 | 0 | 0 | 2 | 0 | 0 | - | - | 0 | 1 | 1 | 1 | 0 | 1 | 0 | 1 | 0 | - | 1 | 2 | 3 | 1 | 0 | 1 | 1 |
| ***E. hirsuta*** | 1/2 | 0 | 0 | 0 | 0 | 2 | 0 | 0 | - | - | 0 | 1 | 1 | 1 | 0 | 1 | 0 | 1 | 0 | - | 1 | 2 | 3 | 1 | 0 | 0 | 0 |
| ***G. consobrina*** | 1/2 | 0 | 0 | 0 | 0 | 2 | 0 | 0 | - | - | 0 | 1 | 1 | 1 | 0 | 1 | 0 | 1 | 0 | - | 1 | 2 | 3 | 1 | 0 | 0 | 1 |
| ***G. geniculata*** | 1/2 | 0 | 0 | 0 | 0 | 2 | 0 | 0 | - | - | 0 | 1 | 1 | 1 | 0 | 1 | 0 | 1 | 0 | - | 1 | 2 | 3 | 1 | 0 | 0 | 1 |
| ***G. oxacana*** | 1/2 | 0 | 0 | 0 | 0 | 2 | 0 | 0 | - | - | 0 | 1 | 1 | 1 | 0 | 1 | 0 | 1 | 0 | - | 1 | 2 | 3 | 1 | 0 | 0 | 1 |
| ***G. pellucida*** | 1/2 | 0 | 0 | 0 | 0 | 2 | 0 | 0 | - | - | 0 | 1 | 1 | 1 | 0 | 1 | 0 | 1 | 0 | - | 1 | 2 | 3 | 1 | 0 | 0 | 1 |
| ***G. karwinskyana*** | 1/2 | 0 | 0 | 0 | 0 | 2 | 0 | 0 | - | - | 0 | 1 | 1 | 1 | 0 | 1 | 0 | 1 | 0 | - | 1 | 2 | 3 | 1 | 0 | 0 | 1 |
| ***C. filiformis*** | 1/2 | 0 | 0 | 0 | 0 | 2 | 0 | 0 | - | - | 0 | 1 | 1 | 1 | 0 | 1 | 0 | 1 | 0 | - | 1 | 2 | 3 | 1 | 0 | 0 | 1 |
| ***C. fragrans*** | 1/2 | 0 | 0 | 0 | 0 | 2 | 0 | 0 | - | - | 0 | 1 | 1 | 1 | 0 | 1 | 0 | 1 | 0 | - | 1 | 2 | 3 | 1 | 0 | 0 | 0 |
| ***C. gentlei*** | 1/2 | 0 | 0 | 0 | 0 | 2 | 0 | 0 | - | - | 0 | 1 | 1 | 1 | 0 | 1 | 0 | 1 | 0 | - | 1 | 2 | 3 | 1 | 0 | 0 | 0 |
| ***C. monandra*** | 1/2 | 0 | 0 | 0 | 0 | 2 | 0 | 0 | - | - | 0 | 1 | 1 | 1 | 0 | 1 | 0 | 1 | 0 | - | 1 | 2 | 3 | 1 | 0 | 0 | 0 |
| ***C. repens*** | 1/2 | 0 | 0 | 0 | 0 | 2 | 0 | 0 | - | - | 0 | 1 | 1 | 1 | 0 | 1 | 0 | 1 | 0 | - | 1 | 2 | 3 | 1 | 0 | 0 | 0 |
| ***Trip. diuretica*** | 1/2 | 0 | 0 | 0 | 0 | 2 | 0 | 0 | - | - | 0 | 1 | 1 | 1 | 0 | 1 | 0 | 1 | 0 | - | 1 | 2 | 3 | 1 | 0 | 0 | 1 |
| ***Trip. elata*** | 1/2 | 0 | 0 | 0 | 0 | 2 | 0 | 0 | - | - | 0 | 1 | 1 | 1 | 0 | 1 | 0 | 1 | 0 | - | 1 | 2 | 3 | 1 | 0 | 0 | 1 |
| ***Trip. glandulosa*** | 1/2 | 0 | 0 | 0 | 0 | 2 | 0 | 0 | - | - | 0 | 1 | 1 | 1 | 0 | 1 | 0 | 1 | 0 | - | 1 | 2 | 3 | 1 | 0 | 0 | 1 |
| ***Trip. multiflora*** | 1/2 | 0 | 0 | 0 | 0 | 2 | 0 | 0 | - | - | 0 | 1 | 1 | 1 | 0 | 1 | 0 | 1 | 0 | - | 1 | 2 | 3 | 1 | 0 | 0 | 1 |
| ***Trip. warmingiana*** | 1/2 | 0 | 0 | 0 | 0 | 2 | 0 | 0 | - | - | 0 | 1 | 1 | 1 | 0 | 1 | 0 | 1 | 0 | - | 1 | 2 | 3 | 1 | 0 | 0 | 1 |
